# Origin of exponential growth in nonlinear reaction networks

**DOI:** 10.1073/pnas.2013061117

**Published:** 2020-10-22

**Authors:** Wei-Hsiang Lin, Edo Kussell, Lai-Sang Young, Christine Jacobs-Wagner

**Affiliations:** ^a^Microbial Science Institute, Yale University, West Haven, CT 06516;; ^b^Department of Molecular, Cellular and Developmental Biology, Yale University, New Haven, CT 06511;; ^c^Howard Hughes Medical Institute, Yale University, New Haven, CT 06510;; ^d^Department of Biology, Stanford University, Palo Alto, CA 94305;; ^e^Chemistry, Engineering & Medicine for Human Health (ChEM-H) Institute, Stanford University, Palo Alto, CA 94305;; ^f^Center of Genomics and Systems Biology, Department of Biology, New York University, New York, NY 10003;; ^g^Department of Physics, New York University, New York, NY 10003;; ^h^Courant Institute of Mathematical Science, New York University, New York, NY 10012;; ^i^School of Mathematics, Institute for Advanced Study, Princeton, NJ 08540;; ^j^School of Natural Sciences, Institute for Advanced Study, Princeton, NJ 08540;; ^k^Department of Microbial Pathogenesis, Yale School of Medicine, New Haven, CT 06510

**Keywords:** exponential growth, reaction networks, systems biology, ergodic theory

## Abstract

Exponentially growing systems are prevalent in nature, spanning all scales from biochemical reaction networks in single cells to food webs of ecosystems. How exponential growth emerges in nonlinear systems is mathematically unclear. Here, we describe a general theoretical framework that reveals underlying principles of long-term growth: scalability of flux functions and ergodicity of the rescaled systems. Our theory shows that nonlinear fluxes can generate not only balanced growth but also oscillatory or chaotic growth modalities, explaining nonequilibrium dynamics observed in cell cycles and ecosystems. Our mathematical framework is broadly useful in predicting long-term growth rates from natural and synthetic networks, analyzing the effects of system noise and perturbations, validating empirical and phenomenological laws on growth rate, and studying autocatalysis and network evolution.

## Significance

Natural systems (e.g., cells and ecosystems) generally consist of reaction networks (e.g., metabolic networks or food webs) with nonlinear flux functions (e.g., Michaelis–Menten kinetics and density-dependent selection). Despite their complex nonlinearities, these systems often exhibit simple exponential growth in the long term. How exponential growth emerges from nonlinear networks remains elusive. Our work demonstrates mathematically how two principles, multivariate scalability of flux functions and ergodicity of the rescaled system, guarantee a well-defined growth rate. By connecting ergodic theory, a powerful branch of mathematics, to the study of growth in biology, our theoretical framework can recapitulate various growth modalities (from balanced growth to periodic, quasi-periodic, or even chaotic behaviors), greatly expanding the types of growing systems that can be studied.

Reaction networks are fundamental structures of living systems. They describe biochemical networks (consisting of metabolites, chemical reactions, and macromolecules), ecological networks (based on species and foraging activities), and economic systems (composed of materials and production processes) (1–5). Such networks can exhibit a capacity for growth under suitable conditions. A growing system requires uptake of external components, conversion reactions between internal components, and energy-generating pathways. These processes often involve nonlinear dependencies (3). For example, in cells, many biochemical reactions (e.g., Michaelis–Menten kinetics, feedback inhibition, and promoter activation) translate into nonlinear differential equations. Yet, the entire system (total biomass) often converges to exponential growth in the long term, which is a typical property of linear differential equations. Similarly, multispecies communities can often undergo exponential expansion (6, 7). Yet, many interactions in ecosystems, including density-dependent competition and mutation-selection processes, follow nonlinear equations (4). The emergence of exponential growth from a multivariable nonlinear network is not mathematically intuitive. This indicates that the network structure and the flux functions of the modeled system must be subjected to constraints to result in long-term exponential dynamics.

**Fig. 1.**
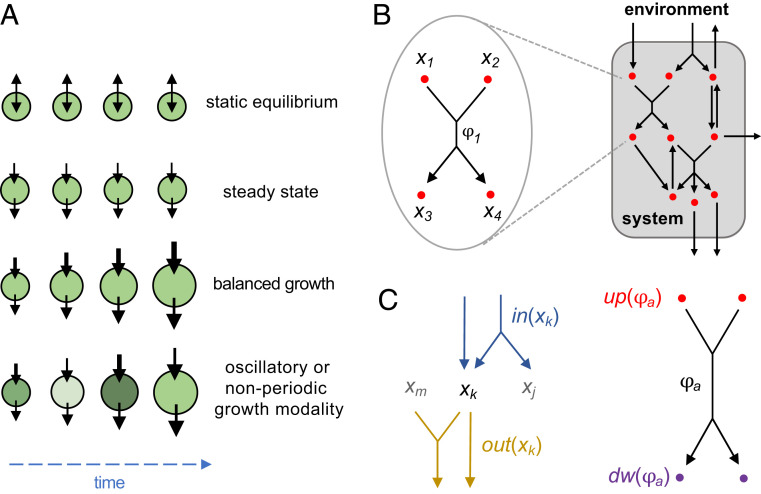
Mathematical formulation of an SRN. (*A*) Growth modalities for various types of open systems. System size is represented by different sizes of circles; a darker green color represents higher metabolite concentration. Flux magnitude is indicated by the thickness of the black arrows. Static equilibrium indicates that the system is at equilibrium with the environment, with the net flux magnitude equal to zero. Steady state means that influx and efflux are of the same magnitude, and the system is maintained at steady state with no growth. Balanced growth indicates that the influx is greater than the efflux, and the system is growing proportionally to the fluxes. For oscillatory and nonperiodic growth modalities, the influx is greater than the efflux and the system is growing, but the relative proportions of influx and efflux, as well as the metabolite concentration, can vary over time. (*B*) A reaction network is composed of a collection of nodes and reactions. Each reaction describes an interconversion process between upstream and downstream nodes and between these nodes and the environment. (*C*) Terminology describing relations between node x and reaction ϕ. Given a node $x_{k}$, the collections of all reactions having $x_{k}$ as the upstream and downstream nodes are denoted by $in\left(x_{k}\right)$ and $out\left(x_{k}\right),$ respectively. Given a reaction $\phi_{a}$, the collections of all upstream and downstream nodes are denoted by $up\left(\phi_{a}\right)$ and $dw\left(\phi_{a}\right)$.

Current growth models, including those based on flux balance analysis (8), proteome partition analysis (9), and general reaction networks (10–12), have been successful at predicting biomass growth. A common limitation of these models is that they assume a priori that the system has long-term exponential growth. In addition, they generally assume that the system exhibits balanced growth (Fig. 1*A*). That is, all components of the system under study are assumed to exponentially increase in amount at the same constant rate, such that their ratios remain fixed during growth of the system. However, in many biological systems, the components are not in balance and display oscillatory or even nonperiodic, behaviors caused by nonlinear fluxes in the systems. For example, the levels of metabolites and proteins can oscillate during cell growth (13–15). The abundance of species in ecosystems can also oscillate (6) or exhibit nonperiodic fluctuations (7). Yet, the biomass of these systems increases exponentially (6, 7, 16). This raises a fundamental question: Are there general principles that underlie long-term exponential growth in complex reaction networks?

Addressing these questions has been a major challenge due to the lack of a generalizable theoretical framework that goes beyond balanced growth. Ergodic theory is a powerful mathematical tool that has been successfully applied in the fields of fluid dynamics and statistical mechanics for studying the long-term behavior of physical systems (17). The ergodic condition is met when the time average of a quantity is equal to a statistical average over all possible outcomes weighed by their likelihood, also known as a space average. For example, the distance between two children running around a playground independently is a stochastic, time-dependent function, yet the average distance between them over the long term does not depend on time and can be calculated from each child’s probability distribution over space. In reaction networks, the relevant “space” consists of the set of all possible configurations or states of the entire system, and the overall dynamics constitute a time-evolution process in this state space. A basic requirement for the application of ergodic theory is stationarity of the process under consideration, which means that the likelihood of events does not change with time. Biological systems whose biomass increases over time violate stationarity, and ergodic theory, therefore, cannot be applied directly to study their long-term growth properties.

Here, we demonstrate that ergodic theory can be applicable in growing systems whose dynamics may be properly rescaled such that the growth process is decoupled from the rest of the dynamics. We show that many nonlinear biological reaction networks—which we define and refer to as scalable reaction networks (SRNs)—satisfy this condition. By applying ergodic theory on rescaled systems, we show mathematically that scalability and ergodicity ensure that a large class of reaction networks have well-defined long-term growth rates (λ), which can be positive (exponential growth), negative (exponential decay), or zero (subexponential dynamics). This theoretical framework opens the door to the study and characterization of processes that exhibit different growth modalities, including not only static equilibrium, steady state, and balanced growth but also oscillatory and nonperiodic growth (Fig. 1*A*). The theory is applicable both for deterministic and stochastic dynamics. It enables one to construct scalable networks of arbitrarily high complexity, predict the growth rate and other dynamical features of the system, and identify autocatalytic networks associated with positive exponential growth.

## Results

### Reaction Network Modeling and Long-Term Growth Rates.

We consider a system represented by a collection of nodes within an environment, *E*, that serves as an ideal reservoir for unlimited supply and removal of materials. A reaction network is defined by a set of nodes $\left\{x_{1},\ldots ,x_{n}\right\},$ reactions $\left\{\phi_{1},\ldots ,\phi_{m}\right\}$, and flux functions $\left\{J_{1},\ldots ,J_{m}\right\}$, which specify the flux magnitude of each reaction. Each reaction represents an interconversion process that consumes materials from upstream nodes and produces materials in downstream nodes (Fig. 1*B*). The stoichiometric coefficients of interconversion reactions are given by an *n* × *m* stoichiometry matrix S (8) (*SI Appendix*), with positive or negative matrix element $S_{ka}$ indicating that the reaction $\phi_{a}$ acts as an influx or efflux of node $x_{k}$, respectively (Fig. 1*C*). In real-world applications, such networks can describe cellular components (e.g., metabolites and metabolic reactions), cellular populations (e.g., cellular states and transitions), or ecological structures (e.g., species and competitive interactions).

To model the system dynamics, we define a biomass vector $\vec{X}={\left(X_{1},\ldots ,X_{n}\right)}^{T}$ as the total amount of material in each node (in units of mass, e.g., grams), and denote the system size by $N\equiv X_{1}+\ldots +X_{n}$. The reaction flux $J_{a}\left(\vec{X}\right)$ is a multivariate function that specifies the rate of reaction $\phi_{a}$, which can depend on any number of components of $\vec{X}$ and can be highly nonlinear depending on, for example, the order of the reaction, whether or not it is enzyme-catalyzed, and so on. For each node, summing all incoming and outgoing fluxes weighted by the stoichiometric coefficients yields a system of ordinary differential equations that governs the network’s dynamics,

$$\begin{array}{c} \frac{dX_{k}}{dt}=\overset{m}{\underset{a=1}{\sum }}S_{ka}\;J_{a}\left(\vec{X}\right). \end{array}$$ [1]

Such systems of nonlinear equations generally cannot be solved analytically and are typically analyzed using numerical algorithms. Currently, the only way to ensure or verify that a reaction network exhibits long-term exponential growth is to run large-scale numerical computations, combined with massive scans of parameter space, and check the results on a case-by-case basis. We therefore sought to identify fundamental features of nonlinear networks that give rise to exponential growth.

We consider the instantaneous growth rate of the system, μ(t)≡(d/dt)log⁡N(t), a function that specifies the relative rate of change of the population size at time t. If the system grows exponentially at long times, then the long-term time average of μ(t) yields the system’s exponential growth rate,

$$\begin{array}{c} \lambda \equiv \underset{t\to \infty }{\text{lim}}\frac{1}{t}\overset{t}{\underset{0}{\int }}\mu \left(t^{'}\right)\;dt^{'}. \end{array}$$ [2]

Note that the long-term dynamics of Eq. **2** could result in a system size that grows exponentially (λ>0), decays exponentially(λ<0), or displays subexponential dynamics (λ=0). Since most of our discussion is related to growing systems, we will refer to λ as long-term growth rate but the reader should keep in mind that the same formula is equally applicable to systems with λ≤0.

Mathematically, given an arbitrary set of nonlinear reactions there is no guarantee that the time average in Eq. **2** will converge to a well-defined limit. For example, if the instantaneous growth rate increases linearly with time, its long-term average will diverge, or if N(t) decays to zero in a finite amount of time, λ has no well-defined meaning. More complex behaviors can also emerge in reaction networks, such as periodic, quasi-periodic, and chaotic dynamics, and in such cases the relation of the network dynamics to λ is not clear.

We wish to establish general conditions such that the time average given in Eq. **2** converges, implying that the system will grow exponentially. These conditions should 1) ensure that the dynamical system of Eq. **1** has a well-defined solution $\vec{X}\left(t\right)$ for all initial conditions, 2) imply that the system’s composition can exhibit stationarity even as population size may grow without bound, and 3) enable the time average in Eq. **2** to be computed as a space average over the system’s stationary distribution. We examine the system’s biomass composition vector, $\vec{Y}=\vec{X}/N$, whose components specify the fraction of the total biomass present in node $x_{i}$. By substituting $X_{k}=NY_{k}$ into Eq. **1**, we find

$$\begin{array}{c} \frac{dY_{k}}{dt}=\overset{m}{\underset{a=1}{\sum }}S_{ka}\left[J_{a}\left(N\vec{Y}\right)/N\right]-\mu \left(t\right)\;Y_{k}. \end{array}$$ [3]

Here, we see that if all fluxes scale up proportionally with N, that is, if the flux functions are extensive in the system size, then the term within the brackets would be simply $J_{a}\left(\vec{Y}\right)$, and independent of N. Summing both sides over k yields the expression for the growth rate,

$$\begin{array}{c} \mu \left(t\right)=\overset{n}{\underset{k=1}{\sum }}\overset{m}{\underset{a=1}{\sum }}S_{ka}\;J_{a}\left(\vec{Y}\right)\;, \end{array}$$ [4]

which would likewise be independent of N. Then, the system’s composition $\vec{Y}$ evolves independently of N according to the dynamical system in Eq. **3**, and the composition alone determines the instantaneous growth rate according to Eq. **4**. Hence, we can write $\mu \left(t\right)=\mu (\vec{Y}\left(t\right))$ (*SI Appendix*, Fig. S1). Below, we formally specify the conditions that ensure that all fluxes are extensive and that Eq. **3** has a well-defined, physical solution. We will refer to such systems as SRNs.

### Building SRNs: Conditions and Examples.

We state the formal conditions that define an SRN and discuss some practical implications and examples. We denote by $Q^{n+}\equiv \left\{X_{j}>0\right\}$ the positive quadrant and by $Q^{n}\equiv \left\{X_{j}\geq 0\right\}$ the nonnegative quadrant. A flux function $J_{a}\left(\vec{X}\right)$ is scalable if it satisfies three conditions:

1) $J_{a}\left(\vec{X}\right)$ is positive on $Q^{n+}$ and continuously differentiable on $Q^{n}\backslash \left\{0\right\}$;
2) if node $x_{k}$ is an upstream node of $J_{a}\left(X\right)$, then $J_{a}\left(X\right)=0$ whenever $X_{k}=0$; and
3) $J_{a}\left(c\vec{X}\right)=cJ_{a}\left(\vec{X}\right)$ for any c>0.

A reaction network is an SRN if all of its flux functions are scalable.

For an SRN, condition 1 guarantees that the system given in Eq. **1** will have a unique solution. Condition 2 requires the flux functions to be upstream-limited, that is, whenever an upstream node is depleted, its connected efflux must be zero. This ensures that solutions are physical, that is, the trajectories remain within $Q^{n}$. Condition 3 requires the flux magnitude to be extensive in the system size, which enables projection onto the simplex and the application of ergodic theory as detailed below.

We would like to emphasize that a multivariate flux function satisfying condition 3 does not necessarily need to be linear. There is a wide range of commonly occurring flux functions that are not linear and yet scalable (Table 1), which one can verify by checking conditions 1 to 3 directly.

**Table 1.** Common examples of scalable and asymptotically scalable flux functions, where $x_{j},\;x_{k}$ are upstream nodes of the flux, $x_{z}$ is a maintenance node of the flux, and $x_{p}$ is a regulatory node of the flux

Scalable flux functions		Asymptotically scalable flux functions	
$rX_{j}$	r>0	All scalable fluxes	
$rY_{j}^{a}Y_{k}^{b}N$	r>0; a,b≥1	$X_{j}\left(\frac{b_{0}+b_{1}X_{p}+b_{2}X_{p}^{2}}{a_{0}+a_{1}X_{p}+a_{2}X_{p}^{2}}\right)$	$a_{j},\;b_{j}>0$
$\frac{rX_{j}^{\theta }X_{z}}{X_{j}^{\theta }+KN^{\theta }}$	r,K>0; θ≥1	$\left(a+be^{-cX_{p}^{m}}\right)\;X_{j}$	a,b,c>0, m≥1
$\frac{rX_{j}X_{k}}{aX_{j}+bX_{k}+cN}$	r,a, b, c>0	$\left[a+\tan^{-1}\left(bX_{p}\right)\right]X_{j}$	$a>\frac{\pi }{2}$
$\left[a+b\sin\left(\frac{cX_{p}}{N}\right)\right]X_{j}$	a>b>0; c>0	$\left[a+\sin\left(\frac{b+X_{p}}{c+X_{p}}\right)\right]X_{j}$	a>1; b,c>0

The system size is $N=X_{1}+\ldots +X_{n}$.

More generally, biochemical fluxes are typically written as nonlinear functions of metabolite concentrations $\left[\vec{X}\right]$ (mass per volume). If, in addition, the system volume (V) scales with the total biomass N, that is, V=bN with constant b, the fluxes become scalable. To see this, we denote by $J_{unit}\left(\left[\vec{X}\right]\right)$ the metabolic flux magnitude per unit volume. Metabolic fluxes usually obey a mass-action law, for example $J_{unit}\propto {\left[X_{1}\right]}^{A}{\left[X_{2}\right]}^{B}$ or quasi-steady-state kinetics, for example $J_{unit}\propto \frac{\left[X_{1}\right]\left[X_{2}\right]}{K+\left[X_{1}\right]}$. The total flux magnitude $J\left(\vec{X}\right)$ scales with volume and can be expressed as

$$\begin{array}{c} J\left(\vec{X}\right)=VJ_{unit}\left(\left[\vec{X}\right]\right)=bNJ_{unit}\left(\frac{X_{1}}{bN},\ldots ,\frac{X_{n}}{bN}\right)\;, \end{array}$$ [5]

which satisfies the scaling condition 3. Therefore, under the volume–biomass scaling assumption, biochemical flux functions belong to the class of scalable flux functions. When volume–biomass scaling is not exact, a larger class of networks, which we refer to as asymptotically scalable, is often applicable with similar results (see *SI Appendix* and Table 1).

### Ergodicity and Dynamics in SRNs.

We are now positioned to apply ergodic theory to evaluate the time average of Eq. **2**. The scalable condition of SRNs allows us to rescale the system and focus on the long-term dynamics of the composition vector $\vec{Y}\left(t\right)$ in the unit simplex $\Delta^{n-1}$, the space of all vectors ${\left(Y_{1},\ldots ,Y_{n}\right)}^{T}$ satisfying $\overset{n}{\underset{i=1}{\sum }}Y_{i}=1$ with $Y_{i}\geq 0$ for all components i. The distribution on the simplex space, or likelihood of $\vec{Y}\left(t\right)$ being located in a given region of $\Delta^{n-1}$, can be regarded as a probability measure ω defined on $\Delta^{n-1}$, satisfying the normalization condition $\overset{\;}{\underset{\Delta^{n-1}}{\int }}\omega \left(d\vec{Y}\right)=1$. We are interested in probability measures ω that are invariant under the time evolution of Eq. **3**, that is, probability distributions with respect to which $\vec{Y}\left(t\right)$ is stationary. Invariant measures are sometimes decomposable, meaning they are weighted sums of invariant measures supported on disjoint invariant sets. Those that cannot be decomposed are called ergodic measures. Ergodic measures are guaranteed to exist for continuous flows on a compact metric space such as $\Delta^{n-1}$, and they have the important property that time averages of observables can be equated with space averages, a property we now use to compute exponential growth rates.

Given an ergodic measure ω, we can apply the Birkhoff ergodic theorem to evaluate Eq. **2**, yielding

$$\lambda \;\underset{\underset{by\;scalability}{↑}}{=}\overset{\left[\text{time}\;\text{average}\right]}{\underset{t\to \infty }{\text{lim}}\frac{1}{t}\;\overset{t}{\underset{0}{\int }}\mu \left(\vec{Y}\left(t^{\text{'}}\right)\right)\;dt^{\text{'}}}\;\underset{\underset{by\;ergodicity}{↑}}{=}\overset{\left[\text{space}\;\text{average}\right]}{\overset{\;}{\underset{\Delta^{n-1}}{\int }}\mu \left(\vec{Y}\right)\omega \left(d\vec{Y}\right)}\;,$$ [6]

where the second equality holds for almost every initial condition $\vec{Y}\left(0\right)$, except possibly on a set of measure zero with respect to ω. Since $\Delta^{n-1}$is a compact space, $\mu \left(\vec{Y}\right)$ is bounded and hence the space average in Eq. **6** is finite. Thus, the long-term growth rate λ defined in Eq. **2** converges and its value is independent of initial conditions (except for a set of ω-measure zero; see *SI Appendix*, section 2 for an example and a detailed discussion). It is also straightforward to show that if a trajectory exhibits exponential growth, any other trajectory that converges to the first one will have the same long-term growth rate. Thus, depending on the structure of ω, a wide range of trajectories exhibit exponential growth with the same rate.

We illustrate the above point with a few concrete scenarios in low dimensional systems, though it is important to note that the real power of the results is that they hold for high-dimensional SRNs, that is, for networks with an arbitrary number of nodes. Here, we consider three-node SRNs, where the composition vector lies in the two-dimensional simplex $\Delta^{2}$.

**Fig. 2.**
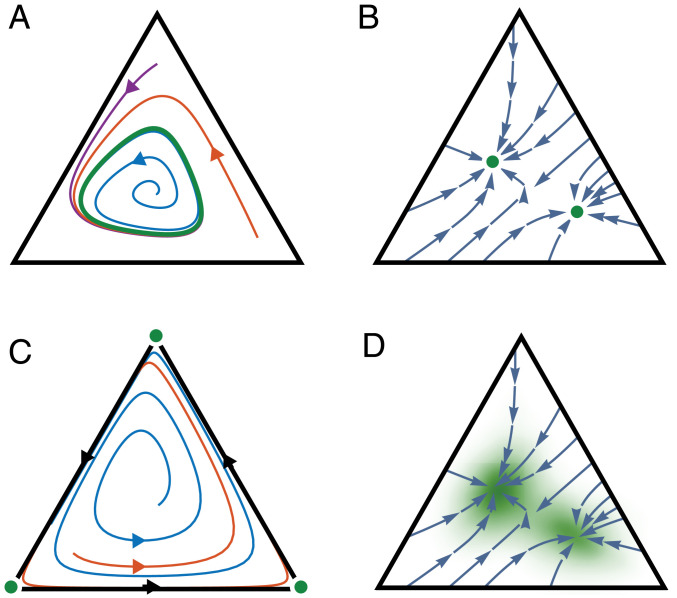
Dynamics of three-node SRNs and ergodic measures. Each panel shows representative trajectories of the composition vector $\vec{Y}\left(t\right)$ on the simplex $\Delta^{2}$, where vertices correspond to network composition states (1,0,0), (0,1,0), or (0,0,1), and any point in the simplex corresponds to a linear combination of these. Ergodic measures are shown in green. (*A*) Dynamics with a stable limit cycle; the ergodic measure is supported on the limit cycle. (*B*) A bistable system with two stable fixed points; two ergodic measures are shown, supported on each of the stable fixed points. (*C*) Dynamics with a heteroclinic cycle on the boundary; ergodic measures exist supported at each of the vertices which are unstable fixed points. Trajectories approach each vertex for a progressively longer time before moving close to the next vertex, and so forth. (*D*) Stochastic dynamics were simulated for the bistable system of *B* and the ergodic measure is shown as a green density within the simplex; the density peaks near the fixed points of the deterministic flows shown in blue.

Let us first discuss deterministic dynamics without noise. Flows exhibiting a limit cycle, bistability, or a heteroclinic cycle are shown in Fig. 2 *A*–*C* (the support of the ergodic measures is shown in green, and any regions lying outside of the support have measure zero). In Fig. 2*A*, the ergodic measure is supported by a limit cycle, and thus λ converges along the limit cycle. All other trajectories converge to the limit cycle trajectory, which implies that the system eventually grows exponentially with the same rate λ regardless of the initial condition. In Fig. 2*B*, we show a bistable system in which two ergodic measures are shown, each supported on a different fixed point. A trajectory that remains at one fixed point will grow exponentially, but the rate of growth can be different for each fixed point; all trajectories that converge to a given fixed point will grow exponentially with the same rate. In Fig. 2*C*, ergodic measures exist at each of the simplex vertices, and a heteroclinic cycle lies on the boundary. Trajectories starting in the interior of the simplex spend progressively longer amounts of time near each of the vertices without converging to any one of them. In this case, although the long-term growth rate would generally not converge, exponential growth will be achieved for increasingly long periods of time while the trajectory remains in the vicinity of each fixed point.

We see that while ergodic measures determine the long-term growth rates of SRNs, complex scenarios involving multiple ergodic measures are possible even in low-dimensional systems, and the situation only gets more complicated in higher dimensions. However, in the presence of random noise, one generally obtains dynamics that are better behaved and are described by a single ergodic measure. All of the ergodic theory results described above carry through for random dynamical systems, specifically when Eq. **3** is formulated as a stochastic differential equation to model noise in the system’s composition. Fig. 2*D* shows the bistable network of Fig. 2*B* with the addition of noise. A single ergodic measure exists on the interior of the simplex, concentrated near the two fixed points but also exhibiting nonzero measure between them, indicating that stochastic trajectories spend time sampling both fixed points. Thus, an SRN whose deterministic dynamics exhibit distinct growth rates dependent on the initial conditions can, due to the presence of noise, grow exponentially with a single rate for all trajectories. The timescale for a stochastic trajectory to converge to an ergodic measure depends on the magnitude of diffusion and drift on the simplex (18). Mathematical techniques, such as large deviations theory and mean first passage time calculations (e.g., refs. 19 and 20), can be applied for estimating the timescale for convergence.

In summary, ergodic measures underlie exponential growth modalities in SRNs. They can be used to analyze these modalities for both deterministic and stochastic dynamics. We found two general criteria for the long-term growth rate of an SRN to converge (see *SI Appendix*, *Supplementary Text* for proof):

• For deterministic dynamics, if a trajectory $\vec{Y}\left(t\right)$ converges to a fixed point, a periodic or a quasiperiodic orbit, then it exhibits a well-defined long-term growth rate λ, which is determined by the ergodic measure for the given attractor according to Eq. **6**.
• For stochastic dynamics, if one adds random diffusion to the composition vector, and if SRNs can always regenerate any component that goes to zero from other components (we call these regenerative SRNs), then all trajectories have the same exponential growth rate regardless of the initial conditions.

Our results extend also to chaotic dynamics, as we show below. Further details and generalizations are provided in *SI Appendix*. In the following sections, we use concrete examples to illustrate the versatility of the SRN framework. This framework allows us to implement nonlinear flux functions in reaction networks to study growth dynamics beyond balanced growth and perform systematic parameter scans for long-term growth rate analysis.

### Growth Modalities of SRNs.

SRNs that display exponential growth can exhibit various growth modalities. The simplest is balanced growth, in which all components of the system grow at the same unchanging rate (Fig. 1*A*). This modality corresponds to exponential growth along a constant vector ${\vec{Y}}^{*}$, and according to Eq. **6** it is a special case in which an ergodic measure is concentrated entirely at ${\vec{Y}}^{*}$, that is, $\omega =\delta \left(\vec{Y}-{\vec{Y}}^{*}\right)$ and $\lambda =\mu \left({\vec{Y}}^{*}\right)$. In general, ω can have forms that correspond to various growth modalities. They include periodic growth as in metabolic cycles (14, 21), cell cycles (22–24), oscillating populations (6, 25), and economic business cycles (26) as well as different types of aperiodic growth such as chaos in microbial food webs (7). Such systems cannot be modeled according to a balanced growth assumption, yet their ability to exhibit exponential growth over long timescales follows the general principles of ergodicity and scalability described above.

**Fig. 3.**
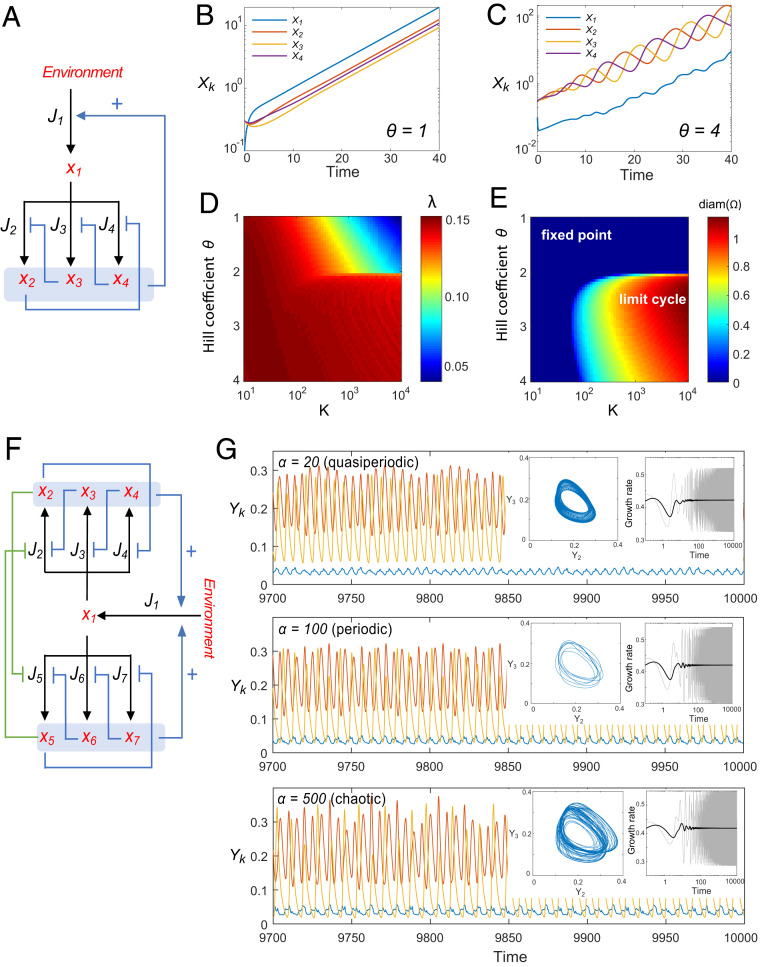
SRNs with various growth modes. (*A*) An autocatalytic, single-repressilator SRN. Blue lines ending with an arrow represent flux activation, whereas blue lines ending with bars indicate flux repression. The flux functions $J_{2}$,$J_{3},J_{4}$have sigmoidal forms, for example $J_{2}\left(X\right)=\frac{cX_{1}}{1+K{\left(X_{3}/N\right)}^{\theta }}$. (*B*) Balanced growth (θ=1, K=500) for the network shown in *A*. (*C*) Periodic growth modality (θ=3,K=500) for the network shown in *A*. (*C*) Phase diagram of λ in the parameter space (θ,K) for the network shown in *A*. (*E*) Phase diagram of the diameter (diam) of the attractor (Ω) for the network shown in *A*. In this regime, Ω is either a fixed point or a limit cycle in $\Delta^{3}$. The diameter of Ω is defined by $\underset{Y,Y'\in \text{Ω}}{\max}|Y-Y'|$; it is zero for fixed points and positive for limit cycles. (*F*) An autocatalytic double repressilator SRN. The parameter α is the ratio of repression strengths between the two oscillators (see *SI Appendix* for details). (*G*) Growth modalities of the double repressilator. Dynamics can be quasiperiodic (α=20), periodic (α=100), or chaotic (α=500). (*Left Insets*) Projection of attractors on $Y_{2}-Y_{3}$ coordinates. (*Right Insets*) Instantaneous growth rate μ(t) (gray) and long-term average $\left(1/t\right)\overset{t}{\underset{0}{\int }}\mu \left(t'\right)dt'$ (black).

To demonstrate the utility of SRNs for the study of nonbalanced growth, we consider two explicit examples with various growth modalities for which long-term growth rates cannot be calculated by using a balanced-growth approach. We first constructed an SRN consisting of a repressilator-type regulatory circuitry. The repressilator is an autonomous nonlinear genetic circuit that is well-known for generating oscillations of protein expression in bacterial cells (27). To create a growing system, we incorporated the repressilator circuit into an autocatalytic network (Fig. 3*A*). In this model, the synthesis fluxes $J_{2},\;J_{3},$ and $J_{4}$ are subject to the repression of nodes $x_{3},\;x_{4},$ and $x_{2}$, respectively. These sigmoidal flux functions depend on the Hill coefficient (θ) and the repression strength (K). By varying θ, we found that this network is able to change growth modes. For small θ (e.g., θ= 1), the system exhibits balanced growth (Fig. 3*B*),while larger θ (e.g., θ = 4) causes the system to display a periodic growth modality (Fig. 3*C*). Here, the bifurcation happens around θ≈2 upon a change in the fixed-point stability of Eq. **3**. Interestingly, the oscillation period and growth rate are strongly coupled in this model. For example, under high repression strength, growth rate λ sharply increases around θ≈2 (Fig. 3*D*), where the attractor of $\vec{Y}\left(t\right)$ transitions from a fixed point to a limit cycle (Fig. 3*E*). The transition from fixed point toward limit cycle is accompanied by a fast increase of growth rate. This intriguing behavior is due to a change in the network composition at the transition, such that the relative biomass fraction of nodes $x_{2},\;x_{3},\;x_{4}$ sharply increases, promoting greater autocatalysis influx ($J_{1}$ in Fig. 3*A*), which yields higher values of λ.

By combining two mutually inhibiting repressilator networks (Fig. 3*F*) we obtained an SRN capable of exhibiting additional growth modalities, ranging from quasiperiodic to nonperiodic, chaotic growth (Fig. 3*G*; see *SI Appendix*, Fig. S3 for the full bifurcation diagram and the largest Lyapunov exponent signature). Under a chaotic growth modality, the trajectory of $\vec{Y}\left(t\right)$ is intrinsically unpredictable in the long term. However, in the presence of noise, this regenerative SRN is guaranteed to have a single ergodic measure with density in the interior of the simplex, and therefore a well-defined long-term growth rate, which we calculate using Eq. **6** in the small-noise limit (*SI Appendix*, Fig. S3*C*). Numerical simulation indicates convergence of λ (Fig. 3 *G*, *Insets*). This example demonstrates that the long-term growth rate of SRNs can be robust, even when the components of the network fluctuate indefinitely.

### Exponential Growth of Ecosystems with Interspecies Competition and Cross-Feeding.

In ecological and population dynamic contexts, SRNs provide a basis for understanding how exponential growth can emerge from nonlinear interactions between different species, such as in natural expansion of ecosystems. In addition, the SRN model is also applicable for turbidostat and chemostat experiments. In a turbidostat, nutrients typically remain in excess at all times and cell density is maintained constant by a feedback loop that adjusts the dilution rate. In such a bioreactor, the long-term average dilution rate is equal to the long-term growth rate of the population and therefore can be predicted by the SRN model. In a chemostat, an essential nutrient is in limiting concentration while the dilution rate is held at a constant value set by the user. We provide an SRN model that enables the long-term dynamics of chemostats to be analyzed under the SRN framework (*SI Appendix*, section 2.8).

**Fig. 4.**
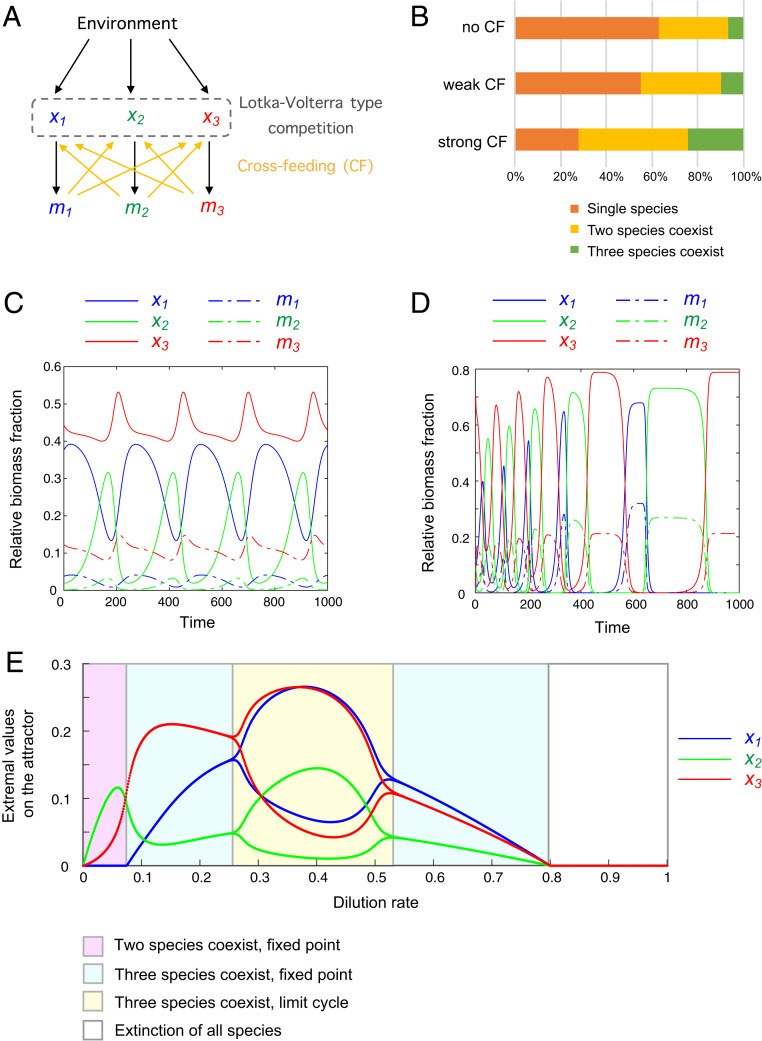
Models of three species that mutually compete and cross-feed under the condition of unlimited (turbidostat-like) or limited (chemostat-like) external resources. See *SI Appendix, Methods* for model details. (*A*) Cross-feeding reaction network with three species ($x_{1}$ to $x_{3}$) and three secreted metabolites ($m_{1}$ to $m_{3}$). The Lotka–Volterra-type competition is described by quadratic functions, and the cross-feeding fluxes follow Michaelis–Menten functions. (*B*) Species coexistence statistics when cross-feeding is absent, weak (cross-feeding efficiency between 0 and 0.1), or strong (cross-feeding efficiency between 0 and 0.5) for a turbidostat-type system. The three-species coexistence includes growth modalities of a fixed point, a limit cycle and a heteroclinic cycle. (*C*) An example from *B* where the cross-feeding system has a limit cycle growth modality. (*D*) An example from *B* where the cross-feeding system has a heteroclinic cycle growth modality. (*E*) Long-term growth dynamics of the cross-feeding population (shown in *A*) for a chemostat-type system as a function of the dilution rate, D.

To illustrate these points, we used an SRN framework to construct a three-species community in which the species compete through a classical Lotka–Volterra-type interaction (28) (Fig. 4*A*). In addition, in this model, each species ($x_{1}$ to $x_{3}$) secretes a metabolite ($m_{1}$ to $m_{3}$) that can cross-feed the other two species. The efficiency of cross-feeding and the competitive interactions are allowed to be asymmetric. To mimic the diversity of natural interactions among species, we took advantage of the SRN framework to scan 150,000 different parameter sets of species interactions and studied long-term species coexistence (*SI Appendix*, *Methods*).

We initially performed our analysis assuming that the environmental nutrient is unlimited (turbidostat-type systems). For a first dataset, we simulated random competitive interactions without cross-feeding. We found that, in the long term, more than 60% of the simulated systems consist of single species, with the remaining systems consisting of two or all three species (Fig. 4*B*). For a second dataset, we simulated random competitive interactions plus random weak cross-feeding (with efficiency parameters between 0 and 0.1; see *SI Appendix*, *Methods*). Increasing the efficiency of cross-feeding increased the chance of species to coexist (Fig. 4*B*). For a third dataset, we allowed stronger cross-feeding to occur (with efficiency parameter between 0 and 0.5), which further increased the probability of species coexistence (Fig. 4*B*). We found that different growth modalities can emerge when three species coexist. They include not only examples of balanced growth (fixed point) but also examples of limit cycles (periodic growth, Fig. 4*C*) and heteroclinic cycles (growth with increasing period, Fig. 4*D*). As shown mathematically (see above and *SI Appendix*), a rescaled system with a fixed point or limit cycle has a well-defined growth rate. In the case of an ecosystem with a heteroclinic cycle, one of the three species will dominate alternately with increasing periods. Exponential growth is achieved when the trajectory Y(t) is at the vicinity of the saddle points of the heteroclinic cycle. For deterministic dynamics the heteroclinic cycle can be arbitrarily close to the simplex boundary. In stochastic dynamics of finite populations, a species would go extinct when the last individual is lost, and the system could end up at a fixed point.

Next, we considered the case when the concentration of a nutrient is limiting (chemostat-type systems) by modifying the cross-feeding model to a chemostat-type equation and including the limited nutrient into the equation (*SI Appendix*, section 2.8). By scanning 120,000 parameter sets, we found that the cross-feeding model can, in specific cases, exhibit transitions in growth modality as the dilution rate is varied. This is illustrated in Fig. 4*E*, where the long-term dynamics can be either two-species fixed point, three-species fixed point, three-species limit cycle, or extinction depending on the dilution rate D.The bifurcations of this system can therefore be studied using the SRN framework in the future.

Our results provide a theoretical basis to understand how ecosystems can grow and expand exponentially even when exhibiting complex, unbalanced dynamics among not only species but also resources (e.g., due to cross-feeding or nutrient limitation). We envision that similar SRN-based models can be constructed for economic systems to study their growth [e.g., business cycles (26)].

### Exponential Growth of Biosynthesis with Energy Allocation.

**Fig. 5.**
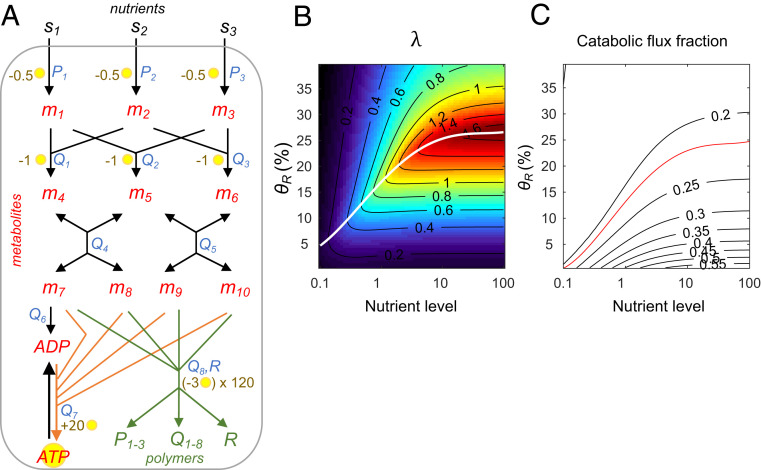
Toy model of an autocatalytic biosynthesis network. (*A*) Diagram of the biosynthesis network. Nodes labeled in red and green correspond to metabolites and polymers, respectively. The external resources $s_{1},\;s_{2},s_{3}$ are imported into the system and converted into metabolites $m_{1}$ to $m_{6}$ through a series of reactions. These metabolites are used to produce amino acids $m_{7}$ to $m_{10}$, which can either enter anabolic pathways (green arrows) to produce polymers such as transporters ($P_{1-3}$), enzymes ($Q_{1-8}$), and ribosomes (*R*) or enter catabolic pathways (orange arrows) to replenish cellular energy in the form of ATP from ADP. Text in blue next to a reaction represents the catalytic enzyme of this flux. The yellow circle next to a reaction represents the number of ATP produced or consumed by this reaction. For details about the model and flux functions, see *SI Appendix*. (*B*) Growth rate under different ribosomal synthesis strengths ($\theta_{R}$) and varying external nutrient levels (set to be the same level for $s_{1}=s_{2}=s_{3}$). The white line indicates the optimal growth condition under each external nutrient level. (*C*) Contour line of the catabolic flux fraction of amino acids ($m_{7-10}$). The red line indicates the optimal condition predicted by the objective function (see main text).

Often reaction networks are modeled to study cellular metabolism and biomass growth, which can be done using SNRs. As a proof of concept, we constructed a simplified biosynthesis model with 24 nodes using common metabolic flux functions such as Michaelis–Menten equations (Fig. 5*A*; also see *SI Appendix* for details). The advantage of using an SRN framework is that it enables unconstrained parameter scans of the system while automatically ensuring that various unphysical behaviors are avoided (e.g., the system size blowing up at a finite time, or biomass components becoming negative). There is no need to impose constraints based on empirical objective functions or phenomenological laws. Instead, as we illustrate below, the SRNs model can be used to uncover a phenomenological law.

We built a toy model in which the reaction network imports three types of external nutrients ($s_{1}$ to $s_{3}$) and converts them to amino acids ($m_{7}$ to $m_{10}$) through intermediate metabolites ($m_{1}$ to $m_{6}$) (Fig. 5*A*). The generated amino acids are then utilized in polymer synthesis (anabolic pathways, green) or energy production (catabolic pathways, orange). Biosynthesis produces transporters (*P*_1–3_), enzymes (*Q*_1–8_), and ribosomes (*R*), while energy production replenishes adenosine 5′-triphosphate (ATP) from adenosine 5′-diphosphate (ADP). The polymers and energy molecules are, in turn, required for catalyzing the upstream fluxes (see *SI Appendix*, *Methods* section 4 for details). Using parameter values that are compatible with physiological conditions (*SI Appendix*, Table S4), the system trajectory Y(t) converges to a fixed point or a limit cycle. Hence, the long-term growth rate can be calculated using Eq. **6**, and the correlation between growth rate, metabolic levels, and flux magnitudes can be analyzed for various parameters.

Using this model, we investigated how growth rate is affected by the balance between catabolism and anabolism, which is known to be essential to achieve optimal biosynthesis (29, 30). We set values of ATP consumption and production within a reasonable range (31). Specifically, consumption of one amino acid ($m_{7-10}$) produces 20 ATP while the synthesis of an amino acid costs 2 ATP. Utilization of amino acids for polymer synthesis costs 3 ATP. Therefore, for each amino acid, the system either produces 18 ATP through the catabolic pathway (orange arrows) or consumes 5 ATP through the anabolic pathway (green arrows) for polymer synthesis (Fig. 5*A*). If the flux magnitudes of the catabolic and anabolic pathways have a ratio of 5:18, the production and consumption of ATP will be balanced. Heuristically, 5/(5 + 18) = 0.22 would be the optimal catabolic flux fraction. This value can be used as a target for an objective function on energy balance.

To determine whether this objective function predicts the optimal growth rate, we varied the external nutrient level and the ribosomal synthesis strength in simulations. In Fig. 5*B*, the condition with the highest λ across various nutrient levels is indicated by the white curve (left graph). In Fig. 5*C*, the optimal objective function (catabolic flux fraction = 0.22) is given by the red curve. The similarity between the white and red curves shows that the objective function can indeed predict optimal growth. When we varied the ATP production stoichiometry to 10, 20, or 30 ATP per amino acid, the optimal catabolic flux fractions became 0.38, 0.22, or 0.15 in this model. In all cases, the optimal growth rate determined by simulation strongly correlated with the optimal growth rate predicted by the objective function (*SI Appendix*, Fig. S4).

Altogether, our results show that our biosynthesis toy model can be used to validate the objective function of ATP balance for optimal growth. By using an SRN formulation, this type of model can be easily generalized to include any number of nodes and reactions.

### Autocatalytic Circuits for SRNs.

For a system to grow autonomously (e.g., free-living organisms), it must contain a reaction network that is autocatalytic. What makes a network autocatalytic? Theorists have addressed this fundamental question and identified design principles of autocatalysis using discrete symbolic models (32, 33). As shown below, by using SRNs we can bridge from symbolic models to continuous dynamical systems (described by differential equations), demonstrating that the existence of autocatalytic properties is a necessary condition for a positive long-term growth rate (λ>0).

Here, we refer to a reaction $\phi_{a}$ as maintained by node $x_{z}$, if $J_{a}\left(X\right)=0$ whenever $X_{z}=0$, and designate the maintenance set of $\phi_{a}$ to be the collection of nodes that maintain $\phi_{a}$, which we denote as $mt\left(\phi_{a}\right)$. Note that the maintenance set of the reaction $\phi_{a}$ only includes the components that directly affect the magnitude of the flux $J_{a}$. For example, in a biochemical network, the immediately upstream precursors, the enzymes and the coenzymes that are directly involved in the reaction are all essential for generating a positive flux. Hence, they all belong to the maintenance set of the given reaction.

A collection of reactions K is called an autocatalytic circuit if

$$\underset{\phi_{a}\in K}{\cup }mt\left(\phi_{a}\right)\subseteq \underset{\phi_{a}\in K}{\cup }dw\left(\phi_{a}\right)$$ [7]

where $dw\left(\phi_{a}\right)$ denotes the downstream nodes of reaction $\phi_{a}$ (Fig. 1*C*). Intuitively, an autocatalytic circuit is a collection of reactions capable of synthesizing their own maintenance set (see *SI Appendix*, Fig. S5 for an example). In biological systems, an autocatalytic circuit contains all central metabolic reactions and synthesis pathways for making essential macromolecules. We call a reaction network autocatalytic if it contains at least one autocatalytic circuit.

With the SRN formulation, we are able to establish a connection between the sign of λ (which is an analytical property given by Eq. **6**) and the presence of an autocatalytic circuit (which is an algebraic property given by Eq. **7**). Our main result is the following (see *SI Appendix*, *Supplementary Text* section 6 for proof):

Any SRN (x, ϕ, J) with λ>0 must have at least one autocatalytic circuit K⊆ϕ; furthermore, every node $x_{k}\in mt\left(K\right)$ has the same long-term growth rate λ.

The above result provides the basic topological constraint: If an SRN does not have an autocatalytic circuit, it cannot exhibit positive long-term growth rate under any parameter set and any initial condition. This topological constraint can be used to identify autocatalytic circuits and to rule out nongrowing topologies from thousands of random networks (see *SI Appendix*, Fig. S6 for examples of random reaction networks with a power-law connection probability).

## Discussion

Long-term growth is an essential property of living systems. How this remarkable property is achieved and maintained is one of the most fundamental questions in biology. In this study, we identify an important class of biological reaction networks, SRNs, whose long-term growth properties can be studied using powerful ergodic theory tools. With these tools, we mathematically demonstrate two basic principles of exponentially growing systems: scalability of the underlying flux functions and ergodicity of the rescaled system. Our mathematical framework explains how various growth dynamics (balanced growth, oscillatory or nonperiodic) driven by complex, nonlinear flux functions can converge to long-term exponential growth, which is prevalent in biological systems.

Our theory has a number of practical implications as it provides a rigorous mathematical foundation for modeling and probing natural and synthetic reaction networks with distinct advantages over existing methodologies. First, our approach does not a priori assume that a long-term growth rate exists; instead, this is a mathematically derived consequence of scalability and ergodicity. Second, as current methods are limited to the case of balanced growth, our theory considerably expands the type of systems that can be analyzed, including metabolic cycles (13, 14, 21, 23) and chaotic oscillations in communities (7). Third, once an SRN is constructed, there is no requirement for the dynamics of the system, $\vec{Y}\left(t\right)$, to be constrained by objective functions or phenomenological laws. In fact, with our approach, objective functions or phenomenological laws can be inferred or validated through large-scale simulation across the parameter space (Fig. 5 and *SI Appendix*, Fig. S4). Fourth, biological processes are stochastic in nature and thereby inherently noisy, yet noise is often neglected in current growth models. The ergodic theory formulation enables generalization from ordinary differential equations to stochastic differential equations and allows rigorous consideration of how noise impacts long-term growth.

From a technical standpoint, our study bridges two fields that seldom interact: ergodic theory and growth modeling. The key concept underlying this connection is the scalability of multivariate functions in reaction networks. This allows one to leverage a powerful branch of mathematics in the study of biological growth processes. Interestingly, in the field of ergodic theory, noise has emerged as an important factor to consider, as its averaging effect tends to produce simpler, more tractable dynamics, while at the same time capturing the system’s essential features (34–36). Consistent with this observation, we find that noise can enable SRNs to achieve ergodicity and thus helps systems attain a robust long-term growth rate (*SI Appendix*, *Supplementary Text* and Fig. S3). This highlights an unappreciated role for noise in biology.

Living systems (e.g., cells) are highly complex in their reaction network structure. Hence, realistic models of such systems can include hundreds to thousands of components (nodes) and reactions (fluxes) (37). Simulating this level of complexity is computationally challenging, largely because of open problems regarding 1) the stability of models, 2) the robustness of results to the choice of parameters and initial conditions, and 3) the behavior of the dynamics over long timescales. In our framework, an SRN regardless of its complexity can be projected into a bounded simplex space while preserving all of the key dynamical information. This results in well-behaved topological and analytical properties (simply connected space, straightforward parameterization, etc.), which will help address the aforementioned open problems.

Our mathematical framework is applicable for both exponential growth and decay. This allows one to study when a system transitions from growth to decay (or vice versa), which can be critical in the context of ecosystem management and economic development (38, 39). For a system to grow and expand exponentially, it must achieve λ>0. Under the SRN framework, we prove that a sufficient condition for achieving λ>0 is the existence of an autocatalytic circuit. Autocatalysis has been extensively studied, primarily using symbolic models (32, 33). Continuous dynamical models have also been reported (e.g., refs. 40–42). However, they are usually restricted to linear networks while the nonlinear network models are specialized for particular systems because of the absence of a theoretical framework. In contrast, our theory provides a generalizable and systematic way to connect symbolic and continuous models to analyze long-term growth rates from any SRN. We envision that our mathematical framework will facilitate future studies in subjects as diverse as network design and evolution, biochemical modeling of the cell, ecosystem growth and sustainability, and economic business cycles.

## Materials and Methods

Numerical solutions of differential equations were integrated using MATLAB (MathWorks) or Mathematica (Wolfram). The bifurcation diagrams and phase plots were generated by customized scripts in MATLAB. See *SI Appendix* for the detailed description of models and parameters used in the simulations. The theoretical derivation is described in *SI Appendix*, *Supplementary Text*.

## Supplementary Material

Supplementary File

## Data Availability

Simulation code used to generate the results shown in this study can be found at https://github.com/JacobsWagnerLab/.

## References

[1] Raa T. T., Economics of Input-Output Analysis, (Cambridge University Press, 2005).

[2] Kussell E., Leibler S., Phenotypic diversity, population growth, and information in fluctuating environments. Science 309, 2075–2078 (2005). 16123265 10.1126/science.1114383

[3] Barenholz U.., Design principles of autocatalytic cycles constrain enzyme kinetics and force low substrate saturation at flux branch points. eLife 6, e20667 (2017). 28169831 10.7554/eLife.20667 PMC5333975

[4] Posfai A., Taillefumier T., Wingreen N. S., Metabolic trade-offs promote diversity in a model ecosystem. Phys. Rev. Lett. 118, 028103 (2017). 28128613 10.1103/PhysRevLett.118.028103 PMC5743855

[5] Agazzi A., Dembo A., Eckmann J.-P., On the geometry of chemical reaction networks: Lyapunov function and large deviation. J. Stat. Phys. 172, 321–352 (2018).

[6] Blasius B., Rudolf L., Weithoff G., Gaedke U., Fussmann G. F., Long-term cyclic persistence in an experimental predator-prey system. Nature 577, 226–230 (2019). 31853064 10.1038/s41586-019-1857-0

[7] Becks L., Hilker F. M., Malchow H., Jürgens K., Arndt H., Experimental demonstration of chaos in a microbial food web. Nature 435, 1226–1229 (2005). 15988524 10.1038/nature03627

[8] Bordbar A., Monk J. M., King Z. A., Palsson B. O., Constraint-based models predict metabolic and associated cellular functions. Nat. Rev. Genet. 15, 107–120 (2014). 24430943 10.1038/nrg3643

[9] Scott M., Klumpp S., Mateescu E. M., Hwa T., Emergence of robust growth laws from optimal regulation of ribosome synthesis. Mol. Syst. Biol. 10, 747 (2014). 25149558 10.15252/msb.20145379 PMC4299513

[10] Marr A. G., Growth rate of Escherichia coli. Microbiol. Rev. 55, 316–333 (1991). 1886524 10.1128/mr.55.2.316-333.1991 PMC372817

[11] Kondo Y., Kaneko K., Growth states of catalytic reaction networks exhibiting energy metabolism. Phys. Rev. E Stat. Nonlin. Soft Matter Phys. 84, 011927 (2011). 21867233 10.1103/PhysRevE.84.011927

[12] Maitra A., Dill K. A., Bacterial growth laws reflect the evolutionary importance of energy efficiency. Proc. Natl. Acad. Sci. U.S.A. 112, 406–411 (2015). 25548180 10.1073/pnas.1421138111 PMC4299221

[13] Ahn E., Kumar P., Mukha D., Tzur A., Shlomi T., Temporal fluxomics reveals oscillations in TCA cycle flux throughout the mammalian cell cycle. Mol. Syst. Biol. 13, 953 (2017). 29109155 10.15252/msb.20177763 PMC5731346

[14] Papagiannakis A., Niebel B., Wit E. C., Heinemann M., Autonomous metabolic oscillations robustly gate the early and late cell cycle. Mol. Cell 65, 285–295 (2017). 27989441 10.1016/j.molcel.2016.11.018

[15] Tu B. P.., Cyclic changes in metabolic state during the life of a yeast cell. Proc. Natl. Acad. Sci. U.S.A. 104, 16886–16891 (2007). 17940006 10.1073/pnas.0708365104 PMC2040445

[16] Godin M.., Using buoyant mass to measure the growth of single cells. Nat. Methods 7, 387–390 (2010). 20383132 10.1038/nmeth.1452 PMC2862099

[17] Moore C. C., Ergodic theorem, ergodic theory, and statistical mechanics. Proc. Natl. Acad. Sci. U.S.A. 112, 1907–1911 (2015). 25691697 10.1073/pnas.1421798112 PMC4343160

[18] Kunita H., Stochastic Flows and Stochastic Differential Equations, (Cambridge University Press, 1997).

[19] Serdukova L., Zheng Y., Duan J., Kurths J., Stochastic basins of attraction for metastable states. Chaos 26, 073117 (2016). 27475077 10.1063/1.4959146

[20] Jack R., Ergodicity and large deviations in physical systems with stochastic dynamics. Eur. Phys. J. B 93, 74 (2020).

[21] Krishnaiah S. Y.., Clock regulation of metabolites reveals coupling between transcription and metabolism. Cell Metab. 25, 961–974.e4 (2017). 28380384 10.1016/j.cmet.2017.03.019 PMC5479132

[22] Wang J. D., Levin P. A., Metabolism, cell growth and the bacterial cell cycle. Nat. Rev. Microbiol. 7, 822–827 (2009). 19806155 10.1038/nrmicro2202 PMC2887316

[23] Gan S., O’Shea E. K., An unstable singularity underlies stochastic phasing of the circadian clock in individual cyanobacterial cells. Mol Cell. 67, 659–672.e12 (2017). 28803778 10.1016/j.molcel.2017.07.015

[24] Ferrezuelo F.., The critical size is set at a single-cell level by growth rate to attain homeostasis and adaptation. Nat. Commun. 3, 1012 (2012). 22910358 10.1038/ncomms2015

[25] May R. M., Leonard W. J., Nonlinear aspects of competition between three species. SIAM J. Appl. Math. 29, 243 (1975).

[26] Farmer R. E. A., The evolution of endogenous business cycles. Macroecon. Dyn. 20, 544–557 (2016).

[27] Elowitz M. B., Leibler S., A synthetic oscillatory network of transcriptional regulators. Nature 403, 335–338 (2000). 10659856 10.1038/35002125

[28] Hofbauer J., Sigmund K., The Theory of Evolution and Dynamical Systems: Mathematical Aspects of Selection, (London Mathematical Society Student Texts, Cambridge University Press, 1988).

[29] Russell J. B., Cook G. M., Energetics of bacterial growth: Balance of anabolic and catabolic reactions. Microbiol. Rev. 59, 48–62 (1995). 7708012 10.1128/mr.59.1.48-62.1995 PMC239354

[30] Flamholz A., Noor E., Bar-Even A., Liebermeister W., Milo R., Glycolytic strategy as a tradeoff between energy yield and protein cost. Proc. Natl. Acad. Sci. U.S.A. 110, 10039–10044 (2013). 23630264 10.1073/pnas.1215283110 PMC3683749

[31] Bender D. A., The metabolism of “surplus” amino acids. Br. J. Nutr. 108 (suppl. 2), S113–S121 (2012). 23107522 10.1017/S0007114512002292

[32] Kauffman S. A., Autocatalytic sets of proteins. J. Theor. Biol. 119, 1–24 (1986). 3713221 10.1016/s0022-5193(86)80047-9

[33] Hordijk W., Steel M., Detecting autocatalytic, self-sustaining sets in chemical reaction systems. J. Theor. Biol. 227, 451–461 (2004). 15038982 10.1016/j.jtbi.2003.11.020

[34] Baxendale P. H., Stability and equilibrium properties of stochastic flows of diffeomorphisms. Progr. Probab. 27 (1992).

[35] Young L. S., Comparing chaotic and random dynamical systems. J. Math. Phys. 60, 052701 (2019).

[36] Arnold L., Random Dynamical Systems, (Springer, ed. 2, 2003).

[37] Karr J. R.., A whole-cell computational model predicts phenotype from genotype. Cell 150, 389–401 (2012). 22817898 10.1016/j.cell.2012.05.044 PMC3413483

[38] Tamura R., From decay to growth: A demographic transition to economic growth. J. Econ. Dyn. Control 20, 1237–1261 (1996).

[39] Pinsky M. L., Byler D., Fishing, fast growth and climate variability increase the risk of collapse. Proc. Biol. Sci. 282, 20151053 (2015). 26246548 10.1098/rspb.2015.1053 PMC4632620

[40] Hinshelwood C. N., On the chemical kinetics of autosynthetic systems. J. Chem. Soc., 745–755 (1952).

[41] Eigen M., Schuster P., The hypercycle. A principle of natural self-organization. Part A: Emergence of the hypercycle. Naturwissenschaften 64, 541–565 (1977). 593400 10.1007/BF00450633

[42] Stadler B. M. R., Stadler P. F., Small autocatalytic reaction networks. 3. Monotone growth functions. Bull. Math. Biol. 53, 469–485 (1991).

