# Supplementary material for: Origin of exponential growth in nonlinear reaction networks
Source: Proc Natl Acad Sci U S A. 2020 Oct 22;117(45):27795–804. doi: 10.1073/pnas.2013061117 (PMC7668091; doi:10.1073/pnas.2013061117)
Supplement: Supplementary File [file pnas.2013061117.sapp.pdf]

PNAS
www.pnas.org

Supplementary Information for

Origin of exponential growth in nonlinear reaction networks

Wei-Hsiang Lin*, Edo Kussell, Lai-Sang Young and Christine Jacobs-Wagner*

Correspondence: jacobs-wagner@stanford.edu, whl243@stanford.edu

This PDF file includes:

Supplementary text
Figures S1 to S6
Tables S1 to S4
SI References

Table of contents

Page

Supplementary text: Mathematical theory of scalable reaction networks 3

0. Introduction 3

1. Flux network (x, ϕ, J) and long-term growth rate 3

2. Scalable reaction networks 6

3. Growth rates in scalable reaction networks with noise 15

4. An exponentially growing system with chaotic dynamics 18

5. Asymptotically scalable reaction networks 20

6. Autocatalytic property of scalable reaction networks 22

7. Appendix 26

Methods: Simulation and analysis of scalable reaction networks 30

1. Numerical solution of ordinary differential equations 30

2. Calculation of Poincare section and bifurcation analysis 30

3. Calculation of the largest Lyapunov exponent 30

4. Mathematical models of scalable reaction networks 32

(i) Autocatalytic single-repressilator model 32

(ii) Autocatalytic double-repressilator model 32

(iii) Ecosystem model of three competing and cross-feeding species 33

(iv) Autocatalytic biosynthesis model 39

(a) Quasi-steady-state formula for composite Michaelis-Menten equations 39

(b) Nodes, reactions and flux functions of the autocatalytic biosynthesis model 41

(c) Parameter range of autocatalytic biosynthesis model 42

5. Analysis of random networks and autocatalytic circuits 43

(i) Generation of random networks 43

(ii) Identification of autocatalytic circuits in random networks 44

(iii) Calculation of autocatalytic probability for random networks 44

Supplementary Figures 46

Fig. S1: Transformation of a scalable reaction network 46

Fig. S2: Modified May-Leonard system 47

Fig. S3: Characterization of the long-term behavior of the double-repressilator model 48

Fig. S4: Growth rate optimization under different ATP production stoichiometries 50

Fig. S5: Example of an autocatalytic circuit in a scalable reaction network 52

Fig. S6: Phase diagram of P_{auto}, the probability of a random network to be autocatalytic 53

Supplementary Tables 54

Table S1: Common flux functions 54

Table S2: Reactions and flux functions in the autocatalytic biosynthesis model 55

Table S3: Parameters used in the autocatalytic biosynthesis model 57

Table S4: Steady-state Y^* of the standard parameter set 58

References 58

Supplementary text:

Mathematical theory of scalable reaction networks

0. Introduction

A flux network is a fundamental structure that describes the interconversion of materials or resource in chemical, biological, and economic models. Although the transient dynamics for a general flux network can be extremely complicated, relatively simple long-term dynamics can emerge over a large timescale. For example, many biochemical reactions are complex nonlinear functions that depend on substrate and enzyme concentrations, yet the entire cell can follow a simple exponential growth at a large time scale.

Our goal is to characterize a mathematical class of flux networks that give rise to exponential dynamics in the long term. While a system evolves and increases its size, the relative proportion of each node also evolves. Since a growing process is not stationary, acquiring its long-term statistical averaging is generally not possible. Here, we explored the class of *scalable functions* (described in section 2) for constructing dynamical systems. The proportional scaling property allows us to decouple the *n*-dimensional dynamics into 1-dimensional "bulk dynamics" and (*n-1)*-dimensional "relative proportion dynamics". The latter dynamics is confined in the (*n-1)*-dimensional unit simplex, which enables us to apply ergodic theory for long-term statistical averaging.

In section 1, we introduce the basic mathematical structure for flux networks and the concept of long-term growth rate. In section 2, we introduce *scalable reaction networks* and present the main result (Theorem 2.3). This result guarantees, via the use of ergodic theory, converged long-term growth rates for many (though not all) initial conditions of scalable networks. Examples are discussed. In section 3, we consider the addition of scalable noise and show that the presence of such noise, together with a regenerative condition on the networks, guarantees well-defined long-term growth rates for all initial conditions. Along with the theory, we investigate concrete examples to illustrate non-steady-state growth modes and rich behaviors (including chaotic dynamics) for growing reaction networks. In section 5, we generalize the class to *asymptotic scalable networks*, which replaces the stringent "proportional scaling" condition to a more relaxed "asymptotic scaling". In section 6, we study the necessary structural motifs for the network to have a positive long-term growth rate.

1. Flux network $(\boldsymbol{x}, \boldsymbol{\phi}, \boldsymbol{J})$ and long-term growth rate

As in thermodynamics formulations, we consider a *system* that is connected to an *environment*. The environment (denoted as E) acts as a reservoir and provides unlimited supply and removal of materials. The system is composed by multiple nodes $x_1, \dots, x_n$ and interconversion reactions $\phi_1, \dots, \phi_m$. A reaction can happen between the environment and system nodes, as well as among multiple system nodes. In general, a reaction ϕ_a can be represented by

$$\phi_a\colon\ p_0E + p_1x_1 + \cdots + p_nx_n \ \rightarrow\ q_0E + q_1x_1 + \cdots + q_nx_n \qquad (1)$$

where p_k and q_k ($k = 0, \ldots, n$) represent the *stoichiometry coefficients* in this conversion. Specifically, whenever the reaction happens, a proportional amount of $E, x_1, \ldots, x_n$ is converted into another proportional amount of $E, x_1, \ldots, x_n$, where both proportions follow the relative ratio of p_j and q_j. It is clear that if $p_k > q_k$, the reaction ϕ_a consumes x_k and decreases the amount of material on node x_k.

The above description is simplified by classifying nodes that are increased, decreased, or unchanged during the conversion. If we denote $s_k \equiv p_k - q_k$ and environment $E \equiv x_0$, then the reaction ϕ_a can be rewritten as

$$\phi_a\colon \sum_{\{s_k<0\}} |s_k|\, x_k \rightarrow \sum_{\{s_k>0\}} |s_k|\, x_k \qquad (2)$$

for $k = 0,1,..,n$. Nodes x_k with $s_k < 0$ (or $s_k > 0$) are referred to be at the *upstream* (or *downstream*) of the reaction ϕ_a. Conceptually, each conversion reaction can be regarded as an "action" that decreases the amount of its upstream nodes and increases the amount of its downstream nodes. This can be visualized as the *network diagram*:

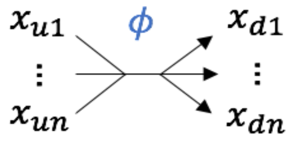

where $\{x_u\}$ and $\{x_d\}$ are the upstream and downstream nodes of the reaction ϕ, respectively. Note that the network diagram is more complex than the directed graph — it allows multiple upstream/downstream nodes.

A flux network can usually have multiple reactions $\{\phi_1, \ldots, \phi_m\}$. The net stoichiometry coefficient of reaction a on node k is denoted by S_{ka}. We define the n-by-m *stoichiometry matrix* S by $[S]_{ka} = S_{ka}$. This way, each column of S represents the stoichiometry coefficient of one reaction. Here, the stoichiometry coefficient of environment (x_0) is not included in S, since we assume unlimited supply/removal of materials from the environment.

Example: Consider the following flux network with $n = 4$ nodes and $m = 3$ reactions:

$$\begin{aligned} &\phi_1\colon E \ \rightarrow\ x_1 \\ &\phi_2\colon x_1 \ \rightarrow\ x_2 + x_3 \\ &\phi_3\colon x_2 + 2x_3 \ \rightarrow\ E + x_4 \end{aligned} \qquad (3)$$

The stoichiometry matrix S and the network diagram are below:

$$S = \begin{bmatrix} 1 & -1 & 0 \\ 0 & +1 & -1 \\ 0 & +1 & -2 \\ 0 & 0 & 1 \end{bmatrix}$$

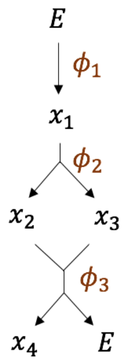

Above, we used the stoichiometry matrix S_{ka} to describe the topology of the reaction network. Each reaction ϕ_a is further associated with a flux function $J_a(X)$. The flux function can be linear such as $J_a(X) = X_1 + X_2$ or nonlinear such as $J_a(X) = e^{c(X_1/X_2)}$. Below, $X_1, \ldots X_n$ denote the *absolute amount* of material on nodes $x_1, \ldots, x_n$, and $J_1, \ldots, J_m$ denote the $flux$ of conversion reactions $\phi_1, .., \phi_m$.

Remark on notation: The x_k and ϕ_a are algebraic terms: x_k are node labels and ϕ_a are stoichiometry column vectors. They are used to describe the *network structure*. The X_k and $J_a(X)$ are multivariate nonnegative functions associated with x_k and ϕ_a, respectively. They are used to model the *network dynamics*.

In a small time interval Δt, the reaction ϕ_a causes X_k to change by an amount of $S_{ka} J_a \Delta t$, where S_{ka} is the net stoichiometry coefficient of flux a on node k. Under a continuous time limit, the deterministic dynamics of a flux network follows a differential equation. Given a node x_k, the rate of change of X_k is equal to the sum of all fluxes weighted by their stoichiometry coefficients on node x_k:

$$\frac{dX_k(t)}{dt} = \sum_{a=1}^{m} S_{ka} J_a \tag{4}$$

The flux magnitude J_a is usually a nonlinear function that depends on the amount or relative amount of materials in system nodes. In general, we write $J_a(X)$ where $X = (X_1, \ldots, X_n)^T$ is a column vector. For the general nonlinear function $J_a(X)$, there is no easy way to explicitly solve the equation. Our approach is to constrain the function form of $J_a(X)$ and study the long-term dynamics of the system.

Let $N \equiv X_1 + \ldots + X_n$ denote the total amount of material in the system, or the *system size*. We are interested in flux networks that have asymptotic exponential growth of $N(t)$. We define long-term growth rate λ by the following limit (if it exists):

$$\lambda \equiv \lim_{t \to \infty} \frac{1}{t} \log N(t) \tag{5}$$

Note that for an arbitrary flux network, this limit may not exist. The asymptotic behavior of $\frac{1}{t} \log N(t)$ can be different, such as (1) $N(t)$ becomes negative in the long term, and hence λ is

not well-defined, (2) $N(t)$ diverges at a finite time t_c and hence λ is not well-defined, and (3) $\frac{1}{t}\log N(t)$ diverges to infinity as $t \to \infty$, and (4) $\frac{1}{t}\log N(t)$ oscillates indefinitely and does not converge to a real number as $t \to \infty$. Therefore, it is clear that constructing flux networks with arbitrary flux functions may not give a well-defined long-term growth rate.

The long-term growth rate λ can be positive, negative or zero, which correspond to exponential growth, exponential decay, or other sub-exponential dynamics, respectively. The value of λ may also depend on the initial condition $X(0)$ of the system. Each trajectory can have a different λ. We explicitly denote this by $\lambda_{X(0),}$ if necessary.

Exponentially growing systems are often studied by starting with a system of equations $\frac{dX_k}{dt} = F_k(X)$ and assuming *a priori* that there exist (i) a long-term growth rate λ and (ii) a *balanced growth vector* X^* such that for $k = 1, \ldots, n$

$$F_k(X^*) = \lambda X_k^* \tag{6}$$

If the above equation has a solution (λ, X^*), then on the half-line $\{X = cX^*, c > 0\}$ the system trajectory follows a *balanced growth solution* $X(t) \sim X^* e^{\lambda t}$. In biological models, the λ mentioned above is regarded as the *dilution rate* for the cells or the chemostat. Still, it is unclear how to find the proper $F_k(X)$ such that the above solution exists. To our knowledge, there are few empirical rules for constructing nonlinear, exponential growing networks. Moreover, the above method only identifies balanced growth solutions and no other types of growth dynamics, such as oscillations, which occur in many biological systems. In the following section, we focus on a class of flux functions $J_a(X)$ for which the convergence of λ can be rigorously analyzed.

2. Scalable reaction networks

In this manuscript, we denote $Q^n\colon \{X \in \mathbb{R}^n \mid X_k \geq 0\}$ as the non-negative quadrant and $Q^{n+}\colon \{X \in \mathbb{R}^n \mid X_k > 0\}$ as the positive quadrant. For all discussions, we will assume that the system $X(t)$ has the initial condition $X(0) \in Q^n$, since the amount of starting material (e.g., metabolites) should be nonnegative in living systems. Recall that a node x_k is *upstream* of flux ϕ_a, if the stoichiometric coefficient $S_{ka} < 0$.

Definition 2.1: A flux function $J_a(X)\colon Q^n \to \mathbb{R}$ is *scalable* if it satisfies the following conditions:

(C1) (*differentiability*): $J_a(X)$ is positive in Q^{n+} and continuously differentiable in $Q^n \setminus \{0\}$.

(C2) (*upstream limited*): If node x_k is upstream of ϕ_a, then $J_a(X) = 0$ whenever $X_k = 0$.

(C3) (*proportional scaling*): $J_a(cX) = cJ_a(X)$ for all scalar $c > 0$ and all $X \in Q^n$. ■

Examples of scalable flux functions: Below, x_j represents an upstream node while x_p and x_z denote other nodes in the network, as exemplified here:

1. rX_j, with $r > 0$.
2. $\frac{rX_jX_p}{a_jX_j+a_kX_p+KN}$, with $a_j, a_k \geq 0, r, K > 0$.
3. $(1 - e^{X_j/N})X_z$
4. $[a + b\,sin(X_p/N)]X_j$, with $a > b > 0$.

Flux networks constructed by scalable functions are called scalable reaction networks (SRNs). SRNs have the nice property that the vector field satisfies the scaling property $F_k(cX) = cF_k(X)$. Therefore, once we specify the vector field on the $n-1$-dimensional unit simplex $\Delta^{n-1} \equiv \{X \in Q^n | X_1 + \ldots + X_n = 1\}$, the entire vector field can be obtained from the scaling relation. Furthermore, as we will show below, this scaling property is closely related to the asymptotic exponential growth of the system.

To analyze the scalable network, we change variable from the X-coordinate to (Y, N)-coordinate where $N = X_1 + \ldots + X_n$ and $Y_k = X_k/N$ for $k = 1, \ldots, n$ (see Fig. S1). The vector $Y = (Y_1, \ldots, Y_n)^T$ is the *relative fraction* of each node in the system. In general, the differential equation $\frac{dX_k}{dt} = F_k(X)$ can be transformed to (Y, N)-coordinates as

$$\frac{dY_k(t)}{dt} = \frac{F_k(NY)}{N} - \left(\frac{1}{N}\frac{dN}{dt}\right) Y_k$$
$$\frac{dN(t)}{dt} = \sum_{k=1}^{n} F_k\,(NY) \tag{7}$$

Next, we introduce the *instantaneous growth rate* of the system by $\mu = \frac{1}{N}\frac{dN}{dt}$. From the above equation, we have $\mu = \frac{1}{N}\sum_{k=1}^{n} F_k\,(NY)$. Now, we assume that all fluxes in the network are scalable. By the scaling property (C3), we have $F_k(NY) = NF_k(Y)$ for all $k = 1, \ldots, n$, and the above equation can be reduced to

$$\frac{dY_k(t)}{dt} = F_k(Y) - \mu(Y)\,Y_k$$
$$\frac{dN(t)}{dt} = N\mu(Y) \tag{8}$$
$$\mu(Y) = \sum_{k=1}^{n} F_k\,(Y)$$

Note that the instantaneous growth rate μ now becomes a function of Y only and is independent of N. The dynamics of $\frac{dY_k}{dt}$ become a function of Y only and are independent of N.

Since μ is a function of Y, the dynamics of $\mu(t)$ is determined by the dynamics of $Y(t)$. For scalable fluxes, the trajectory $Y(t)$ is confined in the $n-1$-dimensional unit simplex space $\Delta^{n-1} \equiv \{X \in Q^n | X_1 + \ldots + X_n = 1\}$. To see this, note that the upstream-limited condition (C2) guarantees that whenever the upstream node of a flux is zero, the flux itself is also zero. Hence,

the non-negative quadrant Q^n is forward invariant for $\frac{dX_k}{dt} = F_k(X)$. This implies that the unit simplex Δ^{n-1} is forward-invariant for $\frac{dY_k}{dt} = F_k(Y) - \mu(Y)Y_k$.

Now, we can express the long-term growth rate as the *time average* of μ:

$$\lambda \equiv \lim_{t\to\infty} \frac{1}{t} \log N(t) = \lim_{t\to\infty} \frac{1}{t} \int_0^t \frac{d \log N(s)}{ds} ds = \lim_{t\to\infty} \frac{1}{t} \int_0^t \mu\,(s) ds \tag{9}$$

Our next step is to replace the time average by the *space average* on the space Δ^{n-1}. To proceed, we need to introduce some concepts about ergodic theory. Below, we adopt the notation $F(X) = (F_1(X), \ldots F_n(X))^T$ as a vector function and denote $Y = (Y_1, \ldots, Y_n)^T$ as a vector on Δ^{n-1}. We denote $\mathcal{B}(S)$ as the Borel sets of S, and write a probability measure $\alpha(dY)$ as $d\alpha$.

Definition 2.2: Given a dynamical system $Y'(t) = G(Y)$ on $S \subseteq \mathbb{R}^n$, a Borel probability measure ω is called *invariant* if for every Borel set $A \subseteq S$, $\omega\{Y(t) \in A\} = \omega\{Y(0) \in A\}$ for all $t \in \mathbb{R}$. An invariant probability measure ω is called *ergodic* if the system has no invariant subset of intermediate ω-measure, i.e., there does not exist $A \subseteq S$ with $0 < \omega(\mathrm{A}) < 1$ such that $Y(0) \in A$ implies $Y(t) \in A$ for all t. ■

We remark that every continuous dynamical system on a compact space admits at least one ergodic measure [Ref(43), Thm 1.8.1]. Given a dynamical system $Y'(t) = G(Y)$ on $S \subseteq \mathbb{R}^n$ and an ergodic measure ω, the ergodic theorem asserts that time averages are equal to space averages, as described below.

Birkhoff Ergodic Theorem [Ref(43), Thm 1.2.1]. Let $Y(t)$ and ω be as above. Then for ω-almost every $Y(0) \in S$, we have

$$\lim_{t\to\infty} \frac{1}{t} \int_0^t f\big(Y(s)\big)\, ds = \int_S f\,(Y)\, d\omega \tag{10}$$

for every continuous function $f(Y): S \to \mathbb{R}$. ■

A point $Y(0) \in S$ is called *ω–regular*, or regular with respect to ω, if it satisfies Eq. (10) for every continuous function $f(Y)$. We say $Y(0)$ is regular if it is regular with respect to some ergodic measure. The following result is a direct consequence of Birkhoff Ergodic Theorem with $f(Y) = \mu(Y)$:

<u>Theorem 2.3</u>: Given a scalable reaction network, the long-term dynamics of $Y(t)$ is governed by

$$\frac{dY}{dt} = F(Y) - \mu(Y)Y \tag{11}$$

and is confined in Δ^{n-1}. Furthermore, if $Y(0)$ is regular with respect to an ergodic probability measure ω, then the long-term growth rate converges to

$$\lambda = \int_{\Delta^{n-1}} \mu\,(Y)\, d\omega \tag{12}$$

Note 1: Given an initial condition $Y(0)$ that is ω-regular, the ergodic probability measure ω can be constructed as follows. Let χ_B be the *characteristic function* such that:

$$\chi_B(Y) \begin{array}{l} = 1, \text{if } Y \in B \\ = 0, \text{if } Y \notin B \end{array} \tag{13}$$

where $B \subseteq \Delta^{n-1}$ is a Borel set. We define a family of probability measures $\{p_t\}_{t\geq 0}$ by

$$p_t(B) \equiv \frac{1}{t}\int_0^t \chi_B\left(Y(s)\right) ds \tag{14}$$

Intuitively, $p_t(B)$ is the fraction of time that $Y(t)$ stays within B during $[0, t]$. Then, as t tends to infinity, p_t converges in the weak* topology to ω, i.e., for every continuous function $f(Y): S \to \mathbb{R}$, the ergodic probability measure ω is the weak* limit such that

$$\lim_{t\to\infty} \int_S f\,(Y)\, dp_t = \int_S f\,(Y)\, d\omega \tag{15}$$

Conversely, for any initial condition $Y(0)$, if p_t converges to an ergodic measure ω, then $Y(0)$ is ω-regular. We note, however, that p_t may not converge for some $Y(0)$, and that the limit measure (even when it exists) need not be ergodic and may depend on $Y(0)$.

Note 2: If $\tilde{Y}(0)$ is another initial condition with $d(Y(t), \tilde{Y}(t)) \to 0$ as $t \to \infty$, then the growth rate at $\tilde{Y}(0)$ is also well-defined and equal to λ.

Note 3: It is possible that for some initial conditions (within a set of ω-measure zero) the growth rate does not converge to the value given by Eq. 12. Here, we analyze a deterministic example to illustrate this possibility.

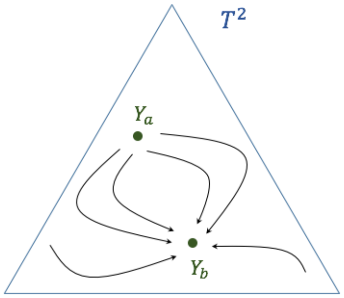

We consider a deterministic case on the simplex space T^{n-1} with *n* = 3 (see figure at right). The simplex space contains two fixed points: Y_a is unstable and Y_b is stable. For this system, we find two ergodic measures on T^2: the delta measures $\delta(Y - Y_a) \equiv \omega_a$ and $\delta(Y - Y_b) \equiv \omega_b$. Since Y_b is a stable attractor, all trajectories with the initial condition $Y(0) \neq Y_a$ converge asymptotically to the constant trajectory $Y(t) = Y_b$ and therefore have $\lambda = \mu(Y_b)$. The trajectory with $Y(0) = Y_a$ has $\lambda = \mu(Y_a)$. Thus, if $\mu(Y_a) \neq \mu(Y_b)$ then with respect to the ergodic measure ω_b we have that $\{Y_a\}$ is a set of measure zero for which λ does not converge to the value given by Eq. 12; and the same is true, respectively, for the ergodic measure ω_a and the set $T^2 \backslash \{Y_a\}$.

This discussion, together with Theorem 2.3, shows that for a scalable network, many initial conditions $X(0)$ are guaranteed to have well-defined long-term growth rates. This includes all $X(0)$ for which the rescaled initial conditions $Y(0)$ are regular.

We now consider a few illustrative examples:

(1) For a flux network with $Y(t)$ converging to a fixed point Y^*, the ergodic probability measure is a Dirac δ-measure supported on Y^*. The ergodic averaging for the long-term growth rate is reduced to $\lambda = \mu(Y^*)$. The long-term dynamics converges to balanced exponential growth on the half line $\{X = cY^*, c > 0\}$, with $X(t) \to Y^* e^{\mu(Y^*)t}$.

(2) For a flux network with $Y(t)$ converging to a limit cycle C, the ergodic probability measure is supported by the limit cycle. The long-term growth rate can be calculated by the contour integral $\lambda = \oint_C \mu\,(Y)\,d\omega$, and the dynamics converges to exponential growth with periodic functions $u_k(t)$ as prefactors, namely, $X_k(t) \to u_k(t)e^{\lambda t}$.

(3) There are attractors of $Y(t)$ that *do not* have an ergodic probability measure. One example is the heteroclinic cycle attractor, where the trajectory $Y(t)$ oscillates between M saddle points $\{Y_{s1}, \ldots, Y_{sM}\}$ with an increasing period. In this case, λ oscillates between the values $\{\mu(Y_{s1}), \ldots, \mu(Y_{sM})\}$ with an increasing period and does not converge to a real number.

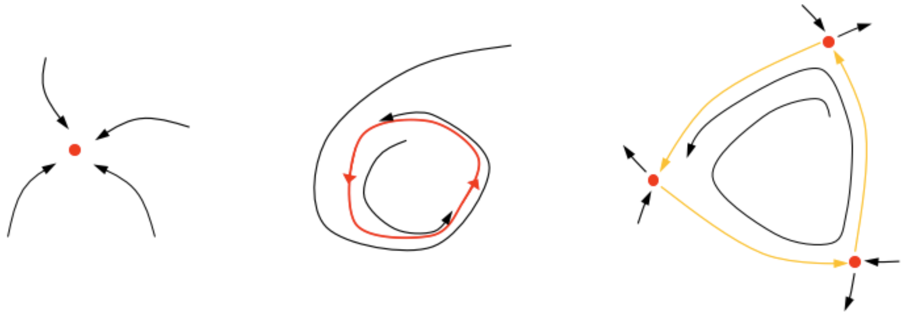

Different types of attractors and invariant measures. Red color indicates invariant sets in the system. Left: a stable fixed point attracting all nearby trajectories. Middle, a stable limit cycle attracting all nearby trajectories. Right, a heteroclinic cycle (union of red saddle points and yellow curves). The saddle points support ergodic measures. For the trajectory $Y(t)$ shown, the measures p_t in the construction above either do not converge or converge to a measure that is a linear combination of the ergodic measures supported on the three saddles. Such a measure is not ergodic. The initial condition $Y(0)$ is not regular and does not have a well-defined long-term growth rate.

As we have seen, balanced exponential growth belongs to one of the special (and simplest) types of exponentially growing networks. Additional growth modalities exist, which correspond to different types of attractors of $Y(t)$, as shown in the following two examples of SRNs.

Example 2.4: The autocatalytic repressilor network. In this example, we modify the repressilator model by Elowitz and Leibler [Ref(44)] to be an autocatalytic, scalable network. The system has four nodes, where x_1 represents the upstream metabolite and x_2, x_3, x_4 represent three mutually repressing components:

$$\begin{aligned}\frac{dX_1}{dt} &= J_1 - J_2 - J_3 - J_4\\ \frac{dX_2}{dt} &= J_2 - d_2X_2\\ \frac{dX_3}{dt} &= J_3 - d_3X_3\\ \frac{dX_4}{dt} &= J_4 - d_4X_4\end{aligned} \tag{16}$$

In the repressilator, the synthetic flux of nodes x_2, x_3, x_4 are repressed by node x_3, x_4, x_2, respectively. We implemented this by defining the synthetic flux function J_2, J_3, J_4 as decreasing functions of X_2, X_3, X_4. With Y_k denoting the fraction of X_k, the flux functions are: $J_1(X) = b_2X_2 + b_3X_3 + b_4X_4$, $J_2(X) = \frac{c_{syn}}{1+KY_3^\theta}X_1$, $J_3(X) = \frac{c_{syn}}{1+KY_4^\theta}X_1$, $J_4(X) = \frac{c_{syn}}{1+KY_2^\theta}X_1$.

The autocatalytic repressilator network can exhibit different growth modes, depending on the cooperativity coefficient θ (see Fig. 3A-E in main text). For $\theta = 1$, $Y(t)$ converges to a stable fixed point, while for $\theta = 3$, $Y(t)$ converges to a limit cycle, which corresponds to a periodic growth mode.

Example 2.5: Modified May-Leonard system. In this example, we study density-dependent growth of three species, which illustrates a case where $Y(t)$ is a heteroclinic cycle and $Y(t)$ is not regular with respect to any ergodic probability measure. The model is modified from the classical May-Leonard model [Ref(45)], with scalable flux functions as birth and death processes. Let x_1, x_2, x_3 denote the three species, the growth equation is

$$\begin{aligned}\frac{dX_1}{dt} &= X_1 \cdot r_1\left(a - \frac{c_1X_1}{N} - \frac{c_2X_2}{N} - \frac{c_3X_3}{N}\right)\\ \frac{dX_2}{dt} &= X_2 \cdot r_2\left(a - \frac{c_3X_1}{N} - \frac{c_1X_2}{N} - \frac{c_2X_3}{N}\right)\\ \frac{dX_3}{dt} &= X_3 \cdot r_3\left(a - \frac{c_2X_1}{N} - \frac{c_3X_2}{N} - \frac{c_1X_3}{N}\right)\end{aligned} \tag{17}$$

This system can be mapped to a network diagram by defining 12 scalable flux functions (see Fig. S2a), three for the birth process and nine for the death process. The death process is driven by a nonlinear flux function in the form of $(X_jX_k)/N$.

In our simulation, we find that the trajectory $Y(t)$ of the flux network exhibits similar behavior as in the original May-Leonard system. Consistent with the analytical results of the May-Leonard system, varying c_2 and c_3 changes the system behavior drastically (see Fig. S2):

(1) For $c_2 + c_3 < 2$ (Fig. S2B), the system has balanced growth and three species co-exist.

(2) For $c_2 > 1$ and $c_3 > 1$ (Fig. S2C), species X_1 is dominant and grows exponentially, while the other species X_2 and X_3 go extinct. The system still undergoes balanced growth, with fixed point $Y^*(t)$ = (1,0,0) on the boundary of Δ^2.

(3) For $c_2 + c_3 > 2$ and $c_2 < 1 < c_3$ (Fig. S2D), $Y(t)$ asymptotically converges to a heteroclinic cycle on the boundary of the system. The heteroclinic attractor $C \equiv \{Y \in \Delta^2 | Y_1 Y_2 Y_3 = 0\}$ is the omega-limit set of $Y(t)$, but $Y(t)$ is not regular with respect to any ergodic probability measure. In this case, each component $Y_k(t)$ oscillates between 0 and 1 while each period becomes exponentially longer. On the other hand, the overall system $X(t)$ grows unboundedly without the long-term growth rate λ converging to a real number (see Remark (3) in Proposition 2.4).

Definition 2.6: A *quasi-periodic trajectory* $Y(t)$ with *q frequencies* is a trajectory that is parametrized by $Y(t) = h(A(\theta_1 t, \ldots, \theta_q t))$, where h is a continuous function from torus T^q to S, and $A: T^q \to T^q$ is a continuous map between torus T^q with rationally independent frequencies $\{\theta_1, \ldots, \theta_q\}$ (i.e., $\sum_{k=1}^{q} c_k \theta_k = 0$ has no integer solution except $c_1 = \ldots = c_k = 0$).

Proposition 2.7: Consider an SRN with the initial condition $X(0) \in Q^n$. If the trajectory of $Y(t)$ converges to a fixed point, a periodic cycle, or an invariant torus with quasi-periodic dynamics, then $Y(0)$ is regular for some ergodic probability measure.

Proof: For $Y(t)$ converging to a fixed point Y^* the ergodic probability measure is $\delta(Y - Y^*)$. For the q-frequency torus (including $q = 1$ case for periodic trajectory), the trajectory is parametrized by equation $Y(t) = A\left(\theta_1 t, \ldots, \theta_q t\right)$, which follows

$$\frac{dA_k}{dt} = \theta_k t \tag{18}$$

with $k = 1, \ldots, q$. The flow $A(t)$ on the torus T^q is uniquely ergodic with respect to Lebesgue measure on T^q [Ref(43), Thm 3.1.2], Hence $A(t)$ has an ergodic probability measure on T^q. Since both h and $A(t)$ are continuous, this implies $Y(t)$ also has an ergodic probability measure on Δ^{n-1}. ■

Example 2.8: SRN-based models for chemostat-type systems

In this section, we study the relation between the SRN framework and chemostat-type equations. The general SRN framework assumes unlimited external resources, which conflicts with chemostat conditions in which an essential nutrient is in limited amount. However, for a chemostat equation, there exists an equivalent SRN model that gives rise to the same dynamics. Below, we use classical chemostat equations [Ref(46)] for demonstration.

Let $s(t)$ and $x(t)$ represent the concentrations of an upstream-limited nutrient and the microorganism in the chemostat culture vessel, respectively. The dynamics of $s(t)$ and $x(t)$ follows the chemostat equations:

$$\frac{ds}{dt} = D(s_{in} - s) - f(s)x$$

$$\frac{dx}{dt} = bf(s)x - Dx$$

where D is the dilution rate of the culture, s_{in} is the constant level of the external nutrient supply, b is the stoichiometry constant for the biomass yield, and $f(s) = f_{max}\frac{s}{K+s}$ is the Monod's function, which describes the dependence of the nutrient uptake rate (influx) on the extracellular nutrient level $s(t)$.

The above chemostat equations are not scalable (e.g. $f(s)$ does not satisfy $f(cs) = cf(s)$). However, since the equations are formulated in terms of concentrations $s(t)$ and $x(t)$, the system dynamics should be independent of the chemostat volume. Intuitively, scaling the chemostat volume to different sizes should yield identical dynamics of concentrations. Therefore, if we reformulate the equation in terms of the total number of molecules, the system should be an SRN.

To rigorously show this, we include the solvent molecule species w in the equations. Let S, X, and W be the total numbers of nutrient molecules, cells, and solvent molecules in the system, and let τ_S, τ_X, τ_W be their unit volumes. The <u>total culture volume</u> $V > 0$ is a time-independent positive constant and is given by:

$$V = S\tau_S + X\tau_X + W\tau_W = \text{constant}$$

This imposes a constraint among S, X, and W, and we have the relations $s = S/V$, $x = x/V$, and $w = W/V$. We refer to the quantities (s, x, w) as *intensive variables* and (S, X, W) as *extensive variables*, similar to thermodynamics definitions.

Since V is a constant, we have

$$\frac{dS}{dt} = V\frac{ds}{dt}$$

Note that the variable $\frac{ds}{dt}$, which is a function of intensive variables (s, x), is scale-invariant under scaling $(S, X, W) \rightarrow c(S, X, W)$. To see this, note that

$$s = \frac{S}{V} = \frac{S}{S\tau_S + X\tau_X + W\tau_W} = \frac{cS}{c(S\tau_S + X\tau_X + W\tau_W)}$$

$$x = \frac{X}{V} = \frac{X}{S\tau_S + X\tau_X + W\tau_W} = \frac{cX}{c(S\tau_S + X\tau_X + W\tau_W)}$$

Therefore, $S'(t) = V\, s'(t)$ is a scalable function. Similarly,

$$\frac{dX}{dt} = V\frac{dx}{dt}$$

The variable $\frac{dx}{dt}$, which is a function of intensive quantities (s, x), is also scale-invariant under scaling $(S, X, W) \rightarrow c(S, X, W)$. Therefore, $X'(t) = V\, x'(t)$ is a scalable function.

For the solvent variable $W(t)$, by the constraint of constant total volumes V, we have

$$\frac{dW}{dt} = \left(\frac{-\tau_S}{\tau_W}\right)\frac{dS}{dt} + \left(\frac{-\tau_X}{\tau_W}\right)\frac{dX}{dt}$$

Since both $S'(t), X'(t)$ are scalable, $W'(t)$ is also scalable. Therefore, the ODE of the new variables (S, X, W) is an SRN and is equivalent to the chemostat equations. The same mapping can be applied for studying more complex chemostat dynamics, including multi-species interactions (as shown in Fig. 4E and described in SI in the Method section).

Remark: For the above equivalent SRN model mentioned above, the long-term growth rate of the system (S, X, W) is zero. This is due to the constraint that the total volume $V = S\tau_S + X\tau_X + W\tau_W$ is a positive constant, and hence it is impossible to have $\lambda > 0$ (V diverges to infinity) or $\lambda < 0$ (V converges to 0).

The SRN framework provides additional insight into how biomass growth rate, $bf(s)$, is related to the dilution rate D. We define

$$\mu_X \equiv \frac{1}{X}\frac{dX}{dt} = \frac{1}{x}\frac{dx}{dt} = bf(S/V) - D$$

If the dilution rate is higher than the maximal biomass growth rate (i.e., $D > bf_{max}$), then $\mu_X < 0$ and all cells in the chemostat will be washed away. For cells to persist in the chemostat culture, the time average of μ_X must equal λ. For the equivalent SRN model, we have $\lambda = 0$ as mentioned above. Using the SRN formulation, we integrate μ_X with respect to an ergodic measure ω, which yields

$$0 = \lim_{T\to\infty}\frac{1}{T}\int_0^T \mu_X(t)\,dt = \lim_{T\to\infty}\frac{1}{T}\int_0^T [bf(S/V) - D]\,dt = \int [bf(S/V) - D]\,d\omega$$

and hence

$$D = \lim_{T\to\infty}\frac{1}{T}\int_0^T bf(S/V)\,dt = \int bf(S/V)\,d\omega \;.$$

In general, the constant dilution rate D is equal to the average biomass growth rate $bf(S/V)$ with respect to an ergodic measure ω. For balanced growth (as a special case), we have $D = bf(S^*/V^*)$ where (S^*, X^*, W^*) is the fixed point of the system. The same relation holds for more complex chemostat dynamics. For example, when a multi-species chemostat exhibits oscillatory behavior, the limit cycle has the property that the average biomass growth rate on the limit cycle is equal to the dilution rate. This also applies for dynamics which include a limit torus or chaos.

3. Growth rates in scalable reaction networks with noise

For scalable reaction networks, ergodic theory offers a conceptual understanding of long-term growth rates. Because the rescaled system takes values in a compact region, long-term growth rates are guaranteed to exist for many initial conditions, namely those that are regular with respect to some ergodic probability measure. Given a particular initial condition, however, growth properties are not always easy to determine, because not every initial condition is regular and more complicated systems often have multiple ergodic measures each with their own set of regular points and growth rates. This less-than-ideal state of affairs is improved greatly in the presence of random noise. Real-world systems are inherently noisy and mathematically stochastic terms are often added to model uncontrolled fluctuations in physical and biological systems. As we will show in this section, the averaging effect of random noise leads to a simpler and more tractable dynamical picture.

To investigate how flux noise affects the system's behavior, we generalize the ordinary differential equation $\frac{dX_k}{dt} = F_k(X)$ into a stochastic differential equation (SDE). As before, we let $Q^n\!: \{X \in \mathbb{R}^n \mid X_k \geq 0\}$, and $Q^{n+}\!: \{X \in \mathbb{R}^n \mid X_k > 0\}$.

Definition 3.1: A *scalable reaction network with scalable noise* is a systems SDE

$$dX_k = F_k(X)dt + H_k(X) \circ dB_k \tag{19}$$

for $k = 1, \ldots, n$. Let S denote the constant *stoichiometry matrix* and define the deterministic flow by $F_k(X) = \sum_a S_{ka} J_a(X)$. Every flux J_a needs to satisfy the following properties:

(C1) $J_a(X)$ is positive in Q^{n+} and continuously differentiable in $Q^n \setminus \{0\}$.
(C2) If the node x_k is one of the upstream nodes of ϕ_a, then $J_a(X) = 0$ whenever $X_k = 0$.
(C3) $J_a(cX) = cJ_a(X)$ for arbitrary scalar $c > 0$ for all $X \in Q^n$.

The noise function $H_k(X)$ satisfies:

(N1) $H_k(X) > 0$ on $\{X \in \mathbb{R}^n \mid X_k > 0\}$ and twice continuously differentiable in $Q^n \setminus \{0\}$.
(N2) $H_k(X) = 0$ whenever $X_k = 0$.
(N3) $H_k(cX) = cH_k(X)$ for arbitrary $c > 0$.

Here, dB_k represents the standard Brownian motion, with integral in Stratonovich-sense. ■

In the above SDE, the deterministic part of the equation, i.e., $F(X)$, has identical requirements as in scalable flux functions. We consider a noise function H_k which also scales with the system size. This is motivated by the following reasons:

(i) In nature, the environment is never truly static. For example, the environmental temperature has nonzero fluctuations and this globally affects all enzyme activities. Consider a flux $J = rX_k$ where the rate constant $r = r(T)$ is a function of temperature T. Fluctuations of the environmental temperature δT create noise $\delta r \approx r'(T)\delta T$ on the rate constant r. Hence, this creates noise on flux $\delta J \approx (\delta r)X_k = (\delta r/r)J$. This noise scales with the flux magnitude and therefore scales with system size.

(ii) Consider a system with finite space, for example, a bacterial culture in a turbidostat, the population is kept at a constant size C while the excess of growing bacteria is discarded by medium dilution. The dilution process usually creates a sampling noise $\delta C \sim \sqrt{C}$. In this case, the growth dynamics is equivalent to an exponentially growing population with N/C copies of subsystem with size C. If the exponential growing population has size N and a noise level $\frac{N}{C}\sqrt{C}$, then each subsystem has a fluctuation $\sqrt{C}$. In this case, the equivalent exponentially growing population has a noise level that scales with system size.

In addition to scalable noise, our formulation allows a system with $\lambda > 0$ to have other types of noise that grow slower than the system size. These "sub-scalable noises" will decay to zero as the system grows to infinity. Therefore, only the scalable noise term remains.

To establish the existence and uniqueness of solutions for all $t > 0$ for the SDE in Eq. (19), we convert our SDE from Stratonovich to the Ito formulation (see [Ref(47)]). Since $H(X)$ is a diagonal matrix, hence $(H)_{kl} = \delta_{kl}H_k$, this simplifies to

$$\begin{aligned} H_k^{(Ito)}(X) &= H_k(X) \\ F_k^{(Ito)}(X) &= F_k(X) + \frac{1}{2}\frac{\partial H_k(X)}{\partial X_k}H_k(X) := \; v_k(X) \end{aligned} \tag{20}$$

Existence and uniqueness are then checked by appealing to classical results (e.g. [Ref(47)], Theorem 5.6.1). Details are given in the Appendix.

As in the deterministic case, we now consider the rescaled SDE $Y(t)$ defined by $Y_k \equiv X_k/|X|$, $|X| = X_1 + \ldots + X_n$. We begin with a result asserting that starting from an initial condition where $X_k > 0$ for all k, i.e., all the substances in the network are present in positive amounts, no substance will be depleted in finite time.

Proposition 3.2: The trajectory $Y(t)$ of Eq. (19) is confined in the unit simplex Δ^{n-1}. For each component k, if $Y_k(0) > 0$, then $Y_k(t) > 0$ for all $t > 0$.

Proof: One way to prove this is to produce a Lyapunov function V on the interior of Δ^{n-1} with $V(Y) \to \infty$ as Y tends to $\partial\Delta^{n-1}$, the boundary of Δ^{n-1}. The function V is to have the property $\mathcal{A}^*V \leq CV$ for some fixed $C > 0$ where $\mathcal{A}^*$ is the diffusion operator of the SDE; it controls the speed with which solutions can approach the boundary. A complete proof is given in the Appendix. ■

The SDE (19) generates a time-homogeneous Markov process on Δ^{n-1}. For $Y_0 \in \Delta^{n-1}$ and $s > 0$, we use $P_s(Y_0, \cdot)$ to denote the *transition probability* starting from Y_0, that is, if $B \subseteq \Delta^{n-1}$ is a Borel set, then $P_s(Y_0, B)$ is the probability that $Y(s) \in B$ given that $Y(0) = Y_0$. A probability measure ω is said to be *stationary* if $\omega(B) = \int P_s(Y, B)\omega(dY)$ for all Borel sets B and all $s > 0$. A stationary probability measure ω is called *ergodic* if there is no subset $A \subset \Delta^{n-1}$ with $0 < \omega(A) < 1$ such that $P_s(Y, A) = 1$ for $Y \in A$ and $s > 0$. The Ergodic Theorem in section 2 applies in this setting as it does in the deterministic case.

Corollary 3.3 (*Dichotomy for ergodic measures*): Consider an SRN with noise as defined by Eq. (19), and let ω be an ergodic probability measure of the rescaled system. Then we have either $\omega(\partial\Delta^{n-1}) = 0$ or 1.

Proof: This follows immediately from Proposition 3.2, which says that the interior of Δ^{n-1} is an invariant set, so it must have measure 0 or 1 by the ergodicity of ω. ■

Below, we give a sufficient condition for the existence of long-term growth rates for noisy scalable networks. Following the notation in Eq. (20), we call a system *regenerative* if for every $k = 1, \ldots, n$, the kth component of the drift vector $v_k(Y) > 0$ whenever $Y_k = 0$. Mathematically, this means the drift term in the SDE points into the interior of the simplex Δ^{n-1} at all points on its boundary.

<u>Theorem 3.4</u>: Consider an SRN with noise as defined by Eq. (19) and assume that the system is regenerative. Then, there is a number λ with the property that starting from any initial condition $(0) \in \Delta^{n-1}$, the long-term growth rate converges almost surely to λ.

Proof: First, ergodic stationary measures exist because the process is confined to a compact region and the transition probabilities $P_s(Y,\cdot)$ vary continuously with Y. Let ω be an ergodic probability measure. By Corollary 3.3, $\omega(\partial\Delta^{n-1}) = 0$ or 1. The regenerativeness of the network implies $\omega(\partial\Delta^{n-1}) = 0$, because the positivity of v_k pushes all initial conditions in $\{Y_k = 0\}$ into the interior of Δ^{n-1} immediately. Next we observe that ω has a density, i.e., $\omega(dY) = \rho(Y)dY$ for some integrable function $\rho(Y)$. This is because $\omega = \int P_s(\mathrm{Y},\cdot)\omega(dY)$ and $P_s(\mathrm{Y},\cdot)$ has a density for all Y in the interior of Δ^{n-1} by condition (N1) above. Observe that $\rho(Y) > 0$ on all of Δ^{n-1} by standard properties of diffusions. The uniqueness of ω follows, as all ergodic probability measures have strictly positive densities and distinct ergodic measures are supported on disjoint sets. Finally, the Birkhoff Ergodic Theorem applied to the function $\mu(Y)$ gives the result that the long-term growth rate of every $Y(0) \in \Delta^{n-1}$ exists almost surely and is equal to $\lambda = \int \mu(Y)\omega(dY)$. ■

We remark that even though the invariant density $\rho(Y)$ is positive on all of Δ^{n-1}, it is in general much more concentrated in some regions of Δ^{n-1} than in other regions, that is to say, certain network configurations (in terms of proportions of the substances present) are much more prevalent even though there is a small theoretical probability of exploring other parts of the phase space. What makes the situation in Theorem 3.4 more tractable than the noise-free case is the uniqueness of ergodic measure and the fact that every initial condition is regular with respect to the unique ergodic measure.

The general case (of not-necessarily-regenerative networks): Not all realistic biological networks are regenerative. Not being regenerative means that there is no explicit mechanism in place for each substance to restore itself as it heads towards relative depletion. To be precise, relative depletion here means the rescaled amounts $Y_k(t)$ (but not necessarily the actual amounts $X_k(t)$) become arbitrarily small. Ergodic measures always exist for the same reasons as before, but unlike the regenerative case, there may exist ergodic measures supported on $\partial\Delta^{n-1}$. For example, if $v_k \equiv 0$ on $\{Y_k = 0\}$, then starting from $Y(0)$ with $Y_k(0) = 0$, almost surely $Y_k(t) = 0$ for all $t > 0$ (see condition (N2)), so there is a subsystem defined on the (n-2)-dimensional simplex $\Delta^{n-1} \cap \{Y_k = 0\}$ with its own ergodic measures.

In this general case, let us consider only initial conditions starting from the interior of Δ^{n-1}. There is the following dichotomy:

Case 1. Existence of an ergodic measure with a density $\rho(Y) > 0$ on all of Δ^{n-1}. In this case, long-term growth rates of all $Y(0)$ in the interior of Δ^{n-1} converge almost surely to $\lambda = \int \mu(Y)\rho(Y)dY$, as before.

Case 2. All ergodic measures are supported on $\partial\Delta^{n-1}$. In this case, starting from $Y(0)$, in the interior of Δ^{n-1}, one or more of the substances will come close to relative depletion (though none can deplete in finite time according to Proposition 3.2). More precisely, given any $Y(0)$, for any $\varepsilon > 0$ we have Prob$\{d(Y(t), \partial\Delta^{n-1}) > \varepsilon\} \to 0$ as $t \to \infty$. We summarize our findings below, leaving mathematical proofs to be published elsewhere.

Since trajectories spend nearly 100% of their time near $\partial\Delta^{n-1}$, their large-time behaviors are dictated by the ergodic measures supported on $\partial\Delta^{n-1}$. Now $\partial\Delta^{n-1}$ is a finite union of (n-2)-dimensional simplices and an analysis similar to that for Δ^{n-1} can be made for each of these simplices. After successively reducing dimension, we arrive at the conclusion that i) the system has at most a finite number of ergodic invariant measures, each supported on a lower dimensional simplex and ii) in this lower dimension, the system has a density with respect to the Lebesgue measure.

It may happen that $Y(t)$ converges almost surely to one of these ergodic measures ω, in which case its long-term growth rate is given by $\lambda = \int \mu(Y)\omega(dY)$. Another possibility is for $Y(t)$ to explore multiple ergodic measures one after another (in a way similar to the heteroclinic loop example in section 2). In this case, there may not be a single number that correctly describes the trajectory's long-term growth rate.

4. An exponentially growing system with chaotic dynamics

In the following example, we study a flux network converging to a nonperiodic trajectory and investigate the effect of noise on its long-term growth rate.

The double repressilator network. We construct a flux network with two repressilators repressing each other (see Fig. 2F and Fig. S3). The double repressilator network consists of seven nodes: x_1 represents the common precursor, $x_2, x_3,$ and x_4 form the first repressilator, and $x_5, x_6,$ and x_7 form the second one. In addition, the two repressilators are coupled by mutual repression, where nodes x_2 and x_5 repress the influxes of each other. The full differential equation is:

$$\frac{dX_1}{dt} = F_1(X) = J_1 - \sum_{k=2}^{7} J_k$$
$$\frac{dX_k}{dt} = F_k(X) = J_k - d_k X_k, \text{for } k = 2, \ldots, 7 \qquad (21)$$

$$
\begin{aligned}
&J_1(X) = \sum_{k=2}^{7} X_k \\
&J_2(X) = R_a(Y)\frac{cX_1}{1 + K_a Y_3^\theta}, \quad J_3(X) = \frac{cX_1}{1 + K_a Y_4^\theta}, \quad J_4(X) = \frac{cX_1}{1 + K_a Y_2^\theta} \\
&J_5(X) = R_b(Y)\frac{cX_1}{1 + K_b Y_6^\theta}, \quad J_6(X) = \frac{cX_1}{1 + K_b Y_7^\theta}, \quad J_7(X) = \frac{cX_1}{1 + K_b Y_5^\theta} \\
&R_a(Y) = \frac{1}{1 + M_a Y_5^4}, \quad R_b(Y) = \frac{1}{1 + M_b Y_2^4},
\end{aligned}
\tag{22}
$$

Note that this flux network satisfies the regenerative condition in Theorem 3.4. The parameters K_a and K_b are the repression strengths of repressilators $\{x_2, x_3, x_4\}$ and $\{x_5, x_6, x_7\}$, respectively. We define $\alpha \equiv K_a/K_b$ as the relative repression strength between the two repressilators. By varying α, the system exhibits complex behaviors, including periodic, quasi-periodic, and non-periodic trajectories of $Y(t)$. For different values of parameter α, the omega-limit set of the $Y(t)$ can have complex geometry such as a limit torus ($\alpha = 20$), a complicated limit cycle ($\alpha = 100$) and a strange attractor ($\alpha = 500$) (Fig. 2G).

To show how the attractor of $Y(t)$ transitions from a limit cycle to a limit torus to a more complex attractor, we project the attractor on the $Y_2 - Y_3$ plane and get a Poincare section at $\{Y_2 = 0.2\}$. For various α, we see that the system exhibits rich behavior, including period doubling and transition to chaos (Fig. 2G, insets). For the full bifurcation diagram, see Fig. S3A.

To quantitatively analyze the attractor, we numerically calculated the largest Lyapunov exponent (LLE) of the trajectories Fig. S3B). LLE quantifies how small perturbations propagate along the solution trajectory, and is a useful indicator for chaotic dynamics: positive LLE indicates strange attractor while LLE=0 indicates a limit torus, a limit cycle or a fixed point. We found that for α with values between 400-500, LLEs are significantly positive, which is consistent with what is qualitatively observed in the bifurcation diagram. There are also sporadic "chaotic islands" for $\alpha = 120$.

For comparison, we introduce a linear noise $H_k(X) = h_k X_k$ on the SDE. We find that the LLEs with and without noise (black versus light blue lines in Fig. S3B) are comparable, indicating that the chaotic behavior is robust under perturbation. Although it is difficult to show the convergence of λ in a nonperiodic, deterministic system, there is noise associated with fluxes in most realistic systems. By introducing small noise, we can infer whether the long-term behavior observed in the deterministic system is robust. By Theorem 3.4, λ converges under arbitrarily small h_k. We simulate long-term growth rate with $h_k = 0$ and $h_k = 0.1$ for various α (Fig. S3C). For non-chaotic regimes, noise has little or no effect on λ, while at the border between periodic and chaotic regimes ($\alpha \sim 420$), λ substantially differs whether noise is present or not. The noise seems to affect the transition between different types of attractors.

The chaotic growing system indicates that an exponentially growing system can have a complex trajectory that is unpredictable in the long-term. Still, the long-term growth rate of the system

converges. We conclude that while the trajectory is sensitive to small perturbations, the long-term growth rate, which is a statistical averaging on the projected system, can be still be robust.

5. Asymptotically scalable reaction networks

The condition (C3) of scalable flux requires that the flux function satisfies $J(cx) = cJ(x)$ for a scalar $c > 0$. This is a relatively strong condition that reduces the degrees of freedom of the function by one dimension. Here, we show that a more general class of flux functions, referred to as *asymptotically scalable functions*, gives a similar convergence property on long-term growth rate.

Definition 5.1: A flux function $J_a(x)\colon Q^n \to \mathbb{R}$ is *asymptotically scalable*, if:

(C1)(*differentiability*): $J_a(X)$ is positive in Q^{n+} and continuously differentiable in $Q^n \setminus \{0\}$.
(C2)(*upstream limited*): If the node x_k is one of the upstream nodes of ϕ_a, then $J_a(X) = 0$ whenever $X_k = 0$.
(C3')(*asymptotic scaling*): For every scalar c_1 and $c_2 > 0$ and every vector $x \in Q^n$,

$$\lim_{c_1, c_2 \to \infty} \frac{J_a(c_1 x)}{J_a(c_2 x)} = \frac{c_1}{c_2}$$
$$\lim_{c_1, c_2 \to 0} \frac{J_a(c_1 x)}{J_a(c_2 x)} = \frac{c_1}{c_2} \tag{23}$$

Note that the (C1) and (C2) conditions are identical to the scalable function definition, while condition (C3') is more general than condition (C3). ■

Examples of asymptotic scalable flux functions: (x_j denotes the upstream node)

1. $\frac{p(X_k)}{q(X_k)}\ X_j$, where $p(X), q(X)$ are polynomials $c_i X^i + \ldots + c_n X^n$ with $c_k > 0$ for $k = i, \ldots, n$.
2. $[M + ae^{-bX_p^c}]\ X_j$, with $M, a, b > 0, c \geq 1$.
3. $[M + \tan^{-1}(aX_k)]\ X_j$, with $M > \pi/2$.
4. $\left[M + \sin(\frac{a+X_p}{b+X_p})\right] X_j$, with $M > 1,\ a, b > 0$.

In the following section, we denote $\tilde{f}_a(Y, N) \equiv f_a(X)$ to be the function f in (Y, N) coordinates.

Proposition 5.2: If $J_a(X)$ is asymptotically scalable, then there exists two limiting functions $J_{a,0}(y)\colon \Delta^{n-1} \to \mathbb{R}$ and $J_{a,\infty}(y)\colon \Delta^{n-1} \to \mathbb{R}$ such that the following point-wise convergence holds:

$$\lim_{N \to 0} \frac{\tilde{J}_a(Y, N)}{N} = J_{a,0}(Y)$$
$$\lim_{N \to \infty} \frac{\tilde{J}_a(Y, N)}{N} = J_{a,\infty}(Y) \tag{24}$$

Proof: Consider a fixed vector $Y^* \in \Delta^{n-1}$ and define a function $h_{Y^*}(N) \equiv J_a(NY^*)/N$ on the ray $\{bY^*\}, b > 0$. We have

$$\frac{h_{Y^*}(c_1 N)}{h_{Y^*}(c_2 N)} = \frac{\frac{J_a(c_1 NY^*)}{c_1 N}}{\frac{J_a(c_2 NY^*)}{c_2 N}} \to 1 \tag{25}$$

as $c_1, c_2 \to 0$, by property (C3'). Hence, $h_{Y^*}(N)$ approaches a constant as $N \to 0$, as denoted by $J_{a,0}(Y^*)$. Since the above argument works for every direction Y^*, this defines the function $J_{a,0}(Y)$ on Δ^{n-1}. A similar argument holds for $J_{a,\infty}(Y)$. ■

Assume that the limiting functions $J_{a,0}(Y), J_{a,\infty}(Y)$ are continuously differentiable, which can be checked directly. Using the same stoichiometry matrix S, we define

$$\begin{gathered} F_{k,0}(Y) = \sum_{a=1}^{m} S_{ka} J_{a,0}(Y) \\ F_{k,\infty}(Y) = \sum_{a=1}^{m} S_{ka} J_{a,\infty}(Y) \end{gathered} \tag{26}$$

and let $F^{(0)} \equiv (F_{1,0}, \ldots, F_{n,0})^T$, $F^{(\infty)} \equiv (F_{1,\infty}, \ldots, F_{n,\infty})$, $\mu^{(0)}(Y) \equiv \sum_{j=1}^{n} F_{j,0}(y)$, $\mu^{(\infty)}(Y) \equiv \sum_{j=1}^{n} F_{j,\infty}(Y)$. We obtain the following proposition, which is analogous to Theorem 2.3:

Proposition 5.3: Given an asymptotically scalable reaction network with the initial condition $X(0) \in Q^n$, consider the long-term dynamics of $Y(t)$:

(i) If $\limsup_{t\to\infty} Y(t) = \liminf_{t\to\infty} Y(t) = \infty$, then the long-term dynamics are governed by

$$\frac{dY}{dt} = F^{(\infty)}(Y) - \mu^{(\infty)}(Y)Y \tag{27}$$

(ii) If $\limsup_{t\to\infty} Y(t) = \liminf_{t\to\infty} Y(t) = 0$, then the long-term dynamics are governed by

$$\frac{dY}{dt} = F^{(0)}(Y) - \mu^{(0)}(Y)Y \tag{28}$$

(iii) If $0 < \liminf_{t\to\infty} Y(t) \le \limsup_{t\to\infty} Y(t) < \infty$, then we have $\lambda = 0$. ■

Remark: The case (i) in the above proposition indicates that the system trajectory $X(t) = (Y, N)(t)$ diverges to infinity along the N-coordinate. Hence, the long-term behavior of $Y(t)$ is asymptotically determined by the limiting function $F^{(\infty)}$. If $Y(0)$ is regular with respect to an ergodic probability measure ω, then $\lambda = \int_{\Delta^{n-1}} \mu^{(\infty)}(Y) d\omega$. Similar arguments are also applicable for case (ii), where the trajectory $(Y, N)(t)$ converges to 0 along the N-coordinate. For case (iii), we obtain $\lambda = 0$ directly since $N(t)$ is bounded.

Finally, there are cases not listed above, which are $0 = \liminf_{t\to\infty} Y(t) < \limsup_{t\to\infty} Y(t)$ and $\liminf_{t\to\infty} Y(t) < \limsup_{t\to\infty} Y(t) = \infty$; in these cases, convergence of λ is inconclusive.

6. Autocatalytic flux networks

One of the essential features of living systems is the existence of autocatalysis in suitable environments. Scalable reaction networks with positive growth rates can be used to address a range of questions related to autocatalytic systems. For example, what are the fundamental motifs in an autocatalytic flux network, and can we draw inferences about the long-term growth dynamics of a network by examining its topological structure?

In an SRN, one can consider the growth rates of individual nodes or fluxes as $\lambda[X_p] \equiv \lim_{t\to\infty}(1/t)\log X_p$ or $\lambda[J_a] \equiv \lim_{t\to\infty}(1/t)\log J_a$, when the limit exists. Given an SRN with $\lambda > 0$, not every node x_j in the network would be expected to grow as fast as the system size *N*. If a node x_j grows slower than the system, we have $Y_j \to 0$ as $t \to \infty$. In this case, we say that the node x_j does not contribute to the growth of the system. Our goal is to characterize the nodes that contribute to system growth and to study the mutual dependence of growth rate between fluxes and nodes.

To describe the structure of a flux network (x, ϕ), we adopt the following terminology: A node x_k is *upstream* of a reaction ϕ_a, if $S_{ka} < 0$. A node x_k is *downstream* of a reaction ϕ_a, if $S_{ka} > 0$. Given a reaction ϕ_a, let $up(\phi_a), dw(\phi_a)$ denote the collection of its upstream and downstream nodes, respectively. A reaction ϕ_a is an *influx* of node x_k, if $x_k \in dw(\phi_a)$. A reaction ϕ_a is an *efflux* of node x_k, if $x_k \in up(\phi_a)$. Given a node x_k, let $in(x_k), out(x_k)$ denote the collection of its influxes and effluxes, respectively.

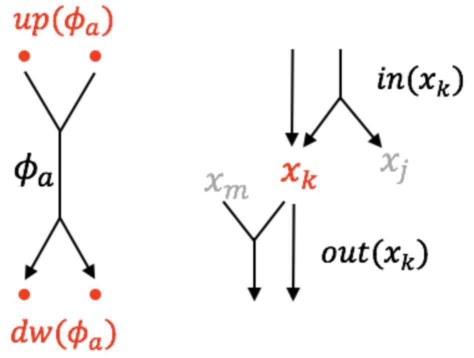

The above definition only involves the network connection; it is not related to the flux function set J. If we associate a flux function set to a flux network (x, ϕ, J), then we can introduce the following concept of "maintenance set":

Definition 6.1: Let $x = \{x_1, \dots, x_n\}$ denote nodes in the system. We say a node x_k *maintains* the reaction ϕ_a, if $X_k = 0$ implies $J_a(X) = 0$. The collection of nodes that maintains ϕ_a is called the *maintenance set* of reaction ϕ_a, denoted by $mt(\phi_a)$. ∎

Remark: One reaction can have zero, one, or multiple maintenance nodes. For a scalable reaction flux, all of its upstream nodes are in the maintenance set (by condition C2). Still, there may be additional nodes that are not upstream of a reaction but do maintain it, such as, for example, an enzyme that is necessary for a reaction flux but is not consumed in the reaction.

For SRNs, the only possible scenario for a reaction ϕ_a to have no maintenance set is the case where the only upstream node is the environment $\{E\}$. For example, consider the reaction $\phi_a: \{E\} \to x_3$, and the associated flux function $J_a(X) = X_1 + X_2$. Biologically, X_1 and X_2 are redundant transporters that import x_3 from the environment into the system. For this case, $mt(\phi_a)$ is empty since x_1 and x_2 are redundant in the flux function. In this case, if we split the reaction ϕ_a into two reactions ϕ_{a1} and ϕ_{a2} and associate two flux functions $J_{a1} \equiv X_1, J_{a2} \equiv X_2$, then we have $mt(\phi_{a1}) = x_1$ and $mt(\phi_{a2}) = x_2$.

Practically, we can always modify the reaction set and split the flux function such that the maintenance set is nonempty under this new representation. This can be done by noticing that $J_a(X) = J_a(Y)N = J_a(Y)X_1 + \cdots + J_a(Y)X_n$ and by setting $J_{aj}(X) = J_a(Y)X_j$. It can be checked that $J_{aj}(X)$ are scalable functions and $mt(\phi_{aj})$ is nonempty. In general, the method to split a flux function is not unique, and we would like to choose the representation that best fits the biological intuition. In the following discussion, we assume that all reactions have a nonempty maintenance set.

Definition 6.2: Given a scalable reaction network (x, ϕ, J), a collection of fluxes $K \equiv \{\phi_{a1}, \dots, \phi_{an}\}$ is called an *autocatalytic circuit*, if

$$\bigcup_{\phi_{aj} \in K} mt(\phi_{aj}) \subseteq \bigcup_{\phi_{aj} \in K} dw(\phi_{aj}) \tag{29}$$

We can examine every reaction ϕ_a and list its maintenance set and its downstream nodes. For a flux collection K, we can verify whether K is an autocatalytic circuit by performing simple logical operations. ■

Note that if K is not an autocatalytic circuit, it is still possible to have a proper subset $K' \subset K$ such that K' is autocatalytic. On the other hand, if K is autocatalytic, it is also possible to have a proper subset $K' \subset K$ which is autocatalytic. The following simple algorithm allows us to examine whether a flux collection K contains any autocatalytic circuit $K' \subseteq K$. We denote $mt(K) \equiv \bigcup_{\phi_a \in K} mt(\phi_a)$ and $dw(K) \equiv \bigcup_{\phi_a \in K} dw(\phi_a)$.

Algorithm 6.3*:* Consider a flux network (x, ϕ, J) and an initial flux set K_1.

(1) For $j \geq 1$, calculate $mt(K_j)$ and $dw(K_j)$. If $mt(K_j) \subseteq dw(K_j)$, an autocatalytic circuit K_j is found and the algorithm terminates. If $mt(K_j) \nsubseteq dw(K_j)$, examine each reaction $\phi_a \in K_j$ and test if $mt(\phi_a) \in dw(K_j)$. Remove every reaction ϕ_a such that $mt(\phi_a) \notin dw(K_j)$, and obtain a smaller reaction set K_{j+1}.

(2) Repeat the above procedure until either K_j is empty or an autocatalytic circuit is found. If the algorithm reaches an empty set, then there is no autocatalytic circuit in K_1.

Proof: Suppose K has no autocatalytic subset, the above algorithm will yield an empty set. Suppose K has an autocatalytic circuit K', we need to show every reaction $\phi_a \in K'$ will not be removed by this algorithm. Since $mt(\phi_a) \subseteq mt(K') \subseteq dw(K')$, ϕ_a will not be removed at step $j = 1$. Suppose that we have $K' \subseteq K_j$ up to step j. We have $mt(\phi_a) \subseteq mt(K') \subseteq dw(K') \subseteq dw(K_j)$ and hence ϕ_a will not be removed in step j. Therefore, K' will be a subset of K_{j+1}. By

induction on j, the reactions in the subset K' will not be removed by the algorithm. Hence, after at most m steps (m is the number of reactions in the system), the algorithm reaches a stable reaction set K^* with $mt(K^*) \subseteq dw(K^*)$ and $K' \subseteq K^*$. ■

The following Lemma establishes a simple relation satisfied by scalable fluxes, with several corollaries, which will be useful in proving our main result on autocatalytic circuits below (Thm. 6.5).

Lemma 6.4: If a node $x_p \in mt(\phi_a)$, then the flux function takes the form $J_a(X) = R_{a,p}(Y)X_p$ where $R_{a,p}(Y)$ is a continuous function defined on Δ^{n-1}.

Proof: We define $R_{a,p}(Y) \equiv J_a(Y)/Y_p = J_a(X)/X_p$ which is well-defined and continuous for $Y_p > 0$. Extension of $R_{a,p}(Y)$ to the entire simplex is possible only if $J_a(Y) \to 0$ as $Y_p \to 0$, i.e. if $x_p \in mt(\phi_a)$, and we define $R_{a,p}(Y) \equiv (\partial/\partial Y_p)J_a(Y)\big|_{Y_p=0}$ for $Y_p = 0$, where the derivative exists by scalability condition C1. ■

Corollary 6.4a: For a SRN, if $Y(0)$ is regular for some ergodic measure ω then the node growth rates $\lim_{t\to\infty}(1/t)\log X_p$ exist for all nodes x_p of the network.

Proof: We can express the dynamics of X_p in terms of the influxes and effluxes of node x_p as $dX_p/dt = F_p(X) = \sum_{a:\phi_a \in in(x_p)} |S_{pa}|J_a(X) - \sum_{a:\phi_a \in out(x_p)} |S_{pa}|J_a(X)$. Using Lemma 6.4 for the effluxes, we have

$$\frac{1}{X_p}\frac{dX_p}{dt} = \frac{F_p(Y)}{Y_p} = \frac{1}{Y_p}\sum\nolimits_{a:\phi_a \in in(x_p)} |S_{pa}|J_a(Y) - \sum\nolimits_{a:\phi_a \in out(x_p)} |S_{pa}|R_{a,p}(Y) \qquad (30)$$

The second summation is a continuous function on the entire simplex hence integrable, while the first summation is non-negative and continuous on the simplex interior; thus the integral of $F_p(Y)/Y_p$ exists and takes values in $\mathbb{R} \cup +\infty$. We can therefore apply the ergodic theorem (which applies without the L^1 assumption for functions that are bounded from below) to obtain

$$\lim_{t\to\infty}\frac{1}{t}\log X_p = \lim_{t\to\infty}\frac{1}{t}\int_0^t \frac{X_p'(s)}{X_p(s)}ds = \lim_{t\to\infty}\frac{1}{t}\int_0^t \frac{F_p(Y(s))}{Y_p(s)}ds = \int \frac{F_p(Y)}{Y_p}d\omega \qquad (31)$$

and since node growth rates are bounded by the system's long-term growth rate λ, which exists and is finite by Thm. 2.3, the above limit is finite. ■

Corollary 6.4b: For an SRN with long-term growth rate $\lambda > 0$, if a flux J_a has growth rate λ, then all of its maintenance nodes x_p grow at rate λ.

Proof: Let x_p be a maintenance node of J_a. By Lemma 6.4, $J_a(X) = R_{a,p}(Y)X_p$. Since $R_{a,p}(Y)$ is a bounded non-negative function, positive growth of J_a implies that X_p must grow at least as fast as J_a. Since J_a is growing at the same rate as the whole system and X_p is a component of the system, X_p must be growing exactly at the rate λ. ■

Theorem 6.5: Consider an SRN (x, ϕ, J) with an initial condition $Y(0)$, which is regular for an ergodic measure ω. If the long-term growth rate λ is positive, then there exists at least one autocatalytic circuit K in the network such that every node $x_j \in mt(K)$ has a long-term growth rate λ.

Proof: By Cor. 6.4a, all node growth rates $\lambda[X_p]$ exist. We provide a recursive procedure to find an autocatalytic circuit K satisfying Eq. (29). Given a network with growth rate $\lambda > 0$, there is at least one node x_p with $\lambda[X_p] = \lambda$, and it is clear that there exists at least one reaction $\phi_{(01)} \in in(x_p)$ with $\lambda[J_a] = \lambda$. We denote $K_0 \equiv \{\phi_{(01)}\}$.

Below we provide a procedure that starts with a collection of reactions K_j all of which have growth rate λ, and enlarges the collection to K_{j+1}. Let $K_j = \{\phi_{(j1)}, \ldots, \phi_{(jn)}\}$. For each flux $\phi_{(ja)}$, we examine its maintenance set $mt(\phi_{ja}(X))$. If $mt(\phi_{ja})$ is nonempty, all nodes $x_p \in mt(\phi_{ja})$ have the same growth rate λ based on Cor. 6.4b, thus every node x_p has at least one influx with growth rate λ. (If there are multiple influxes with growth rate λ, we are free to choose any of them.) We denote $\phi[x_p]$ to be the influx chosen from $in(x_p)$, as shown here:

$$\boldsymbol{\phi_a \dashrightarrow x_p \in mt(\phi_a) \dashrightarrow \phi[x_p] \in in(x_p)}$$

Now, we define

$$K_{j+1} \equiv K_j \cup \left(\bigcup_{\phi_{(ja)} \in K_j} \; \bigcup_{x_p \in mt\{\phi_{(ja)}\}} \phi\left[x_p\right] \right) \qquad (31)$$

noting that all fluxes in K_{j+1} have growth rate λ. By this procedure, the size $\{K_j\}$ monotonically increases. Since the total number of reactions in the network is finite, there exists a maximal collection K such that K is invariant under this procedure. Next, we must show that $mt(K) \subseteq dw(K)$, i.e., K is an autocatalytic circuit.

Given an arbitrary reaction $\phi_a \in K$, suppose that this reaction is recruited into K at step j for the first time. For each node $x_k \in mt(\phi_a)$, there exists at least one reaction that will be recruited into K at step $j+1$, denoted as $\phi[x_k]$ (if this reaction does not yet belong to K_j). Hence, $x_k \in dw(\phi[x_k]) \subset dw(K)$. This shows $mt(\phi_a) \in dw(K)$ for an arbitrary reaction $\phi_a \in K$, and therefore, K is an autocatalytic circuit.

By construction, every flux J_a associated with $\phi_a \in K$ has a growth rate $\lambda > 0$. By Cor. 6.4b, all maintenance nodes of K has a growth rate λ. ■

Remark 1: The maximal collection K constructed above is not necessarily unique. There may be several redundant autocatalytic circuits in the flux network. Each of them can be constructed by choosing a different initial reaction $\phi_{(01)}$ or a different $\phi[x_k]$ during the recursion steps.

Remark 2: The condition $\lambda > 0$ is essential in Theorem 6.5. If the system has zero or negative long-term growth rate, the statements in Propositions 6.4a and 6.4b no longer hold. That is, the growth rate of each node in an autocatalytic circuit does not necessarily need to be the same.

This method is remarkable in the sense that we do not need to solve the differential equation (either analytically or numerically). The procedure only requires that we inspect each flux

function and obtain its maintenance set. The remaining computation is purely algebraic. It is the flux network connection and the property of positive growth rate that constrain the growth rate between nodes and fluxes.

Example 6.6: Random networks

To characterize random networks, we define P_{auto} as the probability that a random reaction network is autocatalytic, which depends on its statistical properties. Intuitively, for Eq. 6 to be satisfied, one should decrease the average number of $mt(\phi_a)$ and increase the average number of $dw(\phi_a)$. Furthermore, given a random reaction ϕ_a, the chance of a given node to belong to $mt(\phi_a)$ or $dw(\phi_a)$ could be different. Intuitively, if some nodes acted as "hubs" to maintain many other nodes, the probability P_{auto} should be higher (Fig. S6A). In contrast, if a node required many other upstream nodes (Fig. S6B), P_{auto} should be lower.

To test how network topology affects P_{auto}, we constructed an ensemble of random reaction networks with a fixed number of nodes (*n* = 100), but with different connections of reactions. Consistent with our expectation, we found that P_{auto} transitions from 0 to 1 as the number of reactions increases in the network (Fig. S6). To see how hub-like connections affect P_{auto}, we randomly sampled the maintenance and downstream nodes of every reaction from power-law distributions with exponents γ_{mt} and γ_{dw}. We found that P_{auto} is strongly affected by both exponents. For example, network connections with uniform probability ($\gamma_{mt} = \gamma_{dw} = 0$) usually required 150 or more reactions to be autocatalytic (Fig. S6A). However, for a hub-like maintenance set with skewed probability $\gamma_{mt} = 2$, the number of reactions required could be reduced to 100. This contrasts with a hub-like downstream set with skewed probability $\gamma_{dw} = 1$, for which more than 400 reactions were required for the network to be autocatalytic.

7. Appendix

Existence and uniqueness of solutions for Eq. (19)

In this section, we denote $|.|$ as the l^1 norm in Euclidean space. We verify the following conditions in Ref(47),

(A1) $|F^{(Ito)}(X)| + |H(X)| \leq C_1(1 + |X|)$

(A2) $|F^{(Ito)}(X) - F^{(Ito)}(X')| + |H(X) - H(X')| \leq C_2|X - X'|$

for all $X, X' \in Q^n$ and positive constants C_1, C_2.

The conversion between Ito and Stratonovich integrals is described by Eq. (20). To show (A1), we obtain $|F_k(X)| = |X||F_k(Y)|$ because of the scalable condition (C3). According to the continuous differentiability condition (C1) and the compactness of Δ^{n-1}, there exists a constant $B_F > 0$ such that $|F_k(Y)| \leq B_F$ for all $Y \in \Delta^{n-1}$ and for every k. The function $H_k(X)$ is bounded similarly by a constant B_H.

Next, by the scalable property (N3), the value of $\frac{\partial H_k}{\partial X_k}$ is scale-invariant (i.e., $\frac{\partial H_k(cX)}{\partial(cX_k)} = \frac{\partial H_k(X)}{\partial X_k}$ for all $c > 0$). Hence, this value is specified by $\frac{\partial H_k}{\partial X_k}(Y)$ on the unit simplex Δ^{n-1}. Since H is continuously differentiable on the simplex, this term is again bounded by a constant. Hence, the drift correction term $\frac{\partial H_k}{\partial X_k} H_k$ is bounded by $B_{drift}|X|$ for all X and all k, with constant $B_{drift} > 0$. Together, we can choose $C_1 = n(B_F + B_{drift} + B_H)$ and condition (A1) is satisfied.

To show (A2), we use the mean value theorem to get $F_k(X) - F_k(X') = \nabla F_k(X^*)(X - X')$ with some X^* at the interior of segment $\overline{XX'}$. Note that the gradient of the scalable function is scale-invariant. Hence, because of the continuity condition (C1), there is a positive constant $B_{\nabla F}$ as upper bound of $\frac{\partial F_k(Y)}{\partial X_{k'}}$ for all $Y \in \Delta^{n-1}$ and for all k, k'. Hence, we have

$$|F_k(X) - F_k(X')| \leq |\nabla F_k(X^*)||X - X'| = \left|\nabla F_k\left(\frac{X^*}{|X^*|}\right)\right| |X - X'| \leq B_{\nabla F}|X - X'| \qquad (32)$$

A similar argument holds for H. For the drift correction term, note that

$$\begin{aligned} \frac{\partial H_k(X)}{\partial X_k} H_k(X) - \frac{\partial H_k(X')}{\partial X_k} H_k(X') &= \frac{1}{2}[H_k(X) + H_k(X')]\left[\frac{\partial}{\partial X_k}\big(H_k(X) - H_k(X')\big)\right] \\ &+ \frac{1}{2}[H_k(X) - H_k(X')]\left[\frac{\partial}{\partial X_k}\big(H_k(X) + H_k(X')\big)\right] \end{aligned} \qquad (33)$$

Using similar arguments to those mentioned above, the difference of the drift-correction term can be bounded by $B_{drift'}|X - X'|$ with a constant $B_{drift'} > 0$. Together, we can choose $C_2 = n(B_{\nabla F} + B_{drift'} + B_{\nabla H})$ and condition (A2) is satisfied.

Proof of Proposition 3.2:

We will use the following two theorems. We denote $Q_k \equiv \{X: X_k = 0\}$ be a plane in $\mathbb{R}^n$ and $\overline{Q_k} \equiv \{X: X_k > 0\}$.

Theorem 3.2.1 [Ref(48), Thm 1.1]: For an SDE with the diffusion operator $\mathcal{A}^* = \sum_{i,j} D_{ij}(X) \frac{\partial^2}{\partial X_i \partial X_j} + \sum_j v_j(X) \frac{\partial}{\partial X_j}$, if there exists a real-valued function $V(X)$ that satisfies the following conditions

(1) there exists a constant $C > 0$ such that $\mathcal{A}^* V \leq CV$ for all $X \in \overline{Q_k}$,

(2) $V(X) \to \infty$ whenever $dist(X, Q_k) \to 0$,

then a trajectory starting with $X_k(0) > 0$ cannot reach Q_k at a finite time.

The next theorem states that for an SDE satisfying the above criteria, the function $V(X)$ in the above theorem can be found explicitly.

Theorem 3.2.2 [Ref(48). Thm 1.1, Ref(49), Thm 9.4.1]: Consider the SDE in Theorem 3.2.1, with diffusion operator $D_{ij}(X)$ and deterministic flow $v_k(X)$. The function $V(X)$ can be chosen as $V(X) \equiv \frac{1}{|dist(X,Q_k)|^{\epsilon}}$ for a fixed $\epsilon > 0$, if the following criteria are satisfied:

(A1) $|F_k^{(Ito)}(X) + H_k(X)| \leq C_1(1 + |X|)$ for a constant $C_1 > 0$ and for all X.

(A2) $|F_k^{(Ito)}(X) - F_k^{(Ito)}(X')| + |H_k(X) - H_k(X')| \leq C_2|X - X'|$ for a constant $C_2 > 0$ and for all X, X'.

(A3) If $X \in Q_k$, then $D_{kk}(X) = 0$.

(A4) If $X \in Q_k$, then $\left(v_k(X) - \frac{1}{2}\sum_{i=1}^{n} \frac{\partial D_{ki}(X)}{\partial X_i}\right) \geq 0$.

The quantity within the parenthesis in (A4) is called the *Fichera drift*.

To proceed, we show that the conditions (A1-A4) hold for the scalable SDE (19). Conditions (A1) and (A2) were demonstrated to hold above. To verify (A3) and (A4), we calculate the diffusion matrix and drift vector by standard SDE rules ([Ref(47)]):

$$\begin{gathered} D_{kj}(X) = \frac{1}{2}\delta_{kj}[H_k(X)]^2 \\ v_k(X) = F_k(X) + \frac{1}{2}\frac{\partial H_k(X)}{\partial X_k}H_k(X) \end{gathered} \tag{34}$$

Since the matrix D is diagonalized, we have $D_{kk}(X) = [H_k(X)]^2$ and condition (N2) implies condition (A3). For condition (A4), we first notice that the upstream-limited condition of flux (C2) implies that $F_k(X) \geq 0$ whenever $X_k = 0$. Second, $H_k(X) = 0$ on Q_k, while $\frac{\partial H_k}{\partial X_k}$ is bounded by a constant. Together, we have $v_k(X) \geq 0$ on Q_k. Finally, we obtain

$$\sum_{i=1}^{n} \frac{\partial D_{ki}(X)}{\partial X_i} = \frac{\partial D_{kk}(X)}{\partial X_k} = \frac{\partial [H_k(X)]^2}{\partial X_k} = H_k(X)\frac{\partial H_k(X)}{\partial X_k} \tag{35}$$

As above, this term is zero on Q_k. This shows that the Fichera drift on Q_k satisfies the condition in (A4).

We have shown that for the scalable SDE, conditions (A1) to (A4) are satisfied. By Theorems 3.2.1 and 3.2.2, for any trajectory $X(t)$ with $X_k(0) > 0$, the system does not reach the plane Q_k at a finite time. This implies that $N = X_1 + \ldots + X_n$ does not reach the origin at a finite time. Together, the trajectory $Y(t) = X(t)/N(t)$ with $Y_k(0) > 0$ does not reach a simplex boundary

at a finite time. Along the proof, we have shown that $v_k(X) \geq 0$ and the component X_k becomes deterministic on Q_k. Hence the trajectory $Y(t)$ is confined in the unit simplex. ∎

Methods: Simulation and analysis of scalable reaction networks

1. Numerical solution of ordinary differential equations

All ordinary differential equations (ODE) were integrated by MATLAB function *ode45* with relative tolerance $RelTol = 10^{-3}$ and absolute tolerance $RelTol = 10^{-6}$. In Fig. 2b, c, and g, the trajectory $Y(t)$ was also calculated by Mathematica function *NDSolve* with default accuracy, yielding consistent results. In Fig. S2, the trajectory $Y(t)$ was integrated by a log-transform ODE for improving the accuracy when Y_k is close to zero. For calculating the long-term growth rate λ, we simulated the trajectory $Y(t)$ for a sufficiently long time such that $Y(t)$ converges to its attractor. After convergence, we averaged the instantaneous growth rate $\mu(Y(t_j))$ with time step = 0.01 (time unit) to obtain growth rate λ. To simulate a system with noise, the differential equation was integrated with an additional Gaussian noise term for every integration time step.

2. Calculation of Poincare section and bifurcation analysis

The Poincare section and bifurcation analysis in Fig. S3A were calculated using the following procedure:

1. A Poincare section plane $\{Y_P = c_P\}$ and a projection coordinate Y_a are chosen.

2. The ODE system is integrated from time 0 to a large time $T_{Pre} = 2000$ to allow the trajectory to converge to the attractor. The final position $Y^{(eq)}$ of the trajectory is used as the initial condition in the next step.

3. The ODE system is integrated from time 0 to a large time $T_L = 1000$, with initial condition $Y(0) = Y^{(eq)}$ and a given parameter κ. This generates a trajectory $\{Y(t_j)\}$ with time step size Δt = 0.02. When a time point t_j satisfied

$$Y_P(t_j) \leq c_P \text{ and } Y_P(t_{j+1}) > c_P,$$

the coordinate $Y_a(t_j)$ is recorded. This procedure gave a list of scalars $\{Y_a(t_{j1}),\ldots, Y_a(t_{jC})\}$.

4. The same procedure as in 3 is repeated with a different parameter α. For each α, a list of points $\{ \left(\alpha, Y_a^{(\alpha)}(t_{j1})\right),\ldots, \left(\alpha, Y_a^{(\alpha)}(t_{jC})\right)\}$ is obtained on the $\alpha - Y_a$ plane. The two-dimensional scatter plot shown in Fig. S3B was generated by using all α values and all points in their lists.

3. Calculation of the largest Lyapunov exponent

The largest Lyapunov exponent (LLE) in Fig. S3B was calculated by the following procedure:

1. The ODE system is integrated for a long-time interval from time 0 to 2000 to allow the trajectory to converge to the attractor. The final position $Y^{(eq)}$ of trajectory is used as the initial condition in the next step.

2. We set $Y_{start} \leftarrow Y^{(eq)}$

$$Y_{start}^{(pert)} \leftarrow Y^{(eq)} + \epsilon * (\delta Y)$$

where (δY) is an isotropic random vector with $|\delta Y| \leq 0.1$ and $\sum_j (\delta Y)_j = 0$.

3. The system with the initial condition Y_{start} is integrated for a short time interval [0, τ] with τ=20 to obtain $Y(\tau)$. Similarly, the system with the initial condition $Y_{start}^{(pert)}$is integrated for a short time interval [0, 20] to obtain $Y^{(pert)}(\tau)$. Using these results, the exponent of trajectory divergence E_1 is calculated as follows:

$$E_1 \equiv \frac{1}{\tau} log\left(\frac{\|Y^{(pert)}(\tau)-Y(\tau)\|}{\left\|Y_{start}^{(pert)}-Y_{start}\right\|}\right)$$

4. The parameter is reset as $Y_{start} \leftarrow Y(\tau)$

$$Y_{start}^{(pert)} \leftarrow Y(\tau) + \epsilon * \left(\frac{Y^{(pert)}(\tau)-Y(\tau)}{\|Y^{(pert)}(\tau)-Y(\tau)\|}\right).$$

with $\epsilon = 0.1$ and the procedure described by step 3 is repeated to obtain another pair of trajectories $Y(\tau)$ and $Y^{(pert)}(\tau)$. The trajectory divergence E_2 is calculated similarly. This aforementioned procedure is repeated 50 times to obtain a list of values $\{E_1, \dots, E_{50}\}$. The average divergence E is calculated by the algebraic averaging of E_j.

5. The procedure in steps 2 to 4 is repeated five times, where each repeat has different random perturbations and yields a different exponential divergence $E^{(k)}$. The LLE shown in Fig. S3B is calculated by averaging $E^{(k)}$ from all simulations.

Note that for choosing the proper values for constants s and τ, they should satisfy

$$\left\|Y_{start}^{(pert)} - Y_{start}\right\| \ll W$$

$$\left\|Y^{(pert)}(\tau) - Y(\tau)\right\| \ll W$$

$$\left|\frac{\|Y^{(pert)}(\tau) - Y(\tau)\|}{\left\|Y_{start}^{(pert)} - Y_{start}\right\|} - 1\right| \gg \epsilon$$

where W is the characteristic length scale of the attractor and ϵ is the numerical accuracy of the computation. (For the autocatalytic double repressilator, we chose $\tau = 20$ and $s = 0.1$.)

4. Mathematical models of scalable reaction networks

Parameters shown in blue are the ones we varied in this study.

(i) Autocatalytic single-repressilator model

The autocatalytic single-repressilator model has four nodes X_1 to X_4 (see Fig 3A and the main text), with the following reactions:

Reaction	Flux function
ϕ_1: {env}→ x_1	$J_1 = b_2X_2 + b_3X_3 + b_4X_4$
ϕ_2: $x_1 \rightarrow x_2$	$J_2 = \frac{c_{syn}X_1}{1 + KY_3^{\theta}}$
ϕ_3: $x_1 \rightarrow x_3$	$J_3 = \frac{c_{syn}X_1}{1 + KY_4^{\theta}}$
ϕ_4: $x_1 \rightarrow x_4$	$J_4 = \frac{c_{syn}X_1}{1 + KY_2^{\theta}}$
ϕ_5: $x_2 \rightarrow \{\emptyset\}$	$J_5 = d_2X_2$
ϕ_6: $x_3 \rightarrow \{\emptyset\}$	$J_6 = d_3X_3$
ϕ_7: $x_4 \rightarrow \{\emptyset\}$	$J_7 = d_4X_4$

Parameters used in simulations were as follows:

$b_2 = 0.5$ (1/*time*)	$d_2 = 0.25$ (1/*time*)
$b_3 = 0.4$ (1/*time*)	$d_3 = 0.3$ (1/*time*)
$b_4 = 0.3$ (1/*time*)	$d_4 = 0.2$ (1/*time*)
$c_{syn} = 20$ (1/*time*)	$K = 10 - 10000$
	$\theta = 1 - 4$

(ii) Autocatalytic double-repressilator model

The autocatalytic double-repressilator model has seven nodes X_1 to X_7 (see Fig. 3F and the main text), with the following reactions:

Reaction	Flux function
ϕ_1: {env}→ x_1	$J_1 = b_2X_2 + b_3X_3 + b_4X_4 + b_5X_5 + b_6X_6 + b_7X_7$

$\phi_2\!: x_1 \to x_2$	$J_2 = \frac{cX_1}{1 + K_a Y_3^\theta} \frac{1}{1 + MY_5^\phi}$
$\phi_3\!: x_1 \to x_3$	$J_3 = \frac{cX_1}{1 + K_a Y_4^\theta}$
$\phi_4\!: x_1 \to x_4$	$J_4 = \frac{cX_1}{1 + K_a Y_2^\theta}$
$\phi_5\!: x_1 \to x_5$	$J_5 = \frac{cX_1}{1 + K_b Y_6^\theta} \frac{1}{1 + MY_2^\phi}$
$\phi_6\!: x_1 \to x_6$	$J_6 = \frac{cX_1}{1 + K_b Y_7^\theta}$
$\phi_7\!: x_1 \to x_7$	$J_7 = \frac{cX_1}{1 + K_b Y_5^\theta}$
$\phi_8\!: x_2 \to \{\emptyset\}$	$J_8 = d_2 X_2$
$\phi_9\!: x_3 \to \{\emptyset\}$	$J_9 = d_3 X_3$
$\phi_{10}\!: x_4 \to \{\emptyset\}$	$J_{10} = d_4 X_4$
$\phi_{11}\!: x_5 \to \{\emptyset\}$	$J_{11} = d_5 X_5$
$\phi_{12}\!: x_6 \to \{\emptyset\}$	$J_{12} = d_6 X_6$
$\phi_{13}\!: x_7 \to \{\emptyset\}$	$J_{13} = d_7 X_7$

Parameters used in simulations are as follows:

$b_2 = 0.9\ (1/time)$	$K_a = 1500$
$b_3 = 0.7\ (1/time)$	$\alpha = 1\ to\ 800,\ K_b = \alpha K_a$
$b_4 = 1.0\ (1/time)$	
$b_5 = 0.6\ (1/time)$	$d_2 = 0.2\ (1/time)$
$b_6 = 0.4\ (1/time)$	$d_3 = 0.5\ (1/time)$
$b_7 = 1.2\ (1/time)$	$d_4 = 0.3\ (1/time)$
$c = 10\ (1/time)$	$d_5 = 0.1\ (1/time)$
$\theta = 4$	$d_6 = 0.4\ (1/time)$
$\phi = 4$	$d_7 = 0.6\ (1/time)$
$M = 100$	

(iii) Ecosystem model of three competing and cross-feeding species

Cross-feeding model for a turbidostat-like system (unlimited resource): The three-species model has six nodes: x_1 to x_3 represent three species and m_1 to m_3 represent three metabolites, each secreted by one species (see Fig. 4A in the main text). $X_1, X_2, X_3, M_1, M_2, M_3$ denote the total amount of biomass of species and metabolites and N is defined by $X_1 + X_2 +$

$X_3 + M_1 + M_2 + M_3$. The variables $Y_1, Y_2, Y_3, \widetilde{M_1}, \widetilde{M_2}, \widetilde{M_3}$ represent the relative amount of biomass of species x_1 to x_3 (normalized as $Y_j = X_j/N$ and $\widetilde{M}_j = M_j/N$).

For each species, the influxes from the environment (ϕ_1 to ϕ_3) are modeled by a Lotka-Volterra-like quadratic function. We modified the function to be scalable. The production of secreted metabolites are linear fluxes (ϕ_4 to ϕ_6) that are proportional to the species abundance. There is a total of six cross-feeding fluxes, with each metabolite being able to feed one or both species that do not produce this metabolite. The cross-feeding fluxes are Michaelis-Menten type (ϕ_{12}, $\phi_{13}, \phi_{21}, \phi_{23}, \phi_{31}, \phi_{32}$).

Reaction	Flux function
ϕ_1: {env}→ x_1	$J_1 = r_1 X_1 \left(1 - \sum_{j=1}^{3} a_{1j} Y_j\right)$
ϕ_2: {env}→ x_2	$J_2 = r_2 X_2 \left(1 - \sum_{j=1}^{3} a_{2j} Y_j\right)$
ϕ_3: {env}→ x_3	$J_3 = r_3 X_3 \left(1 - \sum_{j=1}^{3} a_{3j} Y_j\right)$
ϕ_4: $x_1 \to m_1$	$J_4 = b_1 X_1$
ϕ_5: $x_2 \to m_2$	$J_5 = b_2 X_2$
ϕ_6: $x_3 \to m_3$	$J_6 = b_3 X_3$
ϕ_{12}: $m_2 \to x_1$	$J_{12} = c_{12} X_1 \frac{M_2}{KN + M_2}$
ϕ_{13}: $m_3 \to x_1$	$J_{13} = c_{13} X_1 \frac{M_3}{KN + M_3}$
ϕ_{21}: $m_1 \to x_2$	$J_{21} = c_{21} X_2 \frac{M_1}{KN + M_1}$
ϕ_{23}: $m_3 \to x_2$	$J_{23} = c_{23} X_2 \frac{M_3}{KN + M_3}$
ϕ_{31}: $m_1 \to x_3$	$J_{31} = c_{31} X_3 \frac{M_1}{KN + M_1}$
ϕ_{32}: $m_2 \to x_3$	$J_{32} = c_{32} X_3 \frac{M_2}{KN + M_2}$

The system dynamics is governed by the following differential equations:

$$\frac{dX_1}{dt} = J_1 - J_4 + J_{12} + J_{13}$$

$$\frac{dX_2}{dt} = J_2 - J_5 + J_{21} + J_{23}$$

$$\frac{dX_3}{dt} = J_3 - J_6 + J_{31} + J_{32}$$

$$\frac{dM_1}{dt} = J_4, \qquad \frac{dM_2}{dt} = J_5, \qquad \frac{dM_3}{dt} = J_6$$

Parameters used in simulations are as follows:

$r_1, r_2, r_3 = 1.0$ (1/*time*)
$b_1, b_2, b_3 = 0.2$ (1/*time*)
$K = 0.2$
$a_{ij} = 0 - 0.8$ (scalar)
$c_{ij} = 0 - 0.5$ (1/*time*)

For all simulations, the coefficients a_{ij} were sampled from a uniform distribution between [0, 0.8].

For weak and strong cross-feeding, the coefficients c_{ij} were sampled from uniform distributions between [0, 0.1] and [0, 0.5], respectively. For the no cross-feeding cases, the coefficients c_{ij} were set to 0.

Each condition (no cross-feeding, weak cross-feeding and strong cross-feeding) were simulated with a 50,000 random parameter set. For each parameter set, five random initial conditions (uniformly sampled from a simplex space) were simulated. A customized script was developed to classify if the normalized system dynamics ($Y(t)$) reaches a fixed point. Systems with nontrivial dynamics in the long term were subjected to manual inspection and classified as fixed points, limit cycles or heteroclinic cycles. For most of random parameter sets, the model converged to a fixed point. About 0.5% of the cases converged to a heteroclinic cycle, while the limit cycle cases were even rarer (2 cases from the 150,000 random parameter sets).

System parameters for data shown in Fig. 4C:

Initial condition: $(X_1, X_2, X_3, M_1, M_2, M_3)$ = (0.25, 0.25, 0.4, 0.02, 0.03, 0.05)

$a_{ij} = \begin{pmatrix} 0.536 & 0.486 & 0.214 \\ 0.266 & 0.055 & 0.763 \\ 0.774 & 0.654 & 0.182 \end{pmatrix}$	$c_{ij} = \begin{pmatrix} 0 & 0.044 & 0.098 \\ 0.251 & 0 & 0.212 \\ 0.436 & 0.426 & 0 \end{pmatrix}$

System parameter set for data shown in Fig. 4D:

Initial condition: $(X_1, X_2, X_3, M_1, M_2, M_3)$ = (0.1, 0.1, 0.7, 0.05, 0.03, 0.02)

$a_{ij} = \begin{pmatrix} 0.553 & 0.694 & 0.061 \\ 0.408 & 0.348 & 0.531 \\ 0.771 & 0.396 & 0.069 \end{pmatrix}$	$c_{ij} = \begin{pmatrix} 0 & 0.098 & 0.108 \\ 0.378 & 0 & 0.165 \\ 0.030 & 0.108 & 0 \end{pmatrix}$

Cross-feeding model for a chemostat-like system (resource in limited amount): The simulations in Fig. 4B-D were performed using usual SRNs in which there are unlimited upstream resources. For a chemostat-like system in which an essential nutrient is limited, we made several modifications to the turbidostat-like equation in the above section.

We first describe the chemostat equations for the cross-feeding model. Below, we denote the variable $[S]$, $[X_j]$,$[M_j]$ as the *concentrations* (mass per volume) of the external metabolite, the cells, and cross-feeding metabolites. The flux functions are:

Reaction	Flux function
ϕ_1: {env}→ x_1	$J_{1c} = \frac{r_{max}[S]}{K_S + [S]}\ [X_1]\left(1 - \sum_{j=1}^{3} a_{1j}[X_j]\right)$
ϕ_2: {env}→ x_2	$J_{2c} = \frac{r_{max}[S]}{K_S + [S]}\ [X_2]\left(1 - \sum_{j=1}^{3} a_{2j}[X_j]\right)$
ϕ_3: {env}→ x_3	$J_{3c} = \frac{r_{max}[S]}{K_S + [S]}\ [X_3]\left(1 - \sum_{j=1}^{3} a_{3j}[X_j]\right)$
ϕ_4: $x_1 \rightarrow m_1$	$J_4 = b_1[X_1]$
ϕ_5: $x_2 \rightarrow m_2$	$J_5 = b_2[X_2]$
ϕ_6: $x_3 \rightarrow m_3$	$J_6 = b_3[X_3]$
ϕ_{12}: $m_2 \rightarrow x_1$	$J_{12} = c_{12}[X_1]\frac{[M_2]}{K + [M_2]}$
ϕ_{13}: $m_3 \rightarrow x_1$	$J_{13} = c_{13}[X_1]\frac{[M_3]}{K + [M_3]}$
ϕ_{21}: $m_1 \rightarrow x_2$	$J_{21} = c_{21}[X_2]\frac{[M_1]}{K + [M_1]}$
ϕ_{23}: $m_3 \rightarrow x_2$	$J_{23} = c_{23}[X_2]\frac{[M_3]}{K + [M_3]}$
ϕ_{31}: $m_1 \rightarrow x_3$	$J_{31} = c_{31}[X_3]\frac{[M_1]}{K + [M_1]}$
ϕ_{32}: $m_2 \rightarrow x_3$	$J_{32} = c_{32}[X_3]\frac{[M_2]}{K + [M_2]}$

The modified system ODE is:

$$\frac{d[X_1]}{dt} = J_{1c} - J_4 + J_{12} + J_{13} - D[X_1]$$

$$\frac{d[X_2]}{dt} = J_{2c} - J_5 + J_{21} + J_{23} - D[X_2]$$

$$\frac{d[X_3]}{dt} = J_{3c} - J_6 + J_{31} + J_{32} - D[X_3]$$

$$\frac{d[M_1]}{dt} = J_4 - D[M_1], \qquad \frac{d[M_2]}{dt} = J_5 - D[M_2], \qquad \frac{d[M_3]}{dt} = J_6 - D[M_3]$$

$$\frac{d[S]}{dt} = S_{in} - J_{1c} - J_{2c} - J_{3c} - D[S]$$

$r_{max} = 2$ (1/time)	$K = 0.2$ (biomass/volume)
$K_S = 1$ (biomass/volume)	$a_{ij} = 0 - 0.8$ (volume/biomass)
$S_{in} = 1.5$ (biomass/volume)/time	$c_{ij} = 0 - 0.5$ (1/*time*)
$b_1, b_2, b_3 = 0.4$ (1/*time*)	$D = 1 - 10$ (1/time)

Next, we convert the above chemostat equations into SRN-equivalent equations. We include the "solvent node" w with the solvent concentration $[W]$ and calculate the total volume of the chemostat system:

$$V = \beta_{x1}[X_1] + \beta_{x2}[X_2] + \beta_{x3}[X_3] + \beta_{m1}[M_1] + \beta_{m2}[M_2] + \beta_{m3}[M_3] + \beta_S[S] + \beta_w[W]$$

where $\beta_{(.)} = \frac{\text{unit volume of a cell (or a metabolite)}}{\text{mass per cell (or per metabolite)}}$

Since the chemostat has a constant volume, we have V as a time-independent constant. Now, we can define $(X_1, X_2, X_3, M_1, M_2, M_3, S, W)$ as the total mass of cells or molecules in the chemostat. This gives the relation $X_j = V[X_j]$, $M_j = V[M_j]$ and $S = V[S]$. The SRN-type flux functions are calculated using the following equations:

Reaction	Flux function
ϕ_1: {env}→ x_1	$J_{1c} = \frac{r_{max}(S/V)}{K_S + (S/V)} X_1 \left(1 - \sum_{j=1}^{3} a_{1j}(X_j/V)\right)$
ϕ_2: {env}→ x_2	$J_{2c} = \frac{r_{max}(S/V)}{K_S + (S/V)} X_2 \left(1 - \sum_{j=1}^{3} a_{2j}(X_j/V)\right)$
ϕ_3: {env}→ x_3	$J_{3c} = \frac{r_{max}(S/V)}{K_S + (S/V)} X_3 \left(1 - \sum_{j=1}^{3} a_{3j}(X_j/V)\right)$
ϕ_4: $x_1 \to m_1$	$J_4 = b_1 X_1$
ϕ_5: $x_2 \to m_2$	$J_5 = b_2 X_2$
ϕ_6: $x_3 \to m_3$	$J_6 = b_3 X_3$
ϕ_{12}: $m_2 \to x_1$	$J_{12} = c_{12} X_1 \frac{(M_2/V)}{K + (M_2/V)}$
ϕ_{13}: $m_3 \to x_1$	$J_{13} = c_{13} X_1 \frac{(M_3/V)}{K + (M_3/V)}$
ϕ_{21}: $m_1 \to x_2$	$J_{21} = c_{21} X_2 \frac{(M_1/V)}{K + (M_1/V)}$

$\phi_{23}:\ m_3 \rightarrow x_2$	$J_{23} = c_{23} X_2 \dfrac{(M_3/V)}{K + (M_3/V)}$
$\phi_{31}:\ m_1 \rightarrow x_3$	$J_{31} = c_{31} X_3 \dfrac{(M_1/V)}{K + (M_1/V)}$
$\phi_{32}:\ m_2 \rightarrow x_3$	$J_{32} = c_{32} X_3 \dfrac{(M_2/V)}{K + (M_2/V)}$

The equivalent SRN follows the ODE:

$$\frac{dX_1}{dt} = J_{1c} - J_4 + J_{12} + J_{13} - DX_1$$

$$\frac{dX_2}{dt} = J_{2c} - J_5 + J_{21} + J_{23} - DX_2$$

$$\frac{dX_3}{dt} = J_{3c} - J_6 + J_{31} + J_{32} - DX_3$$

$$\frac{dM_1}{dt} = J_4 - DM_1, \qquad \frac{dM_2}{dt} = J_5 - DM_2, \qquad \frac{dM_3}{dt} = J_6 - DM_3$$

$$\frac{dS}{dt} = S_{in}V - J_{1c} - J_{2c} - J_{3c} - DS$$

$$-\frac{dW}{dt} = \sum_{j=1}^{3} \left(\frac{\beta_{xj}}{\beta_W}\right) \frac{dX_1}{dt} + \sum_{j=1}^{3} \left(\frac{\beta_{mj}}{\beta_W}\right) \frac{dM_1}{dt} + \left(\frac{\beta_S}{\beta_W}\right) \frac{dS}{dt}$$

By choosing the proper volume unit, we can set $V = 1$ in the numerical simulation. In this case, the flux functions of the SRN-equivalent model become identical to those of chemostat model. In our simulation, we choose $V = 1$ liter and measure the total mass of X_j, M_j, S, W in grams. The parameters of the SRN-equivalent model are:

$r_{max} = 2$ (1/time)<br>$K_S = 1$ (g/L)<br>$S_{in} = 1.5$ (g/L)/time<br>$b_1, b_2, b_3 = 0.4$ (1/time)	$K = 0.2$ (g/L)<br>$a_{ij} = 0 - 0.8$ (L/g)<br>$c_{ij} = 0 - 0.5$ (1/time)<br>$D = 1 - 10$ (1/time)

We performed simulations using the above equations and the random coefficients a_{ij} and c_{ij}, where a_{ij} was sampled from a uniform distribution on [0,0.8] and c_{ij} was sampled from a uniform distribution on [0,0.1]. For most random parameters, the model converged to a fixed point. In about 0.2% of the cases the model converged to a heteroclinic cycle, while the limit cycle cases were again rare (3 out of 120,000 random parameter sets).

System parameters of Fig. 4E:

Initial condition: $(X_1, X_2, X_3, M_1, M_2, M_3, S)$ = (0.018, 0.012, 0.038, 0.592, 0.296, 0.044, 0.500)

$a_{ij} = \begin{pmatrix} 0.383 & 0.660 & 0.235 \\ 0.351 & 0.354 & 0.667 \\ 0.665 & 0.024 & 0.270 \end{pmatrix}$	$c_{ij} = \begin{pmatrix} 0 & 0.003 & 0.049 \\ 0.053 & 0 & 0.063 \\ 0.063 & 0.022 & 0 \end{pmatrix}$

<u>(iv) Autocatalytic biosynthesis model (see Fig. 5A and the main text)</u>

(a) Quasi-steady-state formula for composite Michaelis-Menten equations

To construct the autocatalytic biosynthesis network model, we used a class of nonlinear flux functions based on quasi-steady-state Michaelis-Menten models (50). All models contained "catalytic enzymes" and one or more "reactants". In a coarse-grained spirit, we did not include all intermediate metabolites in the model; instead, we used the end product formation flux (the irreversible step in the Michaelis-Menten model) as the flux function. In the following section, we describe the flux functions used in the model. We denote E_{total} as the total amount of enzyme *E,* including all possible enzyme states (free, bound, etc.).

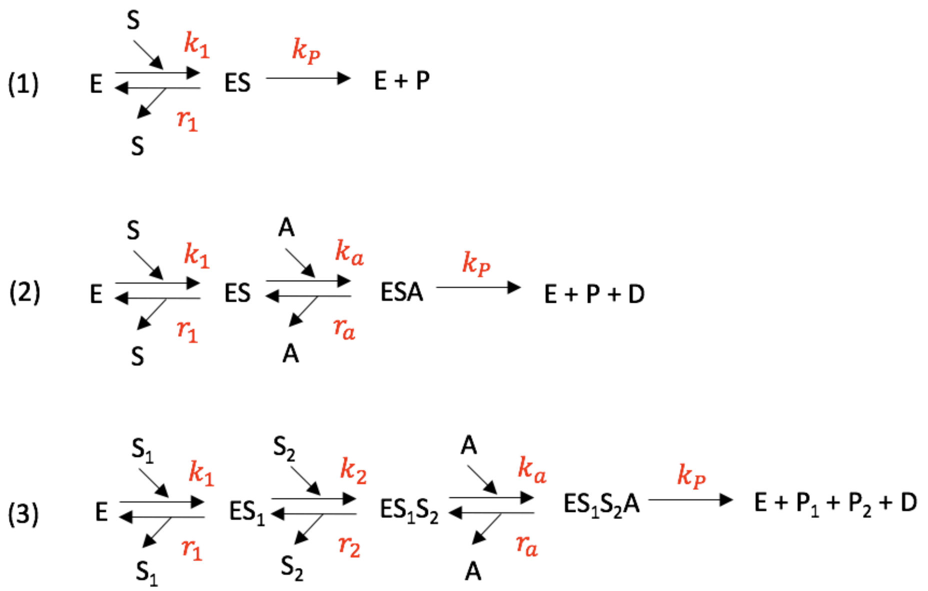

(1) One-substrate Michaelis-Menten equation (assuming quasi-steady state on the [ES] complex): The flux function is

$$J = \frac{k_P[S]}{K_S + [S]} E_{total}$$

with $K_s = \frac{r_1 + k_P}{k_1}$.

(2) Two-substrate Michaelis-Menten equation (assuming detailed balance between reversible reactions and quasi-steady state on the [ESA] complex): The flux function is

$$J = \frac{k_P[S][A]}{\rho_1 K_a + K_a[S] + [S][A]} E_{total}$$

with $\rho_1 = \frac{r_1}{k_1}, K_a = \frac{r_a + k_P}{k_a}$.

(3) Three-substrate Michaelis-Menten equation (assuming detailed balance between reversible reactions and quasi-steady state on the $[ES_1S_2A]$ complex): The flux function is

$$J = \frac{k_P[S_1][S_2][A]}{\rho_1\rho_2 K_a + \rho_2 K_a[S_1] + K_a[S_1][S_2] + [S_1][S_2][A]} E_{total}$$

with $\rho_1 = \frac{r_1}{k_1}, \rho_2 = \frac{r_2}{k_2}, K_a = \frac{r_a + k_P}{k_a}$.

(4) Polymer reaction flux (assuming that the initial complex formation between E and E_o to be at equilibrium and that each polymerization step is a two-substrate Michaelis-Menten equation with similar conditions as in (2), for a total of *n* cycles).

(4)

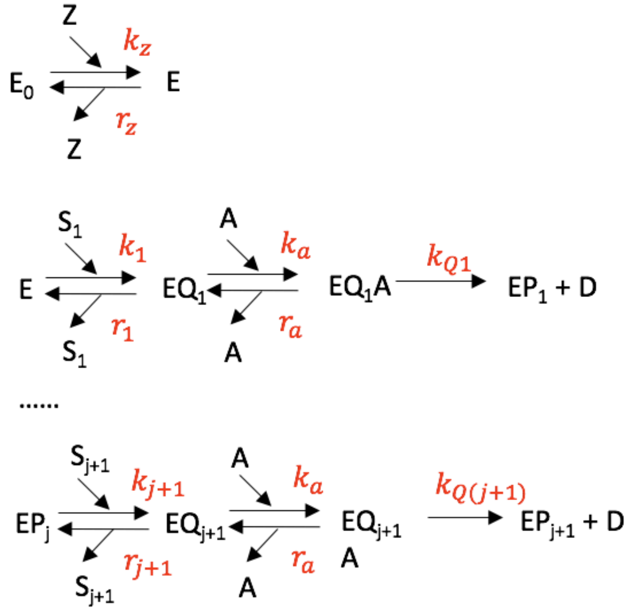

Note that we have the following relation for all *j*=1,…,*n*:

$$k_{Q1}[EQ_1A] = \cdots = k_{Qj}[EQ_jA]$$

$$[EQ_jA] = \frac{[A]}{K_j}[EQ_j] \ = \frac{[A][S_j]}{K_j\rho_j}[EP_j]$$

where $K_j = \frac{k_{Q_j}+r_a}{k_a}$, $\rho_j = r_j/k_j$.

In particular, $[EQ_1A] = \left(\frac{[A]}{K_1}\right)[EQ_1] = \left(\frac{[A][S_1]}{K_j\rho_j}\right)[E]$. In addition, $E_o = \left(\frac{r_z}{k_z[Z]}\right)E \equiv \left(\frac{\rho_Z}{Z}\right)E$.

Therefore, by summing all (bound and unbound) enzymes, we have

$$[E_o] = [E]\left\{\frac{\rho_Z}{[Z]} + \sum_{j=1}^{n}\frac{k_{Q_1}}{k_{Q_j}}\left(1+\frac{K_1}{[A]}+\frac{K_1\rho_1}{[A][S_1]}\right)\left(1+\frac{K_1}{[A]}+\frac{K_1\rho_1}{[A][S_1]}\right)\right\}$$

and hence

$$J = k_{Q_1}[S_1][A][E_o]\left\{\frac{\rho_Z}{[Z]}(K_1\rho_1 + K_1[S_1] + [A][S_1]) + \sum_{j=1}^{n}\frac{k_{Q_1}}{k_{Q_j}}\frac{[S_1]}{[S_j]}(K_j\rho_j + K_j[S_j] + [A][S_j])\right\}^{-1}$$

For a polymer requiring $\{n_1, \dots, n_m\}$ monomers with monomer type 1,…,*m*, the summation can be expressed by

$$J = k_{Q_1}[S_1][A][E_o]\left\{\frac{\rho_Z}{[Z]}(K_1\rho_1 + K_1[S_1] + [A][S_1]) + \sum_{b=1}^{m} n_b\frac{k_{Q_1}}{k_{Q_b}}\frac{[S_1]}{[S_b]}(K_b\rho_b + K_b[S_b] + [A][S_b])\right\}^{-1}$$

(b) Nodes, reactions and flux functions of the autocatalytic biosynthesis model

There are 24 nodes and 40 reactions in our autocatalytic biosynthesis model. All flux functions of the 40 reactions are scalable (see Table S1); their formulas are derived in the previous section. The 24 nodes, including 12 metabolites (red) and 12 polymers (green) are shown in the figure below. The external nutrients s_1, s_2, s_3 belong to the environment and are not part of the system nodes. They are assumed to be maintained at *constant concentration levels* for each simulation condition.

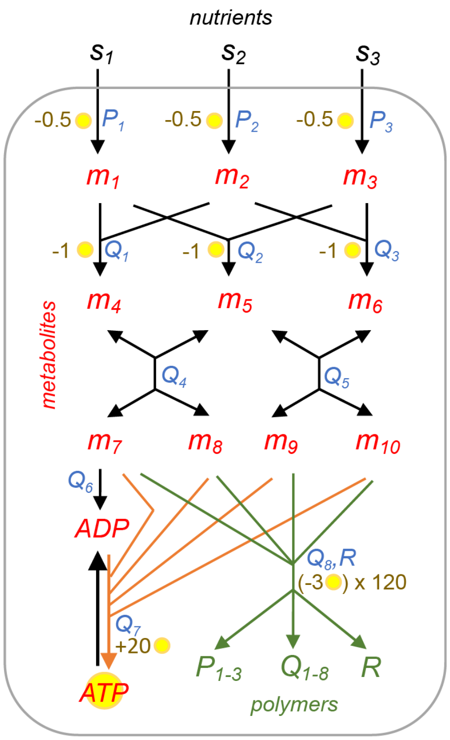

s_1, s_2, s_3: external resources

m_{1-6}: intermediate metabolites
m_{7-10}: amino acids

ATP: free-energy carrying molecule (active)
ADP: free-energy carrying molecule (inactive)

P_{1-3}: transporters of external resources

Q_{1-5}: synthesis enzymes for metabolites
Q_6: *de novo* synthesis enzyme for ATP
Q_7: catabolic enzyme, regenerating ATP by utilizing amino acid m_{7-10}

Q_8: genetic materials (DNA and RNA) required for the synthesis of various enzymes

R: ribosome, anabolic enzyme that synthesizes all polymers, including itself.

The 40 fluxes are indicated by the arrows in the figure. Note that many fluxes are coupled with $\mathrm{ATP} \rightarrow \mathrm{ADP}$ reactions and hence have ATP as an upstream node and ADP as a downstream node. For clarity, all ATP-related arrows are not drawn; instead, the ATP coupling is indicated by the "yellow coin" symbol. The full stoichiometry of the reaction is listed in Table S2.

In addition, many reactions require enzyme catalysis, indicated by blue letters next to the reactions. These enzymes are required for flux, but are not consumed by the reaction. Hence, they do not belong to the upstream or downstream nodes of these reactions. The dependence of these enzymes is shown by the flux function in Table S2.

(c) Parameter range of the autocatalytic biosynthesis model

We tested a wide range of parameter values and various initial conditions for the system. Most of the trajectories $Y(t)$ converged to a fixed point or limit cycle, resulting in convergence of the long-term growth rate. We chose a "standard parameter set" (see Table S3) in which the system trajectory $Y(t)$ converges to a steady state. Due to the simplicity of our toy model, our parameter set cannot be directly compared to realistic metabolite concentrations. However, we can compare the relative fraction of biomass of each polymer and metabolite between simulations and the cell. Note that all parameters follow the unit system of biomass vector $X(t)$, which is unitless.

To compare with classical biochemical rate constants, one can use Eq. (5) presented in the main text. We found that under our standard parameter set (Table S3), the 12 metabolites in our toy

model account for 4.3% of biomass, while the 12 polymers account for more than 95% of the biomass. Among the polymers, the ribosomes (Y_R) and the genetic material (Y_{Q_8}) account for ~33% and ~8% of biomass, respectively (see Table S3).

This biomass partition is physiologically realistic for *Escherichia coli*. In this bacterium, macromolecules account for ~95% of the biomass [Ref(51)], with ribosomes occupying 20% to 40% of the biomass of the polymer fraction [Ref(51)]. Polymers that are not ribosomal RNAs (rRNAs) (including mRNA, tRNA and DNA) account for ~7% of the biomass [Ref(52)]. In *E. coli*, the ATP concentration is about 2 mM [Ref(53)], with a molecular weight (MW) = 507 $g/mole$. Suppose the cell is 30% dry weight (i.e., biomass) and the dry weight density is about 1.1 g/cm^3[Ref(54)], the biomass fraction of ATP is about 0.33%. In our model we have Y_{ATP}= 0.45% which is closed to the physiological range. For the energy balance level, the ratio ATP:ADP is about 5-10 folds [Ref(55)]. In our model, Y_{ATP}: Y_{ADP} ~ 4, which is close to the physiological range.

5. Analysis of Random networks and autocatalytic circuits

(i) Generation of random networks

In the analysis of symbolic reaction networks, an ensemble of random networks is generated according to a given statistical property of the network connections. Each random network is composed of a node set x and a reaction set ϕ. Each reaction ϕ_a has its associated maintenance set $mt(\phi_a) \subseteq x$ and downstream set $dw(\phi_a) \subseteq x$. We simulated the network as follows:

(1) First, we fixed the numbers of nodes and reactions to be *n* and *m* and set two constants n_{mt}, n_{dw} as the numbers of maintenance nodes and downstream nodes for all reactions. For Fig. S6, we chose $n_{mt} = 3$ and $n_{mt} = 2$.

(2) For each node x_j, we assigned a weighting factor W_j^{mt} to the node. This weighting factor determines the relative likelihood that node x_j is chosen as the maintenance set of a random reaction. The weighting factors are chosen over the interval $(0, w_{max}]$, with a probability distribution

$$\Pr\{W_j^{mt} \in [w, w + dw]\} = Cw^{-\gamma_{mt}}dw$$

with the normalizing constant $C \equiv \int_0^{w_{max}} w^{\gamma_{mt}} dw$.

The aforementioned distribution follows a power law with exponent $-\gamma_{mt}$ and is truncated in the range $(0, w_{max}]$. In simulations, γ_{mt} varied between 0 and 3 and $w_{max} = \gamma_{mt}|\log(10^{-4})| \approx 9\,\gamma_{mt}$. This was sufficient to generate a wide range of values for W_j^{mt} with several orders of magnitude with probability of $\Pr\{W_j^{mt}\}$.

(3) After each node was assigned a weighting factor W_j^{mt}, we normalized the weighting factor and acquired a probability function $P_{mt}(j)$ by

$$P_{mt}(j) = \frac{W_j^{mt}}{\sum_{j=1}^{n} W_j^{mt}}$$

For each reaction and each slot of a maintenance set, a random node was drawn from $\boldsymbol{x}$ according to probability $P_{mt}(j)$ and assigned to the maintenance node slot. This specified the maintenance set $mt(\phi_a)$ for each reaction ϕ_a.

(4) For each node x_j, a similar method is used to generate the node set $dw(\phi_a)$ for each reaction ϕ_a. We defined W_j^{dw} with γ_{dw} ranging from 0 to 3, similar to W_j^{mt}. We define $P_{dw}(j) = \frac{W_j^{dw}}{\sum_{j=1}^{n} W_j^{dw}}$. For each reaction and each slot of its downstream set, a random node was drawn from x according to probability $P_{dw}(j)$ and assigned to the downstream node slot. This specified the downstream node $dw(\phi_a)$ for each reaction ϕ_a.

(ii) Identification of autocatalytic circuits in random networks

To test whether a random network has an autocatalytic circuit, we used the following procedure (for the proof of this algorithm, see the Supplementary Text section in the SI, Algorithm 6.3).

(1) Given a collection of reactions $K \subseteq \phi$, we defined

$$mt(K) \equiv \bigcup_{\phi_a \in K} mt(\phi_a)$$

$$dw(K) \equiv \bigcup_{\phi_a \in K} dw(\phi_a)$$

(2) We started with a collection of reactions $K_0 \equiv \phi$. For each $m \geq 0$, we checked if $mt(K_m) \subseteq dw(K_m)$. If true, K_m is an autocatalytic circuit and the network is autocatalytic. If false, we scanned for all reactions and obtained a subset A_m: $\{\ \phi_a \in K_m,\ mt(\phi_a) \notin K_m\}$. We deleted this reaction subset from K_m and obtained $K_{m+1} \equiv K_m \backslash A_m$.

(3) The procedure above was repeated until either K_m became autocatalytic or an empty set. If K_m became an empty set, then the network contained no autocatalytic circuit.

(iii) Calculation of the autocatalytic probability for random networks

For Fig. S6, random reaction networks with 100 nodes and various number of reactions were constructed. Each reaction was randomly assigned with three maintenance nodes and two

downstream nodes (using sampling with replacement). The probability of assigning a node to a maintenance or downstream set followed a power law exponent γ_{mt} and γ_{dw}.

For each condition, 500 random networks were generated and tested for their autocatalytic ability using the algorithm shown in the previous section. The autocatalytic probability P_{auto} was calculated as follows:

$$P_{auto} = \frac{\#\ of\ random\ networks\ being\ autocatalytic}{\#\ of\ random\ networks\ simulated}.$$

Supplementary Figures

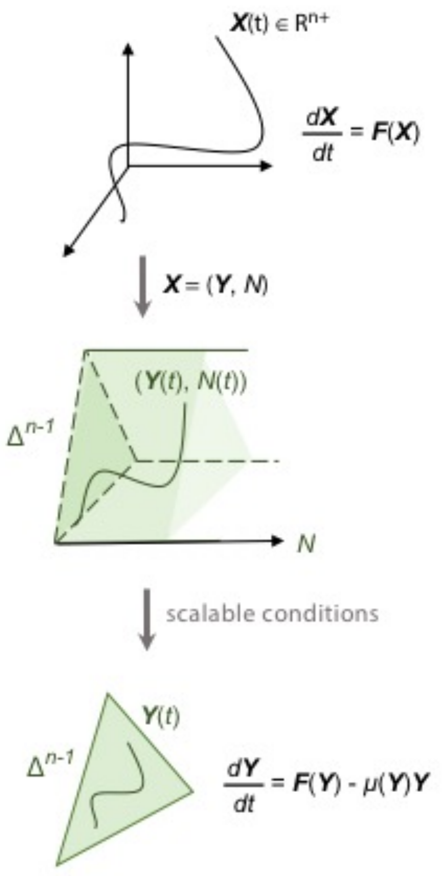

Figure S1: Transformation of a scalable reaction network. Top: System trajectory $X(t)$ in non-negative quadrant Q^n: $\{X \in \mathbb{R}^n,\ X_j \geq 0\}$. Middle: System trajectory $(Y(t), N(t))$ after transformation from X to (Y, N) coordinate. The state space is $\Delta^{n-1} \times [0, \infty)$, where Δ^{n-1}:$\{X \in \mathbb{R}^{n+},\ X_1 + \cdots + X_n = 1\}$ is the (n-1)-dimensional unit simplex. Bottom: For a scalable reaction network, the trajectory Y(t) is confined in Δ^{n-1} and the system dynamics is fully determined by Eq. (3) in the main text.

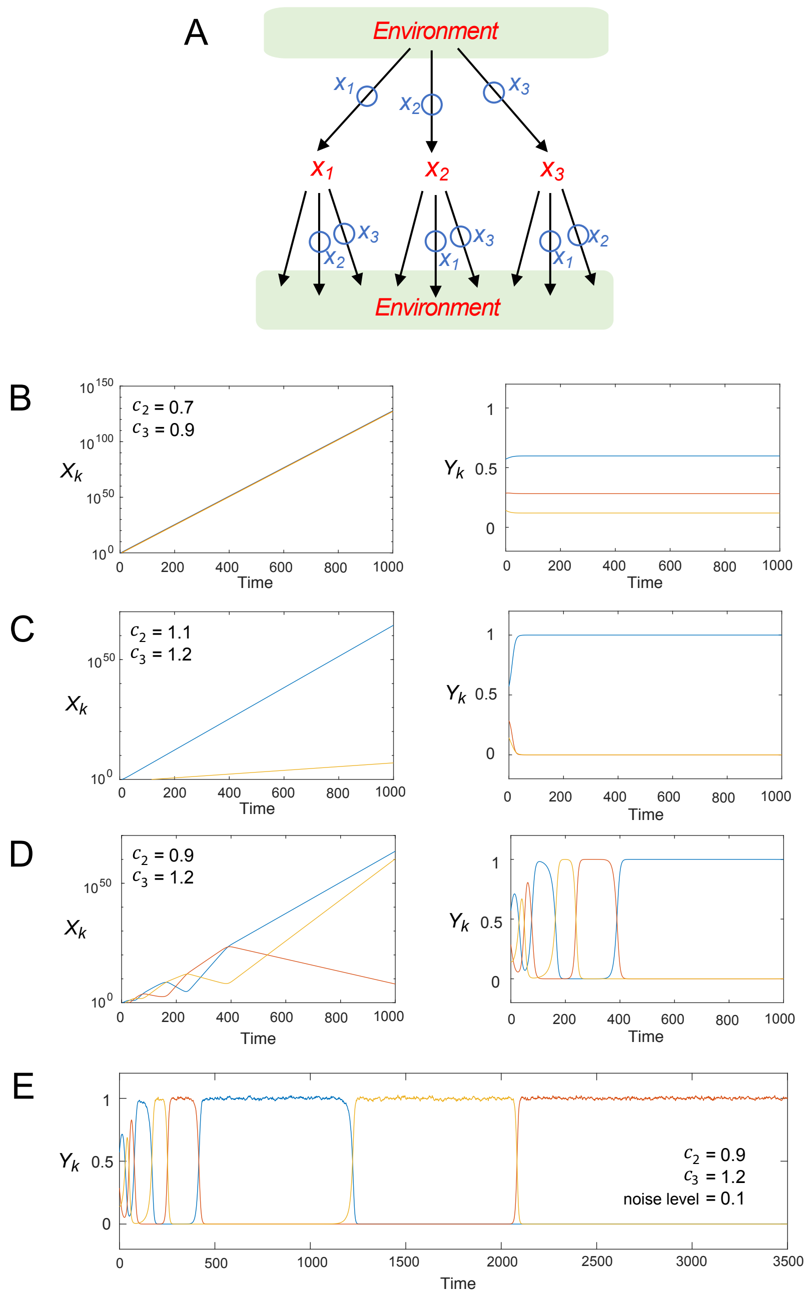

Figure S2: Modified May-Leonard system. **A**, Flux diagram of the system. Blue circles on the flux reactions indicate the nodes that promote the corresponding reaction. For the nonlinear flux function, see example 2.5 in the Supplementary Materials. **B**, System trajectories with $c_2 = 0.7, c_3 = 0.9$. The blue, orange and yellow lines represent X_1, X_2 and X_3 in the left panel, while representing Y_1, Y_2 and Y_3 in the right panel. In the long-term, $Y(t)$ converges to a steady-state, with the three species coexisting. **C**, Same as **B** except that $c_2 = 1.1, c_3 = 1.2$. In the long-term, $Y(t)$ converges to a steady-state, with a single species dominating. **D**, Same as **B** except that $c_2 = 0.9, c_3 = 1.2$. In the long-term, $Y(t)$ converges to a heteroclinic cycle and exhibits oscillations with increasing period length. **E**, Same as **D** except that a scalable noise term $h \circ dB_k$ is added for k=1,2,3 with noise level $h = 0.01$. This shows that the heteroclinic cycle (in **C**) is robust under noise perturbation.

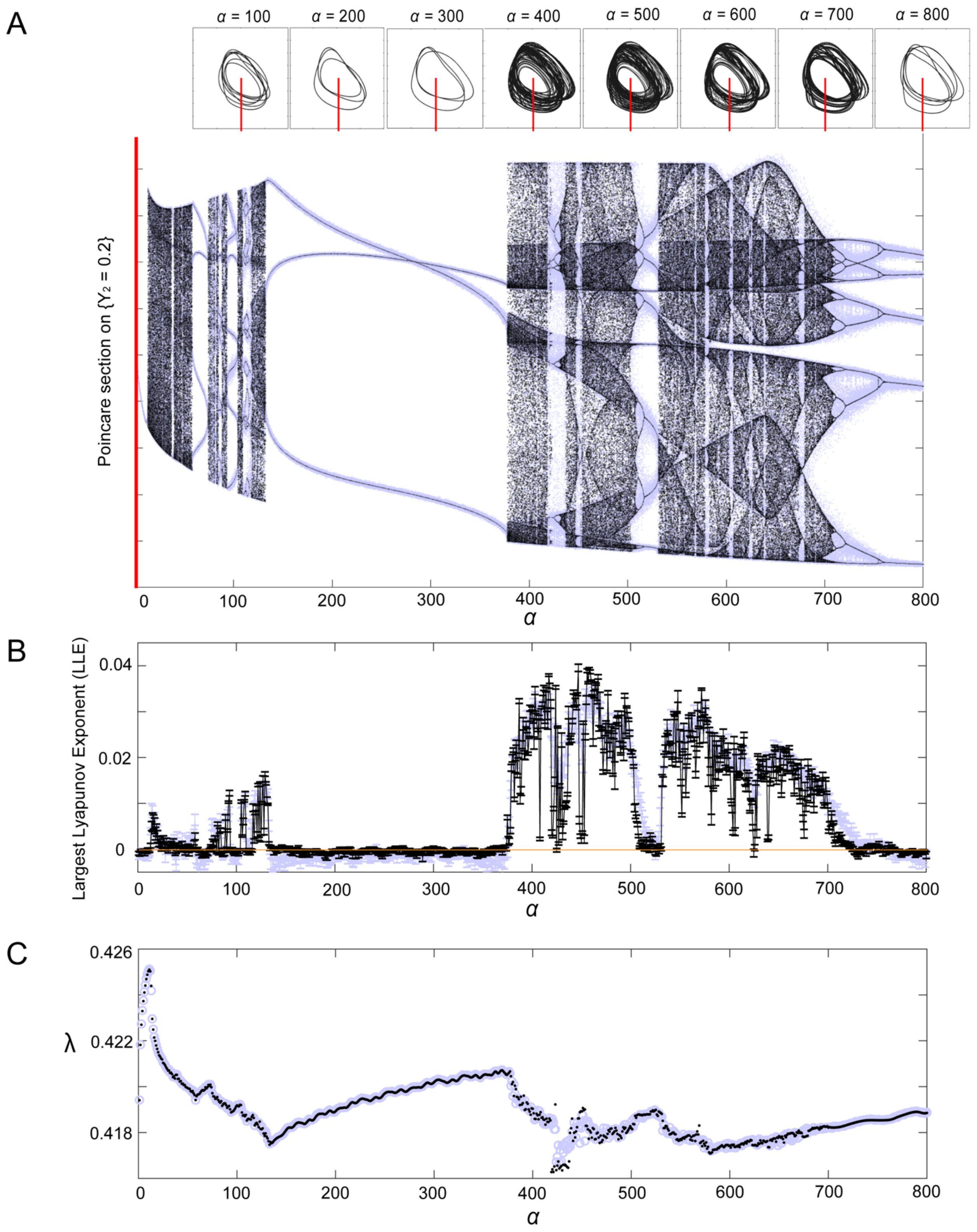

Figure S3: Characterization of the long-term behavior of the double-repressilator model. The analysis is focused on varying parameter $\alpha = K_a/K_b$, the relative repression strength between the two repressilators.

For simulation parameters, see “Methods: Simulation and analysis of scalable networks” in the SI. **A**, Bifurcation diagram of the Poincare section on omega-limit set of $Y(t)$, with various values of α. Upper panel: Trajectory $Y(t)$ projected on $Y_2 - Y_3$ subspace, indicating various types of omega-limit sets of $Y(t)$. Red lines indicate the Poincare section used in the lower panel. Lower panel: Bifurcation diagram on the Poincare section $\{Y_2 = 0.2\}$ (indicated in the top panel). Black dots show simulation results from a deterministic system, while blue dots are the simulation results from the same system, but with small scalable noise (see SI, Supplementary Text, section 4). **B**, Largest Lyapunov exponent (LLE) of $Y(t)$ as a function of α. Each data point is the average of ten simulation results. The error bars indicate the standard error of the simulated LLE. The orange horizontal line indicates LLE = 0. The regime with positive LLE corresponds to a chaotic regime, which can be compared to **A**. The simulation results from a deterministic system are shown in black, while the ones with small scalable noise are shown in blue. **C**, Long-term growth rate as a function of α. The simulation results from a deterministic system are shown in black, while the ones with small scalable noise are in blue.

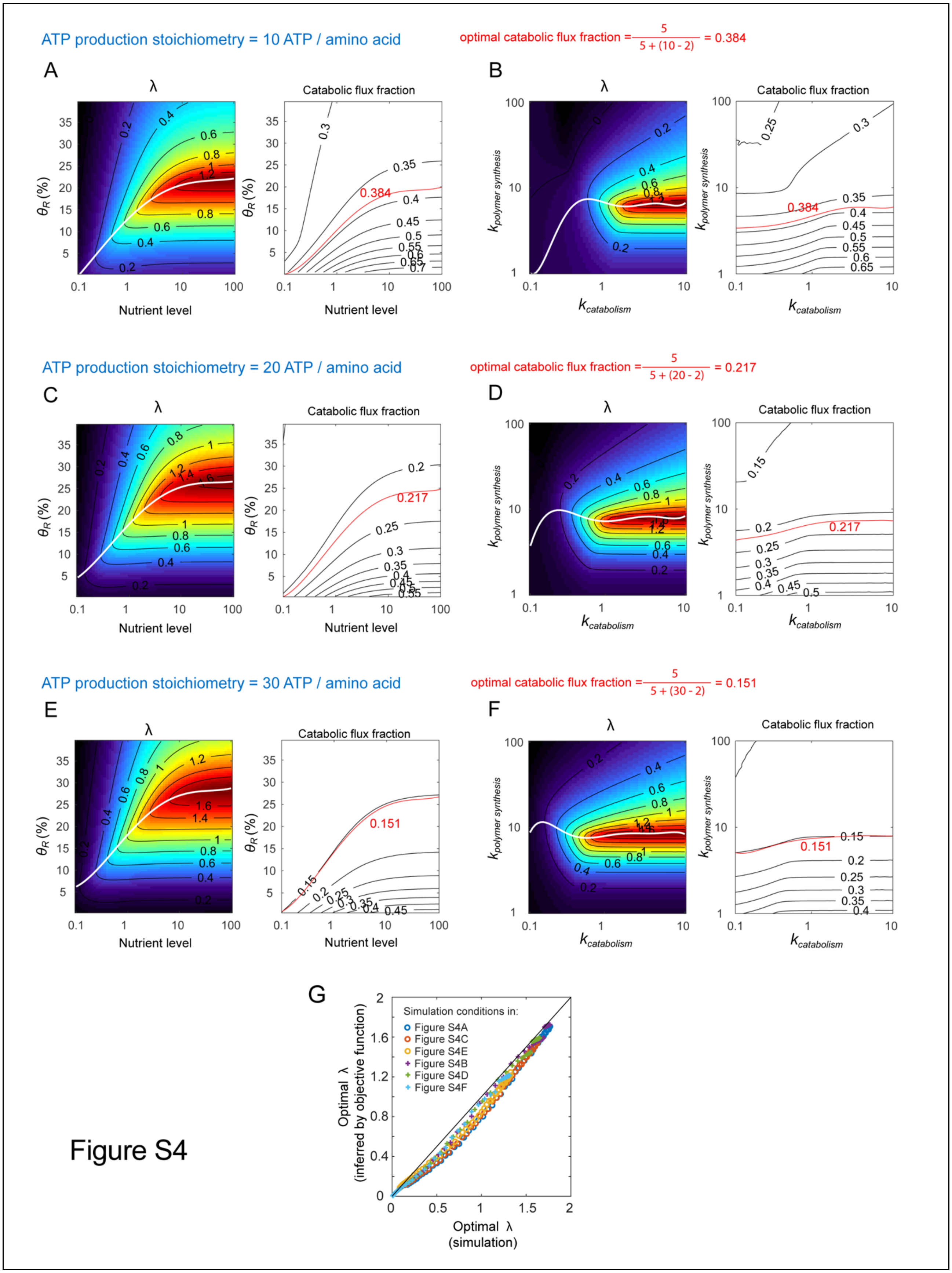
ATP production stoichiometry = 10 ATP / amino acid
optimal catabolic flux fraction = 5 / (5 + (10 - 2)) = 0.384
A
B
λ
Catabolic flux fraction
θ_R (%)
Nutrient level
$k_{polymer\ synthesis}$
$k_{catabolism}$
ATP production stoichiometry = 20 ATP / amino acid
optimal catabolic flux fraction = 5 / (5 + (20 - 2)) = 0.217
C
D
ATP production stoichiometry = 30 ATP / amino acid
optimal catabolic flux fraction = 5 / (5 + (30 - 2)) = 0.151
E
F
G
Simulation conditions in:
Figure S4A
Figure S4C
Figure S4E
Figure S4B
Figure S4D
Figure S4F
Optimal λ
(inferred by objective function)
Optimal λ
(simulation)

Figure S4

Figure S4: Growth rate optimization under different ATP production stoichiometries**. A**, Growth rate and catabolic flux fraction, with the ATP production stoichiometry set to 10. Left: Growth rate with various θ_R (ribosomal synthesis strength) under various external nutrient levels. The white line indicates the maximal growth rate under each nutrient level. Right: Catabolic flux fraction with various θ_R under various external nutrient levels. The red line indicates the contour of the optimal catabolic flux fraction. **B**, Growth rate and catabolic flux fraction, with the ATP production stoichiometry set to 10. Left: Growth rate with various $k_{polymer\ synthesis}$ (polymer synthesis rate constant) under various $k_{catabolism}$ (catabolic rate constant). The white line indicates the maximal growth rate under each value of $k_{catabolism}$. Right: catabolic flux fraction with various $k_{polymer\ synthesis}$ under various $k_{catabolism}$. The red line indicates the contour of the optimal catabolic flux fraction. **C**, Same as **A**, except that the ATP production stoichiometry is set to 20. **D**, Same as **B**, except that the ATP production stoichiometry is set to 20. **E**, Same as **A**, except that the ATP production stoichiometry is set to 30. **F**, Same as **B**, except that the ATP production stoichiometry is set to 30. **G**, Comparison between the optimal growth rate obtained by simulation and the optimal growth rate inferred from the objective function. This plot contains six different types of optimization conditions in which ATP production stoichiometry is 30, 20 and 10 per amino acid (m_{7-10}), respectively.

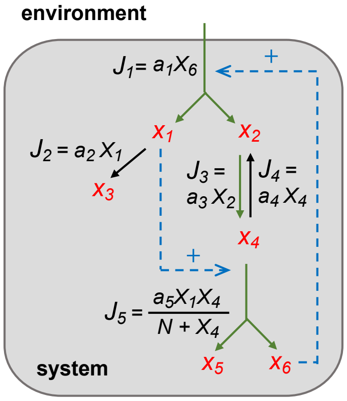

$mt(\varphi_1) = \{x_6\}$ $dw(\varphi_1) = \{x_1, x_2\}$

$mt(\varphi_2) = \{x_1\}$ $dw(\varphi_2) = \{x_3\}$

$mt(\varphi_3) = \{x_2\}$ $dw(\varphi_3) = \{x_4\}$

$mt(\varphi_4) = \{x_4\}$ $dw(\varphi_4) = \{x_2\}$

$mt(\varphi_5) = \{x_1, x_4\}$ $dw(\varphi_5) = \{x_5, x_6\}$

$mt(\{\varphi_1, \varphi_3, \varphi_5\}) = \{x_1, x_2, x_4, x_6\} \subseteq dw(\{\varphi_1, \varphi_3, \varphi_5\})$

Fig. S5: Example of an autocatalytic circuit in a scalable reaction network. In this system, there are five reactions with associated flux function forms specified in the figure. The maintenance and downstream sets of reactions are listed on the right. A subset of reactions $K \equiv \{\phi_1, \phi_3, \phi_5\}$ satisfies $mt(K) \subseteq dw(K)$, indicating that this reaction network is autocatalytic.

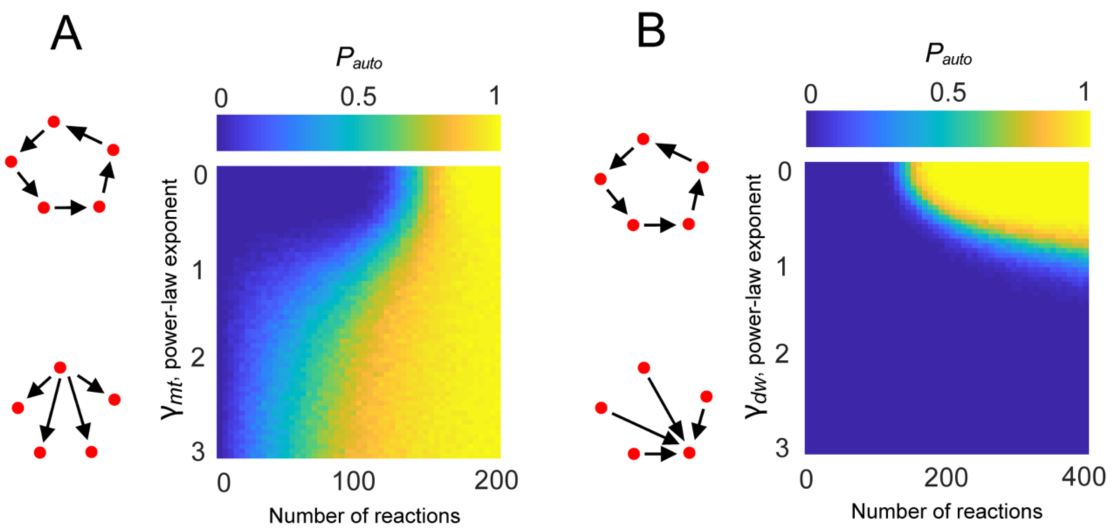

Fig. S6: Phase diagram of P_{auto}, the probability of a random network to be autocatalytic. Ensembles of random networks were simulated under different power-law connection probabilities and various numbers of reactions. All random reaction networks have 100 nodes and each reaction has three maintenance nodes and two downstream nodes. **A**, Network with power-law exponent $\gamma_{dw} = 0$ and γ_{mt} varying from 0 (uniform connection) to 3 (hub-like maintenance nodes). **B**, Network with power-law exponent $\gamma_{dw} = 0$ and γ_{dw} varying from 0 (uniform connection) to 3 (hub-like downstream nodes).

Supplementary Tables

$g_1(u_1, u_2; c_1, c_2) = \frac{c_1 u_1 u_2}{u_2 + c_2 N}$
$g_2(u_1, u_2, u_3; c_1, c_2, c_3) = \frac{c_1 u_1 u_2 u_3}{c_2 c_3 N^2 + c_2 N u_2 + u_2 u_3}$
$g_3(u_1, u_2, u_3, u_4; c_1, c_2, c_3, c_4) = \frac{c_1 u_1 u_2 u_3 u_4}{c_2 c_3 c_4 N^3 + c_2 c_4 N^2 u_2 + c_2 N u_2 u_3 + u_2 u_3 u_4}$
$g_{poly}(u_1, u_2, u_3, u_4, u_5, u_6, u_7; c_1, c_2, c_3, c_4, c_5, c_6, c_7, c_8) = c_2 u_1 u_2 u_4 \times \left\{ \frac{c_1}{u_3}(c_3 c_4 N^2 + c_3 N u_4 + u_2 u_4) + \sum_{b=1}^{4} \frac{c_{b+4} u_4}{u_{b+3}}(c_3 c_4 N^2 + c_3 N u_{b+3} + u_2 u_{b+3}) \right\}^{-1}$
$g_5(u_1; c_1) = c_1 u_1$

Table S1: Common flux functions.
This table lists the scalable flux functions used in the biosynthesis model. Here, u_j are variables of the scalable flux function and we denote $N = u_1 + \cdots + u_n$. The parameters in the function are denoted as c_j. The formulas are derived from Michaelis-Menten kinetics on quasi-steady-state reactions (see Method section: Mathematical models of SRN iv (a)), assuming that the cell volume is proportional to the total biomass N during the system growth.

Reactions	Flux functions	Notes
$\phi_1: s_1 + ATP \rightarrow m_1 + ADP$	$g_1\left(X_{P_1}, X_{ATP}; r_{import,1}(s_1), K_A\right)$	1
$\phi_2: s_2 + ATP \rightarrow m_2 + ADP$	$g_1\left(X_{P_2}, X_{ATP}; r_{import,2}(s_2), K_A\right)$	
$\phi_3: s_3 + ATP \rightarrow m_3 + ADP$	$g_1\left(X_{P_3}, X_{ATP}; r_{import,3}(s_3), K_A\right)$	
$\phi_4: 0.5m_1 + 0.5m_2 + ATP \rightarrow m_4 + ADP$	$g_3\left(X_{Q_1}, X_{m_1}, X_{m_2}, X_{ATP}; r_S, K_{AS}, \rho_{S1}, \rho_{S2}\right)$	
$\phi_5: 0.5m_1 + 0.5m_3 + ATP \rightarrow m_5 + ADP$	$g_3\left(X_{Q_2}, X_{m_1}, X_{m_3}, X_{ATP}; r_S, K_{AS}, \rho_{S1}, \rho_{S2}\right)$	
$\phi_6: 0.5m_2 + 0.5m_3 + ATP \rightarrow m_6 + ADP$	$g_3\left(X_{Q_3}, X_{m_2}, X_{m_3}, X_{ATP}; r_S, K_{AS}, \rho_{S1}, \rho_{S2}\right)$	
$\phi_7: m_4 + m_5 \rightarrow m_7 + m_8$	$g_3\left(X_{Q_4}, X_{m_4}, X_{m_5}, X_{ATP}; r_F, \rho_{F1}, \rho_{F2}, K_{AF}\right)$	2
$\phi_8: m_5 + m_6 \rightarrow m_9 + m_{10}$	$g_3\left(X_{Q_5}, X_{m_5}, X_{m_6}, X_{ATP}; r_F, \rho_{F1}, \rho_{F2}, K_{AF}\right)$	
$\phi_9: m_7 + m_8 \rightarrow m_4 + m_5$	$g_2\left(X_{Q_4}, X_{m_7}, X_{m_8}; r_R, K_R, \rho_R\right)$	
$\phi_{10}: m_9 + m_{10} \rightarrow m_5 + m_6$	$g_2\left(X_{Q_5}, X_{m_9}, X_{m_{10}}; r_R, K_R, \rho_R\right)$	
$\phi_{11}: m_7 \rightarrow ADP$	$g_1\left(X_{Q_6}, X_{m_7}; r_{ADPdenovo}, K_{ADPdenovo}\right)$	3
$\phi_{12}: m_7 + n_{cat\_value}ADP \rightarrow n_{cat\_value}ATP$	$g_2\left(X_{Q_7}, X_{m_7}, X_{ATP}; k_{catabolism}, K_{cat}, \rho_C\right)$	
$\phi_{13}: m_8 + n_{cat\_value}ADP \rightarrow n_{cat\_value}ATP$	$g_2\left(X_{Q_7}, X_{m_8}, X_{ATP}; k_{catabolism}, K_{cat}, \rho_C\right)$	
$\phi_{14}: m_9 + n_{cat\_value}ADP \rightarrow n_{cat\_value}ATP$	$g_2\left(X_{Q_7}, X_{m_9}, X_{ATP}; k_{catabolism}, K_{cat}, \rho_C\right)$	
$\phi_{15}: m_{10} + n_{cat\_value}ADP \rightarrow n_{cat\_value}ATP$	$g_2\left(X_{Q_7}, X_{m_{10}}, X_{ATP}; k_{catabolism}, K_{cat}, \rho_C\right)$	
$\phi_{16}: n_{m_7}m_7 + n_{m_8}m_8 + n_{m_9}m_9 + n_{m_{10}}m_{10}$<br>$+n_{syn\_value}\left(n_{m_7} + n_{m_8} + n_{m_9} + n_{m_{10}}\right) ATP$<br>$\rightarrow \sum_{j=1}^{3} \theta_{P_j}P_j + \sum_{j=1}^{8} \theta_{Q_j}Q_j + \theta_R R$<br>$+n_{syn\_value}\left(n_{m_7} + n_{m_8} + n_{m_9} + n_{m_{10}}\right) ADP$	$g_{poly}(X_R, X_{ATP}, X_{Q_8}, X_{m_7}, X_{m_8}, X_{m_9}, X_{m_{10}};$<br>$K_{Q8,poly}, k_{polymer\ synthesis}, K_{a,poly}, \rho_{poly},$<br>$n_{m_7}, n_{m_8}, n_{m_9}, n_{m_{10}})$	4
$\phi_{17-19}: P_{1-3} \rightarrow\ n_{m_7} + n_{m_8} + n_{m_9} + n_{m_{10}}$<br>$\phi_{20-26}: Q_{1-5} \rightarrow\ n_{m_7} + n_{m_8} + n_{m_9} + n_{m_{10}}$<br>$\phi_{27}: \quad R \rightarrow\ n_{m_7} + n_{m_8} + n_{m_9} + n_{m_{10}}$<br>$\phi_{28}: \quad Q_8 \rightarrow\ n_{m_7} + n_{m_8} + n_{m_9}n_{m_{10}}$	$g_1\left(X_{P_{1-3}}; d_{P_{1-3}}\right)$<br>$g_1\left(X_{Q_{1-7}}; d_{Q_{1-7}}\right)$<br>$g_1(X_R; d_R)$<br>$g_1\left(X_{Q_8}; d_{Q_8}\right)$	5
$\phi_{29-38}: m_{1-10} \rightarrow \{\emptyset\}$<br>$\phi_{39}: ATP \rightarrow \{\emptyset\}$<br>$\phi_{40}: ADP \rightarrow \{\emptyset\}$	$g_1\left(X_{m_{1-10}}; d_{m_{1-10}}\right)$<br>$g_1(X_{ATP}; d_{ATP})$<br>$g_1(X_{ADP}; d_{ADP})$	6

Table S2: Reactions and flux functions in the autocatalytic biosynthesis model. This table lists 24 reactions in the autocatalytic biosynthesis network, with the corresponding flux function. The mathematical formulas of these flux functions (g_1, g_2, g_3, g_{poly} and g_5) are described in Table S1. Here, the subscript of the variable $X_{(.)}$ denotes the name of the corresponding node.

Note 1: The external nutrient level s_j affects the parameter $r_{import,j}$, with the Michaelis-Menten form $r_{import,j} = \frac{r_{s_j} s_j}{K_{s_j} + s_j}$ for *j* = 1,2,3.

Note 2: The reactions ϕ_7 and ϕ_8 do not consume ATP, but require ATP as an intermediate in the flux function (i.e., ATP acts as a cofactor for the enzymes Q_4 and Q_5).

Note 3: Reaction ϕ_{11} is the *de novo* synthesis of ADP. This step does not involve ATP.

Note 4: Reaction ϕ_{16} describes the process of overall polymer synthesis. It uses amino acids m_{7-10} and ATP energy, and generates 12 types of polymers with stoichiometry parameters $\{\theta_{P_{1-3}}, \theta_{Q_{1-8}}, \theta_R\}$. By incorporating the stoichiometry coefficients into the common kinetic parameter $k_{polymer\ synthesis}$, the coefficient θ_α can be normalized such that $\sum_\alpha \theta_\alpha$ = 1. Hence, θ_α represents the fraction of polymer synthesis flux. Biologically, θ_α can be regarded as the (relative) polymer synthesis strength, which is the fraction of the total capacity of polymer synthesis occupied by the polymer type α. In the main text, the ribosomal synthesis strength θ_R is set to different values to vary the ribosomal fraction and change the polymer partition.

Note 5: Reactions ϕ_{17-28} represent first-order polymer degradation fluxes. The polymer is degraded to regenerate amino acids m_{7-10}.These reactions are not coupled with ATP consumption.

Note 6: Reaction ϕ_{29-40} represent first-order monomer degradation fluxes. These reactions do not have downstream nodes and can be regarded as degradation of metabolites.

Parameter	Value used in the standard parameter set	Reaction involved	Parameter regime tested
$r_{import,1-3}$	$r_{import,j} = \frac{r_{s_j} s_j}{K_{s_j}+s_j} =$ 54.5 (1/t)<br>$s_1 = s_2 = s_3$ =10 (mM)<br>r_{s_j} = 60 (1/t)<br>K_{s_j} =1 (mM)	ϕ_{1-3}	s_j =0.01 mM – 100 mM
K_A	0.005	ϕ_{1-3}	
r_S	120 (1/t)	ϕ_{4-6}	
K_{AS}	0.005	ϕ_{4-6}	
ρ_{S1}	0.01	ϕ_{4-6}	
ρ_{S2}	0.01	ϕ_{4-6}	
r_F	250 (1/t)	ϕ_{7-8}	
ρ_{F1}	0.005	ϕ_{7-8}	
ρ_{F2}	0.005	ϕ_{7-8}	
K_{AF}	0.005	ϕ_{7-8}	
r_R	1 (1/t)	ϕ_{9-10}	
K_R	0.04	ϕ_{9-10}	
ρ_R	1	ϕ_{9-10}	
$r_{ADPdenovo}$	1	ϕ_{11}	
$K_{ADPdenovo}$	0.05	ϕ_{11}	
$k_{catabolism}$	6 (1/t)	ϕ_{12-15}	0.1-10 (1/t)
K_{cat}	0.001 (Fig. 3B, Table S4)<br>0.005 (Fig. 3F, Fig. S4)	ϕ_{12-15}	
ρ_C	0.05	ϕ_{12-15}	
n_{cat_value}	20	ϕ_{12-15}	10 - 30
n_{syn_value}	3	ϕ_{16}	
$K_{Q8,poly}$	0.002	ϕ_{16}	
$k_{polymer\ synthesis}$	10	ϕ_{16}	1 - 100
$K_{a,poly}$	0.0025	ϕ_{16}	
ρ_{poly}	0.005	ϕ_{16}	
$n_{m7} - n_{m10}$	30	ϕ_{16-28}	
θ_α	$\theta_{P_{1-3}}$ = 2.0%, $\theta_{Q_{1-5}}$ = 5.2%<br>θ_{Q_6} = 6.9%, θ_{Q_7} = 17.3%<br>θ_{Q_8} = 8.7%, θ_R = 35%	ϕ_{16}	θ_R = 0% - 40%
d_α (polymer)	0.005	ϕ_{17-28}	
d_β (monomer)	0.005	ϕ_{29-40}	

Table S3: Parameters used in the autocatalytic biosynthesis model.
This table shows the parameters used in the simulations. Parameters that were varied in this study are shown in blue.

Node	% of biomass	Node	% of biomass
Y_{m_1}	0.3913	Y_{P_1}	1.9136
Y_{m_2}	0.3913	Y_{P_2}	1.9136
Y_{m_3}	0.3913	Y_{P_3}	1.9136
Y_{m_4}	0.1393	Y_{Q_1}	4.9809
Y_{m_5}	0.1393	Y_{Q_2}	4.9809
Y_{m_6}	0.1393	Y_{Q_3}	4.9809
Y_{m_7}	0.5128	Y_{Q_4}	4.9809
Y_{m_8}	0.5517	Y_{Q_5}	4.9809
Y_{m_9}	0.5517	Y_{Q_6}	6.6411
$Y_{m_{10}}$	0.5517	Y_{Q_7}	16.6028
Y_{ATP}	0.4510	Y_{Q_8}	8.3015
Y_{ADP}	0.1113	Y_R	33.4871

Table S4: Steady-state Y^* of the standard parameter set.
This table lists the steady-state biomass fraction of the autocatalytic biosynthesis model, simulated by the standard parameter set (see Table S3). At this steady state, $\lambda = 1.093$ (1/t).

References

43. I. P. Cornfeld, S. V. Fomin, Y. G. Sinai, *Ergodic Theory* (Springer, 1980).
44. M. B. Elowitz, S. Leibler, A synthetic oscillatory network of transcriptional regulators. *Nature* **403**, 335-338 (2000).
45. R. M. May, W. J. Leonard, Nonlinear aspects of competition between three species. *SIAM J. Appl. Math* **29**, 243 (1975).
46. N. S. Panikov, *Microbial Growth Kinetics* (Chapman and Hall, 1995).
47. B. K. Øksendal, *Stochastic differential equations : an introduction with applications*, Universitext (Springer, Berlin ; New York, ed. 6th, 2007), pp. xxix, 369 p.
48. A. Friedman, M. A. Pinsky, Asymptotic stability and spiraling properties for solution of stochastic equations. *Trans. Amer. Math. Soc* **186**, 331 (1973).
49. A. Friedman, *Stochastic differential equations and applications*, Probability and mathematical statistics series v 28 (Academic Press, New York, 1975).
50. D. A. Beard, H. Qian, *Chemical Biophysics: Quantitative Analysis of Cellular Systems* (Cambridge Unversity Press, 2008).
51. F. C. Neidhardt, J. L. Ingraham, M. Schaechter, *Physiology of the Bacterial Cell: a Molecular Approach* (Sinauer, 1990).
52. H. Bremer, P. Dennis, *Modulation of Chemical Composition and Other Parameters of the Cell at Different Exponential Growth Rates* (Ecosal Plus, 2008), vol. 3.
53. H. Yaginuma *et al.*, Diversity in ATP concentrations in a single bacterial cell population revealed by quantitative single-cell imaging. *Sci Rep* **4**, 6522 (2014).

54. G. Bratbak, I. Dundas, Bacterial dry matter content and biomass estimations. *Appl Environ Microbiol* **48**, 755-757 (1984).
55. M. H. Buckstein, J. He, H. Rubin, Characterization of nucleotide pools as a function of physiological state in Escherichia coli. *J Bacteriol* **190**, 718-726 (2008).
